# Semicarbazide and thiosemicarbazide containing butylated hydroxytoluene moiety: new potential antioxidant additives for synthetic lubricating oil†

**DOI:** 10.1039/d0ra10626g

**Published:** 2021-02-10

**Authors:** Syabilah Sazeli, Amit R. Nath, Mohd Hafiz Ahmad, N. W. M. Zulkifli, Mohd Rafie Johan, Wageeh A. Yehye, Lee Hwei Voon

**Affiliations:** Nanotechnology & Catalysis Research Centre (NANOCAT), Universiti Malaya Block 3A, Inst. for Advanced Studies 50603 Kuala Lumpur Malaysia syabilahsazeli@hotmail.com amit_cu.chem@yahoo.com hafiz99414@yahoo.com wdabdoub@yahoo.com leehweivoon@um.edu.my; Department of Mechanical Engineering, Universiti Malaya 50603 Kuala Lumpur Malaysia nurinmz@um.edu.my

## Abstract

New multipotent antioxidants (MPAOs), namely semicarbazides and thiosemicarbazides bearing thiolated butylated hydroxytoluene (BHT), were synthesized. The antioxidant activity of the synthesized compounds was evaluated using *in vitro* DPPH assay. Compounds containing thiosemicarbazides (5a′–h′) were found more active in free radical scavenger than semicarbazides (5a–h). Among the other compounds, compound 5f′ (IC_50_ of 25.47 ± 0.42 μM) showed the best antioxidant activity against DPPH radicals compared to standard antioxidant butylated hydroxytoluene (BHT). Based on DPPH results, compound 5f′ and its corresponding semicarbazide 5f were blended into trimethylolpropane trioleate (TMPTO) and isothermal differential scanning calorimetry (DSC) was carried out for the investigation of oxidative stability. At 125 °C isothermal DSC, TMPTO with 0.25 wt% of 5f′ showed 1.5 times higher oxidation stability than its corresponding semicarbazide 5f and was 2 times better than BHT. It was anticipated that due to the strong auto-synergistic effect, compound 5f′ showed promising oxidative stability to TMPTO by protecting from pre-mature oxidative degradation.

## Abstract 2

New multipotent antioxidants (MPAOs), namely semicarbazides and thiosemicarbazides bearing thiolated butylated hydroxytoluene (BHT), were synthesized.

## Graphical abstract

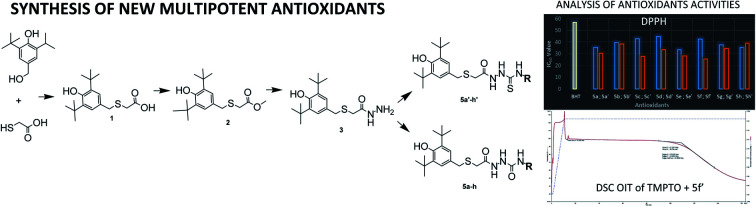

## Introduction

Premature oxidation is one of the major hindrances to develop oxidative and thermally stable synthetic lubricants. For the last few years, growing ecological alarms and expanding preventive etiquettes also have encouraged the enhancement of environmental-friendly lubricants worldwide. Therefore, synthetic ester-based biodegradable lubricant oils are a class of compound that need extra attention. The most commonly used ester-based lubricant is trimetylolpropane trioleate (TMPTO), a petrochemical derivative with naturally derived oleic acid.^1–3^ It has outstanding lubricity, low-temperature properties, high viscosity index, and improved oxidative resistance compared to vegetable oil thus promoting it to be a competitive green lubricant.

Though TMPTO provides superior lubricant properties to mineral oil, its wide application was restricted due to its poor oxidative stability towards oil from premature oxidation or auto-oxidation.^1^ Therefore, the development of antioxidants with better oxidative stability properties and less toxicity appeared to be one of the reasons of searching for new potential antioxidants. In the history of the development of antioxidants, many scientists have proven semicarbazide and thiosemicarbazide derivatives as a superior oxidation inhibitors. This type of compound has rising interests in the research group because of its multi-donor ligands. They had a wide range of pharmacological activities not only as antioxidant, but also as antifungal, antibacterial, antitumor, antimalarial, antiviral, antimicrobial, anti-tubecular agents, which made it biologically active and nontoxic compounds.^4–7^

On the other hand, BHT is the most common primary synthetic antioxidant which has been used in a wide range of application until today. Current research found that BHT could be combining with other secondary antioxidant to obtain an excellent oxidative stability for synthetic ester based lubricant.^8–10^ Antioxidant synergism is the subsequent impact of utilizing at least two different type of antioxidants together conveys more noteworthy oxidation soundness than that of any individual antioxidant functions.^11^ Synergistic antioxidants frameworks compromise convincing answers for issues where a single antioxidant is deficient to offer satisfactory outcomes.^12^ The mixture of various antioxidants in definite proportion could perform in a decent synergistic impact where oxidation stability is superior to the exclusively utilized antioxidants.^8,13^

Organosulfur compounds are frequently used in the lubricant compositions with BHT to have better synergistic impact since it is essential additives for antioxidant, antiwear and extreme pressure properties. Though, the least amount of sulfur-containing additive should be applied in advanced engine oil formulation according to the current environmental and economic constraint. In fact, the use of several antioxidants in lubricant formulation may raise the environmental issue and increase the cost. Therefore, multi-functional antioxidant has become more popular to cut down the number of several antioxidants in lubricant composition. Since multifunctional antioxidants can possess several antioxidant functional groups in their structure, which can provide auto-synergistic antioxidant system by exhibiting radical scavenging and peroxide decomposing properties.^12,14,15^

So, we envisioned to assemble several antioxidant functions in one structure by incorporating BHT moiety into semicarbazide and thiosemicarbazide compounds to obtain better antioxidant auto-synergistic effect. This article deals with the preparation and evaluation of new series of semicarbazide and its corresponding thiosemicarbazide derivatives bearing BHT moiety for their antioxidant activities using DPPH assays and DSC. BHT moiety has been incorporated into semicarbazide and thiosemicarbazide through thioether bridge to obtain multifunctional antioxidant which can offer better auto-synergistic antioxidant system. To our acquaintance, this is the first report discussing the comparative antioxidant activity between semicarbazide and thiosemicarbazide derivatives. Thus, this study would help researchers to have deep understanding on potential between multifunctional semicarbazide and thiosemicarbazide derivatives as antioxidant inhibitor for future global lubricant oxidative stability industry perspective.

## Experimental methods

### General reagents and instruments

All materials and solvents were purchased in analytical grade from commercial suppliers and used without further purification. Toluene was dried over molecular sieves 4A (Sigma-Aldrich) prior to use. BHT and DPPH were purchased from Sigma-Aldrich. Merck TLC aluminium sheets (Silica gel 60 F254) were used for thin layer chromatography. All synthesized target compounds were characterized using FTIR, NMR, LCMS, and melting point instrument. An infrared spectrum was recorded on a PerkinElmer, FTIR-Spectrum 400 spectrometer at wave number from 4000–400 cm^−1^. 1D and 2D analysis of nuclear magnetic resonance (NMR) spectra were obtained on Bruker Advance III 600 MHz. Spectra are reported in units of ppm on the scale, relative to chloroform and DMSO. The coupling constants are given in Hz. HR-mass spectra (ESI†) were obtained with 6550 i-Funnel Q-TOF LC-MS. Melting points were determined with Mel-Temp II melting point apparatus.

### Preparation of acid hydrazide

**Scheme 1:**
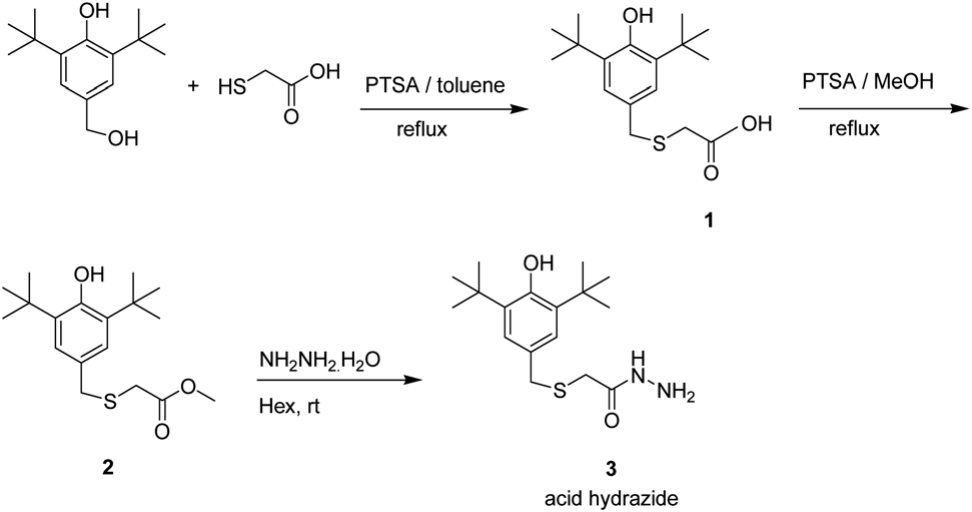
Preparation of acid hydrazide, 3.

The 2-((3,5-di-*tert*-butyl-4-hydroxybenzyl)thio) acetohydrazide was synthesized according to previously described method shown in Scheme 1.^16^

### Preparation of semicarbazides and thiosemicarbazides: general synthesis procedure

To a solution of *S*-(3,5-di-*tert*-butyl-4-hydroxybenzyl) thioglycolic acid hydrazide (3; 0.3241 g, 1 mmol), dry toluene (5 mL) was added isocyanate (1 mmol), and the reaction mixture was stirred at room temperature for 2 hours until the reaction completed (monitored by thin-layer chromatography). The white precipitate was collected by filtration, washed with water and boiled hexane, and dried at 50 °C. The same procedure is applied to respective isothiocyanate group of reactants.

#### 2-(2-(3,5-Di-*tert*-butyl-4-hydroxybenzylthio)acetyl)-*N*-phenyl hydrazinecarboamide, 5a

Yield: 0.42 g (95%); white solid; mp 151 °C; FTIR (ATR): 3619, 3252, 2960, 1605 cm^−1^; ^1^H NMR (600 MHz, DMSO-*d*_6_): *δ* = 9.83 (s, 1H), 8.72 (s, 1H), 8.13 (s, 1H), 7.46 (d, *J* = 6 Hz, 2H), 7.26 (t, *J* = 6 Hz, 2H), 7.08 (s, 2H), 6.96 (t, *J* = 6 Hz, 1H), 6.89 (s, 1H), 3.79 (s, 2H), 3.13 (s, 2H), 1.38 (s, 18H); ^13^C NMR (150 MHz, DMSO-*d*_6_): *δ* = 169.49, 155.68, 153.32, 140.06, 139.63, 129.12, 128.92, 125.73, 122.38, 118.90, 36.56, 34.95, 32.92, 30.83; HRMS (Q-TOF): *m*/*z* [M + Na]^+^ = 466.2161, calcd for C_24_H_33_NaN_3_O_3_S^+^ 466.2135.

#### 2-(2-(3,5-Di-*tert*-butyl-4-hydroxybenzylthio)acetyl)-*N*-(4-fluorophenyl)hydrazinecarboamide, 5b

Yield: 0.44 g (96%); white solid; mp 160–162 °C; FTIR (ATR): 3634, 3292–3344, 2871–2955, 1656 cm^−1^; ^1^H NMR (600 MHz, DMSO-*d*_6_): *δ* = 9.81 (s, 1H), 8.76 (s, 1H), 8.15 (s, 1H), 7.47 (m, 2H), 7.10 (m, 2H), 7.07 (s, 2H), 6.89 (s, 1H), 3.78 (s, 2H), 3.12 (s, 2H), 1.37 (s, 18H); ^13^C NMR (150 MHz, DMSO-*d*_6_): *δ* = 169.49, 158.64, 157.06, 153.31, 139.64, 136.40, 128.92, 120.70, 115.66, 115.52, 36.55, 34.94, 32.93, 30.82; HRMS (Q-TOF): *m*/*z* [M + H]^+^ = 462.2225, calcd for C_24_H_33_N_3_O_3_SF^+^ 462.2221.

#### 2-(2-(3,5-Di-*tert*-butyl-4-hydroxybenzylthio)acetyl)-*N*-4-(cyanophenyl)hydrazinecarboamide, 5c

Yield: 0.42 g (91%); white solid; mp 141–148 °C; FTIR (ATR): 3589–3649, 3337, 2956–3025, 2234, 1601 cm^−1^; ^1^H NMR (600 MHz, DMSO-*d*_6_): *δ* = 9.89 (s, 1H), 9.27 (b, 1H), 8.43 (s, 1H), 7.71 (d, *J* = 6 Hz, 2H), 7.65 (d, *J* = 6, 2H), 6.88 (s, 1H), 3.78 (s, 2H), 3.14 (s, 2H), 1.38 (s, 18H); ^13^C NMR (150 MHz, DMSO-*d*_6_): *δ* = 169.51, 155.24, 153.33, 144.66, 139.64, 133.64, 128.89, 125.73, 119.75, 118.74, 103.86, 36.53, 34.95, 32.87, 30.82; HRMS (Q-TOF): *m*/*z* [M + H]^+^ = 469.2265, calcd for C_25_H_33_O_3_N_4_S^+^ 469.2268.

#### 2-(2-(3,5-Di-*tert*-butyl-4-hydroxybenzylthio)acetyl)-*N*-(3-methoxyphenyl)hydrazinecarboamide, 5d

Yield: 0.44 g (93%); white solid; mp 134–136 °C; FTIR (ATR): 3624, 3288, 2961, 1683 cm^−1^; ^1^H NMR (600 MHz, DMSO-*d*_6_): *δ* = 9.82 (b, 1H), 8.72 (b, 1H), 8.12 (b, 1H), 7.16 (m, 1H), 7.08 (s, 2H), 6.98 (d, *J* = 6 Hz, 1H), 6.88 (s, 1H), 6.54 (dd, *J* = 6 *J* = 6 Hz, 1H), 3.79 (s, 2H), 3.71 (s, 3H), 3.13 (s, 2H), 1.38 (s, 18H); ^13^C NMR (150 MHz, DMSO-*d*_6_): *δ* = 169.49, 158.64, 157.06, 153.31, 139.64, 136.40, 128.92, 120.70, 115.59, 36.55, 34.94, 32.93, 30.82; HRMS (Q-TOF): *m*/*z* [M + H]^+^ = 475.2470, calcd for C_25_H_37_O_4_N_3_S^+^ 475.2442.

#### 2-(2-(3,5-Di-*tert*-butyl-4-hydroxybenzylthio)acetyl)-*N*-(2,4-dichlorophenyl)hydrazinecarboamide, 5e

Yield: 0.46 g (90%); white solid; mp 104–106 °C; FTIR (ATR): 3644, 3184–3332, 2862–2956, 1591 cm^−1^; ^1^H NMR (600 MHz, DMSO-*d*_6_): *δ* = 10.00 (s, 1H), 8.91 (s, 1H), 8.33 (b, 1H), 8.11 (d, 3*J* = 6 Hz 1H), 7.62 (d, 4*J* = 0 Hz 1H), 7.37 (dd, 3*J* = 6, 4*J* = 0 Hz 1H), 7.07 (s, 2H), 6.89 (s, 1H), 3.78 (s, 2H), 3.13 (s, 2H), 1.37 (s, 18H); ^13^C NMR (150 MHz, DMSO-*d*_6_): *δ* = 169.40, 155.03, 153.33, 139.63, 135.50, 129.06, 128.84, 128.14, 126.80, 125.74, 123.28, 122.57, 36.52, 34.94, 32.65, 30.81; HRMS (Q-TOF): *m*/*z* [M + Na]^+^ = 510.1373, calcd for C_22_H_31_O_3_NaN_3_SCl_2_^+^ 510.1336.

#### 2-(2-(3,5-Di-*tert*-butyl-4-hydroxybenzylthio)acetyl)-*N*-(1-naphthyl) hydrazinecarboamide, 5f

Yield: 0.44 g (89%); white solid; mp 121–123 °C; FTIR (ATR): 3639, 3200–3302, 2873–2961, 1610 cm^−1^; ^1^H NMR (600 MHz, DMSO-*d*_6_): *δ* = 9.96 (s, 1H), 8.79 (s, 1H), 8.43 (s, 1H), 8.06 (d, 4*J* = 12 Hz, 1H), 7.92 (m, 1H), 7.80 (b, 1H), 7.67 (d, 3*J* = 6 Hz, 1H), 7.53 (m, 2H), 7.46 (t, 3*J* = 6, 3*J* = 6 Hz, 1H), 7.08 (s, 2H), 6.90 (s, 1H), 3.80 (s, 2H), 3.16 (s, 2H), 1.36 (s, 18H); ^13^C NMR (150 MHz, DMSO-*d*_6_): *δ* = 169.60, 156.42, 153.32, 139.69, 134.58, 134.19, 128.90, 128.74, 126.41, 126.21, 126.16, 125.74, 124.25, 122.38, 119.42, 36.59, 34.92, 32.91, 30.81; HRMS (Q-TOF): *m*/*z* [M + H]^+^ = 494.2473, calcd for C_28_H_36_O_3_N_3_S^+^ 494.2472.

#### 2-(2-(3,5-Di-*tert*-butyl-4-hydroxybenzylthio)acetyl)-*N*-(allyl)hydrazinecarboamide, 5g

Yield: 0.37 g (90%); white solid; mp 127–129 °C; FTIR (ATR): 3619, 3436.3–3515, 3139, 2867–2956, 1675 cm^−1^; ^1^H NMR (600 MHz, DMSO-*d*_6_): *δ* = 9.63 (s, 1H), 7.86 (s, 1H), 7.06 (s, 2H), 6.86 (s, 1H), 6.47 (t, 3*J* = 6, 3*J* = 6 Hz, 1H), 5.80 (m, 1H), 5.15 (dd, 3*J* = 18, 2*J* = 0 Hz, 1H), 5.03 (dd, 3*J* = 12, 2*J* = 0 Hz, 1H), 3.75 (s, 2H), 3.66 (t, 3*J* = 6, 3*J* = 6 Hz, 2H), 3.08 (s, 2H), 1.38 (s, 18H); ^13^C NMR (150 MHz, DMSO-*d*_6_): *δ* = 169.39, 158.27, 153.30, 139.62, 136.75, 128.96, 125.68, 114.94, 41.94, 36.62, 34.94, 32.99, 30.84; HRMS (Q-TOF): *m*/*z* [M + Na]^+^ = 430.2154, calcd for C_21_H_33_O_3_NaN_3_S^+^ 430.2135.

#### 2-(2-(3,5-Di-*tert*-butyl-4-hydroxybenzylthio)acetyl)-*N*-butyl hydrazinecarboamide, 5h

Yield: 0.37 g (87%); white solid; mp 82–84 °C; FTIR (ATR): 3375, 3132, 2849–2917, 1635 cm^−1^; ^1^H NMR (600 MHz, DMSO-*d*_6_): *δ* = 9.60 (s, 1H), 7.75 (s, 1H), 7.06 (s, 2H), 6.87 (s, 1H), 6.26 (t, 3*J* = 6, 3*J* = 6 Hz, 1H), 3.75 (s, 2H), 3.07 (s, 2H), 3.01 (m, 2H), 1.37 (s, 18H), 1.26 (m, 2H), 0.87 (t, 3*J* = 6, 3*J* = 6 Hz, 3H); ^13^C NMR (150 MHz, DMSO-*d*_6_): *δ* = 169.35, 158.41, 153.30, 139.61, 128.93, 125.69, 39.30, 36.59, 34.93, 32.92, 32.42, 30.82, 19.88, 14.17; HRMS (Q-TOF): *m*/*z* [M + H]^+^ = 424.2637, calcd for C_22_H_38_O_3_N_3_S^+^ 424.2628.

#### 2-(2-(3,5-Di-*tert*-butyl-4-hydroxybenzylthio)acetyl)-*N*-phenyl hydrazinecarbothioamide, 5a′

Yield: 0.43 g (94%); white solid; mp 126–128 °C; FTIR (ATR): 3634, 3144, 2956, 1670, 1225 cm^−1^; ^1^H NMR (600 MHz, DMSO-*d*_6_): *δ* = 10.10 (s, 1H), 9.67 (s, 1H), 9.60 (b, 1H), 7.45 (s, 2H), 7.34 (t, 3*J* = 6, 3*J* = 6 Hz, 1H), 7.17 (t, 3*J* = 6, 3*J* = 6 Hz, 1H), 7.08 (s, 2H), 6.90 (s, 1H), 3.79 (s, 2H), 3.17 (s, 2H), 1.38 (s, 18H); ^13^C NMR (150 MHz, DMSO-*d*_6_): *δ* = 181.39, 169.38, 153.34, 139.66, 139.55, 128.87, 128.59, 126.09, 125.70, 125.51, 36.67, 34.95, 33.35, 30.84; HRMS (Q-TOF): *m*/*z* [M + H]^+^ = 460.2096, calcd for C_24_H_34_O_3_N_3_S_2_^+^ 460.2087.

#### 2-(2-(3,5-Di-*tert*-butyl-4-hydroxybenzylthio)acetyl)-*N*-(4-fluorophenyl)hydrazinecarbothioamide, 5b′

Yield: 0.45 g (95%); white solid; mp 109–111 °C; FTIR (ATR): 3624, 3139–3283, 2956, 1670, 1220 cm^−1^; ^1^H NMR (600 MHz, CDCl_3_): *δ* = 9.47 (b, 1H), 8.54 (b, 1H), 8.11 (s, 1H), 7.39 (m, 2H), 7.12 (s, 2H), 7.11 (d, *J* = 6 Hz, 2H), 5.24 (s, 1H), 3.85 (s, 2H), 3.27 (s, 2H), 1.44 (s, 18H); ^13^C NMR (150 MHz, CDCl_3_): *δ* = 166.22, 162.09, 160.46, 153.29, 136.47, 132.47, 127.07, 126.92, 125.86, 116.64, 116.49, 37.75, 34.35, 33.76, 30.23; HRMS (Q-TOF): *m*/*z* [M + Na]^+^ = 500.1827, calcd for C_24_H_32_O_2_NaN_3_S_2_F^+^ 500.1812.

#### 2-(2-(3,5-Di-*tert*-butyl-4-hydroxybenzylthio)acetyl)-*N*-(4-cyanophenyl)hydrazinecarbothioamide, 5c′

Yield: 0.43 g (90%); white solid; mp 152–154 °C; FTIR (ATR): 3634, 3127–3205, 2956, 2229, 1600, 1240 cm^−1^; ^1^H NMR (600 MHz, CDCl_3_): *δ* = 9.81 (b, 2H), 8.93 (b, 1H), 7.66 (d, *J* = 6 Hz, 2H), 7.53 (d, *J* = 6 Hz, 2H), 7.03 (s, 2H), 5.17 (s, 1H), 3.76 (s, 2H), 3.25 (s, 2H), 1.34 (s, 18H); ^13^C NMR (150 MHz, CDCl_3_): *δ* = 179.79, 167.41, 153.42, 142.01, 136.63, 133.03, 126.59, 125.78, 122.55, 118.55, 107.53, 37.78, 34.36, 33.97, 30.22; HRMS (Q-TOF): *m*/*z* [M + H]^+^ = 485.2043, calcd for C_25_H_33_O_2_N_4_S_2_^+^ 485.2039.

#### 2-(2-(3,5-Di-*tert*-butyl-4-hydroxybenzylthio)acetyl)-*N*-(3-methoxyphenyl)hydrazinecarbothioamide, 5d′

Yield: 0.46 g (94%); white solid; mp 109–111 °C; FTIR (ATR): 3634, 3283–3332, 2834–2956, 1674, 1162 cm^−1^; ^1^H NMR (600 MHz, DMSO-*d*_6_): *δ* = 10.09 (b, 1H), 9.68 (s, 1H), 9.54 (b, 1H), 7.24 (t, 3*J* = 6, 3*J* = 6 Hz, 1H), 7.17 (s, 1H), 7.08 (s, 2H), 7.04 (dd, 3*J* = 6, 4*J* = 6 Hz, 1H), 6.89 (s, 1H), 6.74 (d, 3*J* = 6 Hz, 1H), 3.79 (s, 2H), 3.74 (s, 1H), 3.17 (s, 2H), 1.38 (s, 18H); ^13^C NMR (150 MHz, DMSO-*d*_6_): *δ* = 181.20, 169.37, 159.50, 153.34, 140.67, 139.66, 129.37, 128.86, 125.71, 110.93, 55.57, 36.68, 34.95, 33.33, 30.83; HRMS (Q-TOF): *m*/*z* [M + H]^+^ = 491.2245, calcd for C_25_H_36_O_3_N_3_S_2_^+^ 491.2293.

#### 2-(2-(3,5-Di-*tert*-butyl-4-hydroxybenzylthio)acetyl)-*N*-(2,4-dichlorophenyl)hydrazinecarbothioamide, 5e′

Yield: 0.51 g (96%); white solid; mp 131–136 °C; FTIR (ATR): 3634, 3174–3278, 2965, 1651, 1275 cm^−1^; ^1^H NMR (600 MHz, DMSO-*d*_6_): *δ* = 10.19 (b, 1H), 9.90 (b, 1H), 9.48 (b, 1H), 7.67 (s, 1H), 7.43 (d, 3*J* = 6 Hz, 2H), 7.07 (s, 2H), 6.89 (s, 1H), 3.78 (s, 2H), 3.16 (s, 2H), 1.37 (s, 18H); ^13^C NMR (150 MHz, DMSO-*d*_6_): *δ* = 182.34, 169.41, 153.34, 139.66, 136.35, 132.86, 132.49, 131.94, 129.28, 128.85, 127.78, 125.69, 36.69, 34.94, 33.24, 30.83; HRMS (Q-TOF): *m*/*z* [M + Na]^+^ = 526.1147, calcd for C_22_H_31_O_2_NaN_3_S_2_Cl_2_^+^ 526.1138.

#### 2-(2-(3,5-Di-*tert*-butyl-4-hydroxybenzylthio)acetyl)-*N*-1-naphthyl hydrazine carbothioamide, 5f′

Yield: 0.47 g (92%); white solid; mp 119–121 °C; FTIR (ATR): 3644, 3166–3258, 2956, 1698 cm^−1^; ^1^H NMR (600 MHz, CDCl_3_): *δ* = 9.79 (b, 1H), 9.04 (b, 1H), 8.54 (s, 1H), 7.92 (m, 1H), 7.82 (m, 1H), 7.76 (d, 3*J* = 12 Hz, 1H), 7.58 (d, 3*J* = 12 Hz, 1H), 7.46 (m, 1H), 7.45 (s, 1H), 7.42 (m, 1H), 7.00 (s, 2H), 5.08 (s, 1H), 3.72 (s, 2H), 3.07 (s, 2H), 1.31 (s, 18H); ^13^C NMR (150 MHz, CDCl_3_): *δ* = 179.16, 165.29, 153.24, 136.29, 134.51, 132.15, 129.58, 128.57; 128.53, 127.27, 126.87, 126.83, 125.87, 125.63, 124.92, 122.10, 37.62, 34.31, 33.69, 30.24; HRMS (Q-TOF): *m*/*z* [M + H]^+^ = 510.2241, calcd for C_28_H_36_O_2_N_3_S_2_^+^ 510.2243.

#### 2-(2-(3,5-Di-*tert*-butyl-4-hydroxybenzylthio)acetyl)-*N*-(allyl) hydrazine carbothioamide, 5g′

Yield: 0.47 g (85%); white solid; mp 113–124 °C; FTIR (ATR): 3619, 3439–3512, 3141, 2868–2956, 1674, 1230 cm^−1^; ^1^H NMR (600 MHz, DMSO-*d*_6_): *δ* = 9.88 (*s*, 1H), 9.32 (*s*, 1H), 8.05 (*s*, 1H), 7.07 (*s*, 2H), 6.88 (*s*, 1H), 5.82 (*m*, 1H), 5.13 (dd, 3*J* = 18, 2*J* = 6 Hz, 1H), 5.04 (dd, 3*J* = 12, 2*J* = 0 Hz, 1H), 4.10 (*s*, 2H), 3.75 (*s*, 2H), 3.11 (*s*, 2H), 1.38 (*s*, 18H); ^13^C NMR (150 MHz, DMSO-*d*_6_): *δ* = 182.35, 169.39, 153.33, 139.64, 135.31, 128.87, 125.68, 115.76, 46.31, 36.66, 34.94, 33.27, 30.84; HRMS (Q-TOF): *m*/*z* [M + H]^+^ = 424.2110, calcd for C_21_H_34_O_2_N_3_S_2_^+^ 424.2117.

#### 2-(2-(3,5-Di-*tert*-butyl-4-hydroxybenzylthio)acetyl)-*N*-(butyl) hydrazine carbothioamide, 5h′

Yield: 0.41 g (93%); white solid; mp 122–127 °C; FTIR (ATR): 3619–3639, 3346, 3215–3273, 2868–2956, 1664, 1225 cm^−1^; ^1^H NMR (600 MHz, DMSO-*d*_6_): *δ* = 9.83 (*s*, 1H), 9.20 (*s*, 1H), 7.82 (*s*, 1H), 7.06 (*s*, 2H), 6.89 (*s*, 1H), 3.75 (*s*, 2H), 3.42 (*d*, 3*J* = 6 Hz, 2H), 3.10 (*s*, 2H), 1.46 (*m*, 2H), 1.38 (*s*, 18H), 1.25 (*m*, 2H), 0.88 (*t*, 3*J* = 6, 3*J* = 6 Hz, 3H); ^13^C NMR (150 MHz, DMSO-*d*_6_): *δ* = 192.18, 169.49, 158.64, 157.06, 153.31, 139.65, 128.86, 125.69, 43.80, 36.63, 34.94, 33.19, 31.32, 30.83, 19.88, 14.25; HRMS (Q-TOF): *m*/*z* [M + H]^+^ = 440.2424, calcd for C_22_H_38_O_2_N_3_S_2_^+^ 440.2400.

### Antioxidant assay (DPPH scavenging assay)

DPPH radical scavenging assay had been carried out according to protocol reported by Ahmad *et al.* with some modifications.^17^ Around 0.2 mM of DPPH solution in methanol was added to various concentrations; 10, 20, 40 and 60 μM of antioxidant samples. A negative control (no sample) is using same concentration of DPPH solution. Each assay was carried out in triplicates. Then, the mixtures were vortexed and incubated in dark for 60 min at room temperature. Absorbance at 517 nm for each sample was measured. BHT was used as positive control. The free radical scavenging activity of the compounds had calculated as a percentage of radical inhibition by using the formula:
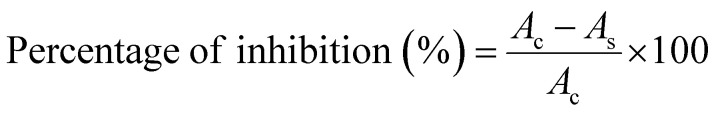
*A*_s_ = absorbance of the compounds/positive control, and *A*_c_ = absorbance of control (DPPH solution and methanol).

Percentage of DPPH inhibition of each compounds were plotted against extract concentration in order to determine the concentration required to achieve 50% inhibition (IC_50_) of DPPH radical.

### Differential scanning calorimetry

The antioxidant effect of hindered phenols and their synergistic effect with secondary antioxidant were demonstrated in thermo-oxidative stability test by differential scanning calorimetry according to ASTM D 3895 with some modification. Free radical contents of the oxidized oils were determined to explain the antioxidant synergistic effect of hindered phenols with secondary antioxidant compounds.

The DSC experiment involves heating a thin film of oil sample on an aluminium pan in an oxygen atmosphere and detecting the exotherm corresponding to the onset of rapid and accelerating oxidation. This was carried out in isothermal temperature programmed mode where the temperature was held constant and the elapsed time to the onset of oxidation (oxidative induction time) was measured. A longer oxidative induction time indicate an improvement of oxidation stability. DSC test conditions as below:Sample weight = 10 mgGas flow rate = 20 mL min^−1^Gas pressure = 2 barTemperature programmed = 10 °C min^−1^Isothermal temperature = 125 °CAntioxidant composition range = 0.25 wt%

The induction periods from the isothermal experiments were obtained by extrapolation of the front edge of the exothermic peak to the baseline.

## Results and discussion

### Chemistry

**Scheme 2:**
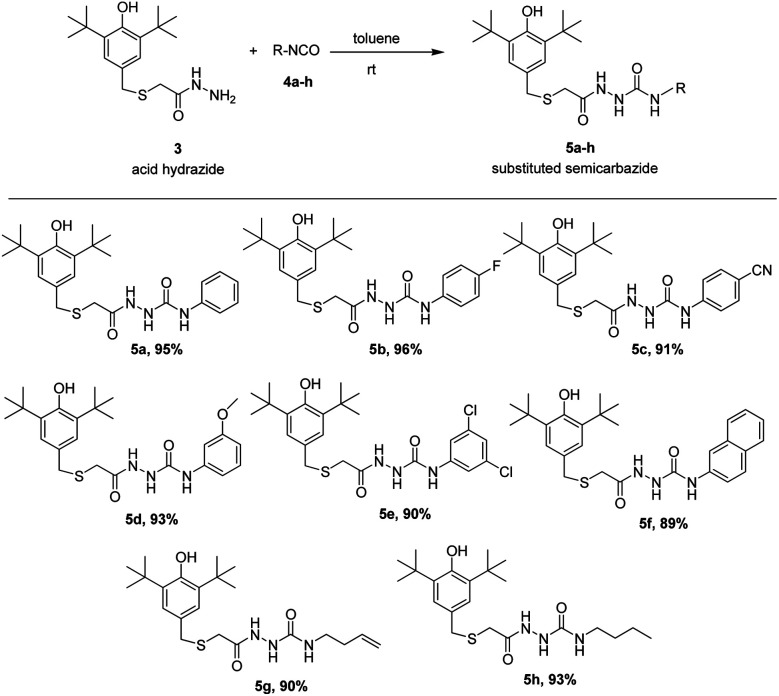
Preparation of substituted semicarbazides, 5a–h.

The preparations of the starting material *S*-(3,5-di-*tert*-butyl-4-hydroxybenzyl)thioglycolic acid hydrazide, 3, a prerequisite precursor, are outlined in Scheme 1.^18,19^ Reaction between acid hydrazide and isocyanates is a straightforward reaction at room temperature that has been reported by our research group.^18^ Herein, we carried out this reaction between prepared acid hydrazide 3 and isocyanates 4a–h at ambient temperature utilizing dry toluene solvent to give corresponding semicarbazides. Interestingly, the reaction started to form visible products almost instantly giving very good yield, (89–96%). TLC was used as indicator upon reaction completion. The results of the reaction are summarized in Scheme 2.

Synthesized compounds were confirmed by ^1^H NMR, ^13^C NMR, high-resolution Q-TOF mass analysis. Tetramethylsilane was used as an internal reference. 600 MHz and 150 MHz NMR spectrometer were used for ^1^H NMR and ^13^C NMR respectively. The IR spectroscopy of semicarbazides clearly showed an O–H stretching sharp band at range 3619–3644 cm^−1^ and the presence of C=O stretching bands at 1703–1715 cm^−1^. A strong band at 3242–3344 cm^−1^ was assigned to the N–H stretching whereas C–H aliphatic bands appeared at 2955–2961 cm^−1^. The mass spectrometry analysis of the products also confirmed the molecular weight of compounds 5a–h. HRMS (ESI†) of each compound had stated in experimental section.

The ^1^H NMR and ^13^C NMR of compound 5a–h were quite similar to each other. The differences between them in the spectra were accorded to the peak and chemical shift of the different substituents on the hydrazides. Basically, each semicarbazides will have a singlet peak at 1.36–1.38 ppm due to eighteen protons of 2-C(C***H***_3_)_3_. Singlet peak at the region between 6.87–6.90 ppm attributed to free phenolic hydroxyl. H-7 and H-8 of thioether in 5a–h exhibited two separate singlet peaks in the region 3.75–3.80 ppm and 3.07–3.16 ppm respectively. H-3 and H-5 of the compounds appeared as singlet at 7.06–7.08 ppm which were assigned to the two symmetrical aromatic ring protons. All compounds exhibited a broad signal at 9.60–10.00 ppm, 7.75–8.76 ppm and 6.26–9.27 ppm referring to NH-1, NH-2 and NH-3 respectively. ^13^C NMR spectra of 5a–h confirmed the presence of carbonyl from semicarbazides –NH(**C=O**)NH at C-10 in the range of 155.03–158.41 ppm.

**Scheme 3:**
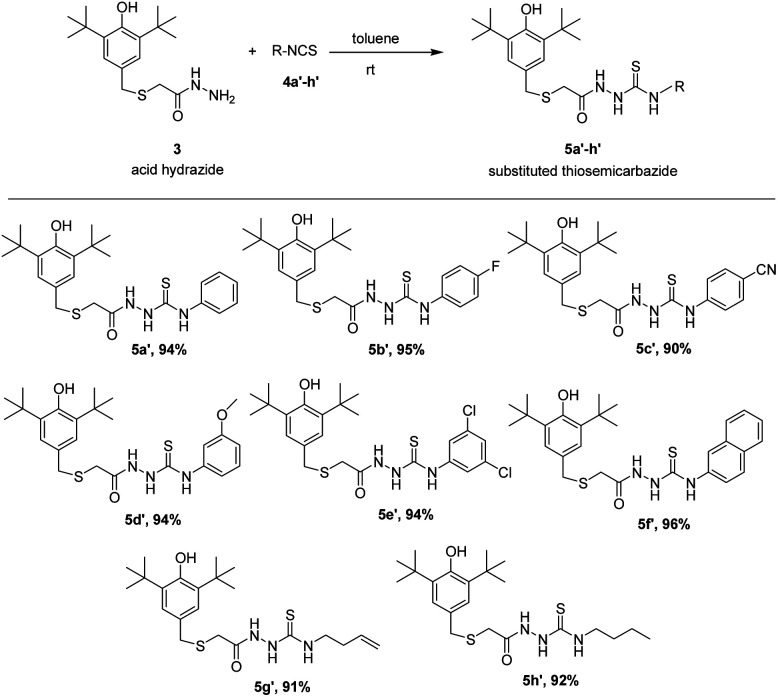
Preparation of substituted thiosemicarbazides, 5a′–h′.

As seems in semicarbazides, thiosemicarbazides group were also successfully formed with the same method. Acid hydrazide 3 was exposed to suitable corresponding thiocyanates 4a′–h′ in toluene at room temperature giving very good yield (90–96%) under addition reaction. All the eight corresponding thiosemicarbazides, 5a′–h′ were synthesized and reported for the first time. The results of the reaction are summarized in Scheme 3. The spectroscopic features of 5a′–h′ were quite similar to the 5a–h corresponding compounds. In IR, a very strong band at the region around 1100–1200 cm^−1^ was assigned to the C=S stretching showing a confirmation that belongs to thiosemicarbazides' group.^20^

In ^1^H NMR and ^13^C NMR spectra, the major difference was only the chemical shift from N–H group due to neighbouring group and carbon at C=S group. ^1^H NMR spectra of 5a′–h′ showed three singlet peaks at 9.47–10.19, 8.11–9.68, and 7.82–9.90 ppm referring to NH-1, NH-2, and NH-3 respectively. It is believed that the chemical shift of NH-2 and NH-3 had deshielding effects due to the thio-neighbouring group, C=S. In ^13^C spectra, it proved that C-10 also had deshielding effect from sulphur making the region appeared at 160.49–182.35 ppm. Thus, confirmed the presence of thiocarbonyl from thiosemicarbazides –NH(**C=S**)NH group.

### Antioxidant evaluation by DPPH

All the compounds were carried out for the antioxidant evaluation test using *in vitro* method of 1,1-diphenyl-2-picryl-hydrazine (DPPH) assay. DPPH assay has been one of the most widely used for measurement of free radical scavenging ability of antioxidants due to its rapid, simple and inexpensive properties.^21^ Overall antioxidant capacity of the system can also be estimated with accuracy by this method.

**Fig. 1:**
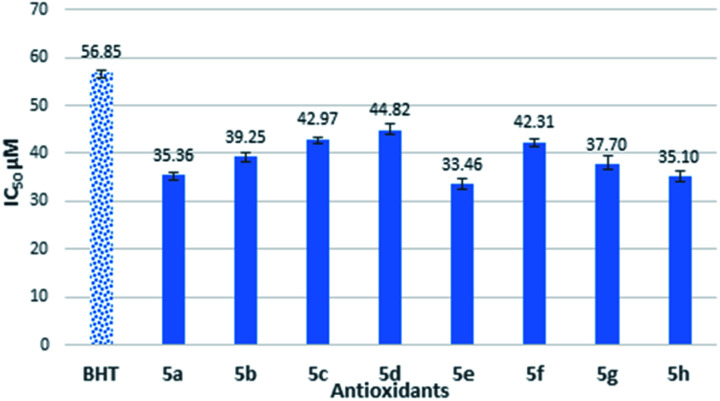
Antioxidant activity of synthesized compounds 5a–h by DPPH assay.

BHT was used as standard antioxidant. The delocalization of the electron in DPPH solution gives a deep violet color at 517 nm absorption. However, the color turns into yellow when antioxidant was added into the DPPH solution due to the reduction of divalent nitrogen (N) of DPPH by antioxidant molecule.^22^ The radical scavenging activity was expressed by the half maximal inhibitory concentration (IC_50_), the concentration needed to inhibit 50% of DPPH solution. Thus, the compound which has a lower IC_50_ value would shows better antioxidant properties. The free radical scavenging properties of the synthesized semicarbazides (5a–h) and thiosemicarbazides (5a′–h′) were investigated by the interaction between experimental compounds and 0.2 mM 1,1-diphenyl-2-picryl-hydrazine (DPPH) for 30 min. The antioxidant activity of synthesized semicarbazides (5a–h) obtained from DPPH assay is summarized in Fig. 1. All synthesized thiolated BHT bearing semicarbazides 5a–h showed better antioxidant activity in comparison with BHT. The decreasing order of IC_50_ values can be made from results in Fig. 1 as 5e > 5h > 5a > 5g > 5b > 5f > 5c > 5d > BHT.

**Fig. 2:**
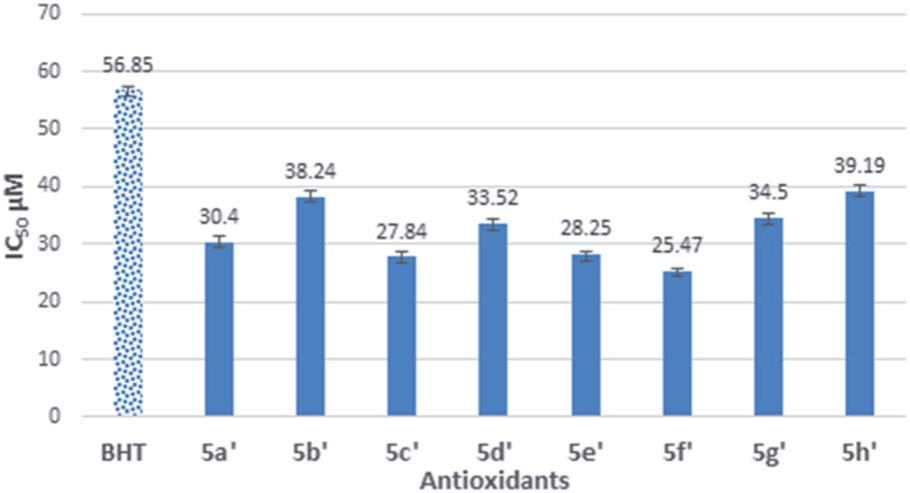
Antioxidant activity of synthesized compounds 5a′–h′ by DPPH assay.

The antioxidant activity of synthesized thiolated BHT bearing thiosemicarbazides (5a′–h′) is also depicted in Fig. 2. The moderate antioxidant activity was observed for all compounds. Interestingly, thiosemicarbazides bearing naphthyl, 4-cyano, 2,4-di-Cl substituents showed significant free radical scavenging activity. Free radical scavenging properties of synthesized compounds can be ordered from the obtained results as follows: 5f′ > 5c′ > 5e′ > 5a′ > 5d′ > 5g′ > 5b′ > 5h′ > BHT.

The IC_50_ values of novel compounds 5a–h and 5a′–h′ obviously indicated that all synthesized compounds have better radical scavenging activity than BHT. In general, the reason for synthesized semicarbazides has higher antioxidant activity than BHT is due to the combination of butylated hydroxy phenyl (BHP) moiety and semicarbazide moiety in a single structure. Semicarbazide moieties exhibit free radical scavenging properties due to the presence of an NH group, which can donate a hydrogen atom to the DPPH radical. After donating a hydrogen atom, compounds 5a–h exist in a radical form, and the radical electron could delocalize to the benzene ring to produce the stable resonance hybrid. The electron conjugation in the structure was stabilized the radical and was prevented it from participating in a destructive chemical reaction.

Thus, due to the antioxidant synergism, the compound 5a–h showed better free radical scavenging properties than standard antioxidant BHT. However, the synthesized substituted semicarbazides 5e, 5h, 5a and 5g showed IC_50_ value in the range of 33.46–37.70 μM. It is known that electron donating substituents enhance free radical scavenging properties than the electron withdrawing substituents on the benzene ring. The reason of this enhanced antioxidant properties might be attributed to the electron resonance effect of substituted benzene ring in radical 5e, 5h, 5a and 5g making the radical more stable in the presence of electron-donating groups. In contrast, semicarbazides containing highly electron withdrawing group –CN (5c), –F (5b) showed higher IC_50_ value 39.26 μM and 42.97 μM respectively. Again, though methoxy (–OMe) is an electron donating substituent but highest IC_50_ value was found for compound 5d. This may attribute to the fact that m-substituent showed only small resonance effect.^19^

**Scheme 4:**
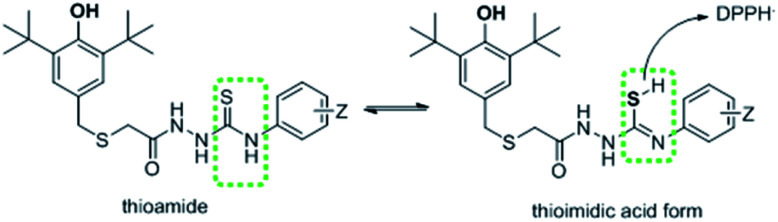
Possible thioamide–thioimidic acid tautomerism of thiosemicarbazides.

On the other hand, synthesized thiosemicarbazides 5a′–h′ showed better free radical scavenging properties in comparison with corresponding semicarbazides 5a–h. Thiosemicarbazides bearing naphthyl substituent 5f′ exhibited best antioxidant activity (25.47 ± 0.42 μM) among the thiosemicarbazides, followed by thiosemicarbazides containing –CN group 5c′ (27.84 ± 1.12 μM), 2,4-di-Cl 5e′ (28.25 ± 0.37 μM) and no substituents 5a′ (30.40 ± 0.61 μM). Strong delocalization of electron in naphthyl ring and steric hindrance might be attributed to the exhibition of enhanced the antioxidant activity of 5f′. Moreover, compounds with naphthalene ring were found in many earlier reports as potential free radical scavenger.^23–25^ Interestingly, thiosemicarbazides with strong electron withdrawing substituent such as cyano (–CN) showed lower IC_50_ value, whereas corresponding semicarbazides with electron withdrawing substituents found higher IC_50_ value. The reason of this phenomenon might be attributed to the thioamide–thioimidic acid tautomerism of synthesized thiosemicarbazides. Unlikely semicarbazides, thiosemicarbazides might reduce DPPH radical in thioimidic acidic form as shown in Scheme 4.

Gratifyingly, the IC_50_ value of thiosemicarbazide 5d′ (33.52 ± 0.99) support the thioimidic acid form of thiosemicarbazide is lower than its corresponding semicarbazide 5d. However, thiosemicarbazides with electron donating substituent such as butyl group (5h′) exhibited higher IC_50_ value (39.19 ± 0.75) though its corresponding 5h showed lower value. It is obvious from DPPH result that thiosemicarbazides with thiolated BHT were found better antioxidant properties in comparison with semicarbazides. There may have two possible reasons to obtain better scavenging properties of thiosemicarbazides: thioamide–thioimidic acid tautomerism and lower electronegativity of sulfur (2.58) than oxygen (3.44). Due to lower electronegativity of sulfur atom, thiol can easily release proton to DPPH radical in comparison of hydroxyl group (Scheme 4).^7,16,22,26^

**Scheme 5:**
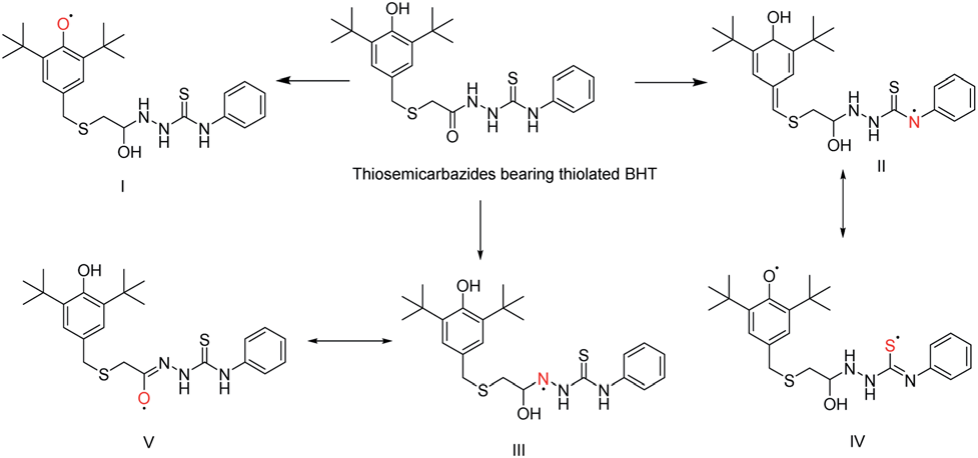
Possible radical formation of thiosemicarbazides bearing thiolated BHT.

In addition, it was found that the degree of conjugation in thioamides is considerably higher than amides which made it as stronger π-electron attractor.^27^ Nevertheless, thiosemicarbazides with thiolated BHT could convert into several radical by donating the proton to reduce DPPH radical. The possible radical formation of synthesized thiosemicarbazides can be drawn as Scheme 5. Thus, the multiple active site of donating proton to counteract the free radical has made our synthesized thiosemicarbazides more efficient antioxidant.

In summary, DPPH results have clarified the difference between C=O and C=S groups as well as the effect of substitution of electron-donating and – withdrawing on the scavenging of free radicals. With the exception of compound 5h and its corresponding 5h′, the IC_50_ values of thiosemicarbazides 5a′–g’ were lower than semicarbazides 5a–g group. It's clearly showed that most thiosemicarbazides groups, 5a′–g′ had a greater degree in antioxidant activity than their corresponding semicarbazides group of compounds, 5a–g. This study highlighted a novel finding that thiocarbonyl C=S in thiosemicarbazides (NH_2_–CS–NH–NH_2_) did play a role in antioxidant activity better than carbonyl in semicarbazides (NH_2_–CO–NH–NH_2_). Hence, thioamides carry better antioxidants than amides. Further investigation on structural behaviour is needed by computational and kinetic studies.

### Oxidation stability assessment with TMPTO

**Fig. 3:**
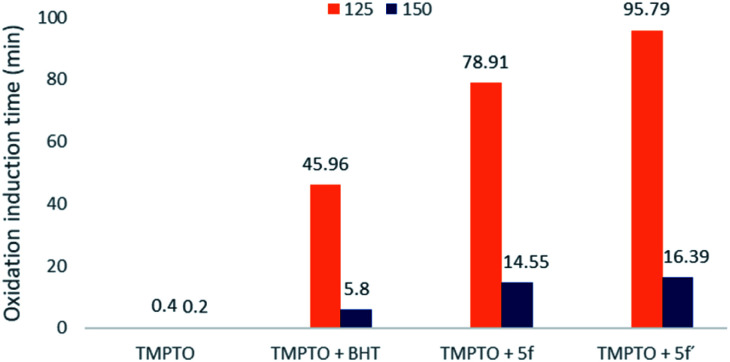
Oxidative induction time of TMPTO, TMPTO + BHT, TMPTO + 5f, and TMPTO + 5f′ at 150 °C and 125 °C isothermal temperature.

Based on DPPH result, the thiosemicarbazide compounds 5f′ was found as the best antioxidant properties. So, compound 5f′ and its corresponding semicarbazide compound 5f were carried out for oxidation stability evaluation of synthetic lubricant oil using differential scanning calorimetry (DSC) test. BHT was used as standard antioxiant since it is widely utilized in lubricant as a potential antioxidant. Trimethylolpropane trioleate (TMPTO) was used as synthetic ester-based lubricant. The compounds 5f′, 5f and BHT were blended into TMPTO with 0.25 wt%. Isothermal DSC was carried out with the blended experimental samples to obtain oxidative induction time (OIT). The longer OIT refers to extended oxidative stabiltiy of oil.^28^ This experiment was conducted at two different isothermal temperature: 150 °C and 125 °C. At first, blended oil was carried out isothermal DSC at 150 °C to evaluate OIT. Afterwards, same experiment was conducted at 125 °C isothermal temperature. Both OIT results are outlined in Fig. 3.

According to Fig. 3, the standard TMPTO was stable only for 0.4 min at 150 °C isothermal. Meanwhile, the OIT value of 0.25 wt% of 5f′ blended with TMPTO was extended to 16.39 min. But, 0.25 wt% of BHT blended with TMPTO was observed at 5.8 min OIT value. Here, it is clear that the incorporation of compound 5f′ with TMPTO extended its oxidation stability around 3 times more than 0.25 wt% of BHT.

Nevertheless, the highest oxidation stability of TMTPO blended with synthesized compound was observed for 0.25 wt% of 5f and 5f′ at 125 °C isothermal DSC. As seen in Fig. 3, the base oil of TMPTO at 125 °C isothermal DSC has the OIT value of 0.4 min. However, the addition of compounds 5f and 5f′ increased the OIT values to 78.91 and 95.79 min, respectively. So, both novel multipotent antioxidant compounds showed better oxidative stability of blended TMPTO (around 50%) than the commercial antioxidant BHT.

**Fig. 4:**
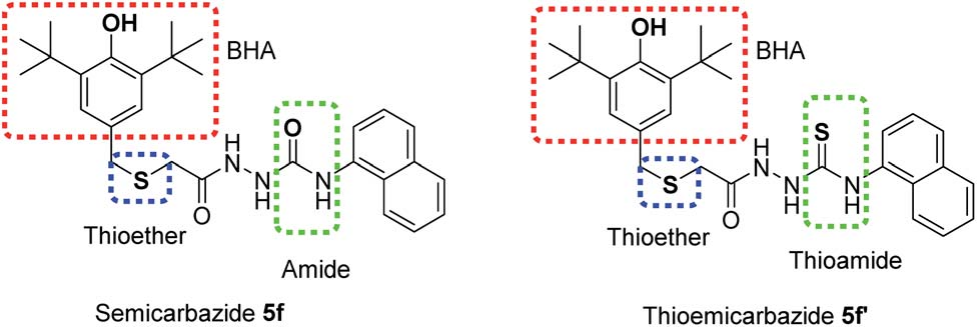
Important antioxidant functions in 5f and 5f′ for potential oxidative stability of lubricant oil.

These results illustrated that the novel MPAOs have a great potential as antioxidant additives for synthetic lubricating oil. Furthermore, it was observed that thiolated BHT containing thiosemicarbazide 5f′ exhibited stronger oxidative stability than semicarbazides with thiolated BHT 5f. This observation also supports the DPPH assay result where thiocarbonyls group give better antioxidant activity than carbonyls. This higher activity of this reporting compound 5f′ can be attributed to the presence of three different antioxidant functions in one structure: BHT as primary antioxidant, thioether and thioamide as secondary antioxidant or peroxide decomposer (Fig. 4). Whereas, semicarbazide 5f possesses two primary antioxidants: BHT and amide, one secondary antioxidant functions thioether. It was reported that antioxidants containing functional groups that can provide free radical scavenger activity and peroxide decomposer moiety exhibit autosynergy.^29,30^ Thus, due to the auto-synergistic effect, compound 5f and 5f′ showed better oxidative stability than BHT.

Organosulfurs are one of the important additives in the lubricant compositions because of their antioxidant, antiwear and extreme pressure properties. And these have been usually utilized with the hindered phenol or aromatic amine to get better synergistic effect.^1^ Therefore, presence of two sulfur atoms and one hindered phenol in structure 5f′ may exhibit better auto-synergistic effect than the compound 5f. Thus, thiosemicarbazide containing thiolated BHT (5f′) is better oxidative stable than the commercial antioxidant BHT and semicarbazide 5f.

## Conclusions

In conclusion, semicarbazides (5a–h) and thiosemicarbazides (5a′–h′) bearing thiolated BHT were synthesized with high product yields as a potential multipotent antioxidant. All the synthesized compounds were carried out for DPPH assay for the evaluation of antioxidant properties. Better antioxidant properties were found for all the synthesized compounds than commercial antioxidant BHT. It was observed that electron donating substituents in semicarbazides enhanced the free radical scavenging properties than electro withdrawing substituents. Though, poor antioxidant property was obtained for semicarbazides containing electro donating substituent methoxy group (5d) due to formation of intramolecular hydrogen bond. However, thiosemicarbazides showed better free radical scavenging properties than semicarbazides series. It was anticipated that better antioxidant properties of compounds (5a′–h′) were found due to the thioamide–thioimidic acid tautomerism. In addition, thioamide of thiosemicarbazides can easily donate proton to reduce DPPH radical due to the lower electronegativity of sulfur (2.58) than oxygen (3.44). Again, based on the DPPH result and solubility in TMPTO, compound 5f′ and 5f were carried out isothermal DSC at two different isothermal temperatures for the evaluation of oxidative stability in comparison commercial antioxidant BHT. Due to strong auto-synergism between two secondary antioxidant functions (thioether and thioamide) and one primary antioxidant function (BHT moiety), compound 5f′ exhibited promising oxidative stability of synthetic ester-based lubricant oil. Therefore, it can also be concluded that there was a positive correlation between DPPH assay and DSC test. The further investigation on structure–thermodynamic–antioxidant relationships employ double H^+^/e^−^ process using density functional theory calculations and quantitative structure–activity relationship (QSAR) modelling are required to identify the specific substituents effect and kinetic mechanisms. Hence, the promising results are expected to get worldwide acceptance on synthetic lubricant oil.

## Author contributions

Data curation, Syabilah Sazeli; formal analysis, Syabilah Sazeli; funding acquisition, Wageeh A. Yehye and NWM Zulkifli; investigation, Syabilah Sazeli, Amit R. Nath and NWM Zulkifli; methodology, Syabilah Sazeli and Wageeh A. Yehye; project administration, Syabilah Sazeli; supervision, Wageeh A. Yehye and Lee Hwei Voon; writing – original draft, Syabilah Sazeli and Amit R. Nath; writing – review & editing, Wageeh A. Yehye, NWM Zulkifli, Amit R. Nath and Mohd Hafiz Ahmad.

## Conflicts of interest

There are no conflicts to declare.

## Supplementary Material

RA-011-D0RA10626G-s001
