# Supplementary material for: Semicarbazide and thiosemicarbazide containing butylated hydroxytoluene moiety: new potential antioxidant additives for synthetic lubricating oil
Source: RSC Adv. 2021 Feb 10;11(13):7138–45. doi: 10.1039/d0ra10626g (PMC8691112; doi:10.1039/d0ra10626g)
Supplement: RA-011-D0RA10626G-s001 [file RA-011-D0RA10626G-s001.pdf]

Electronic Supplementary Material (ESI) for RSC Advances.
This journal is © The Royal Society of Chemistry 2021

ELECTRONIC SUPPLEMENTARY INFORMATION

Semicarbazide and Thiosemicarbazide Containing Butylated Hydroxytoluene Moiety: New Potential Antioxidant Additives for Synthetic Lubricating Oil.

Syabilah Sazeli, [a] Amit R. Nath, [a] Mohd Hafiz Ahmad, [a] NWM Zulkifli, [b] Mohd Rafie Johan,[a] Wageeh A. Yehye,[a] and Lee Hwei Voon *[a].

[a] Nanotechnology & Catalysis Research Centre (NANOCAT), Institute for Advanced Studies, Level 3 Block A, Universiti Malaya, Kuala Lumpur 50603, Malaysia

[b] Department of Mechanical Engineering, Universiti Malaya, 50603 Kuala Lumpur, Malaysia

Email: leehweivoon@um.edu.my

*For correspondence

General reagents and instruments.

All materials and solvents were purchased in analytical grade from commercial suppliers and used without further purification. Toluene was dried over molecular sieves 4A (Sigma-Aldrich) prior to use. BHT and DPPH was purchased from Sigma-Aldrich. Merck TLC aluminium sheets (Silica gel 60 F254) were used for thin layer chromatography. All synthesized target compounds were characterized using FTIR, NMR, LCMS, and melting point instrument. An infrared spectrum was recorded on a Perkin Elmer, FTIR-Spectrum 400 spectrometer at wave number from 4000-400 cm^{-1}. 1D and 2D analysis of nuclear magnetic resonance (NMR) spectra were obtained on Bruker Advance III 600MHz. Spectra are reported in units of ppm on the scale, relative to chloroform and DMSO. The coupling constants are given in Hz. HR-mass spectra (ESI) were obtained with 6550 i-Funnel Q-TOF LC-MS. Melting points were determined with Mel-Temp II melting point apparatus.

Synthesis of starting material

The preparation of the starting material is outlined in Scheme 3.1.

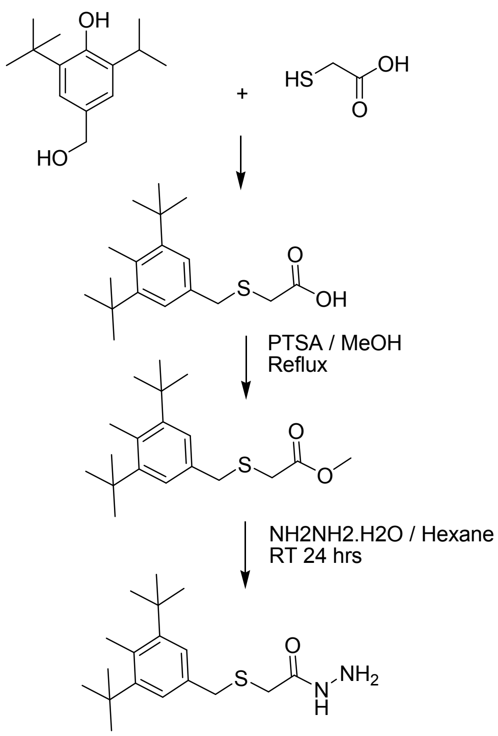

Scheme 3.1: Synthetic scheme for the preparation of 3.0.

3.1.2.1 *S-(3,5-di-tert-butyl-4-hydroxybenzyl) thioglycolic acid.*

Thioglycolic acid (4.8 g, 52 mmol) was added to a stirred solution of 3,5-di-tert-butyl-4-hydroxybenzyl alcohol (6 g, 25 mmol) in toluene (52 ml). PTSA (0.02 g) was added, and the solution was refluxed for 6 hours (Dean-Stark apparatus). The mixture was cooled, filtered and washed with distilled water to remove PTSA and unreacted thioglycollic acid, dried over anhydrous magnesium sulphate. The crude product, obtained after concentration under reduced pressure was purified by recrystallization from n-hexane to give 7.21 g of white solid (93%). Mp 97-99 ˚C.

3.1.2.2 *Methyl-S-(3,5-di-tert-butyl-4-hydroxybenzyl)thioglycolate.*

S-(3,5-di-tert-butyl-4-hydroxybenzyl)thioglycolic acid (6 g, 19.3 mmol), anhydrous methanol (200 ml) and p-toluenesulfonic acid (0.06 g) were refluxed for 24 hours. After cooling to RT, 10 ml of NaHCO3 (3%) was added, the solution was extracted by ethylacetate, filtered, dried over anhydrous sodium sulphate. The crude product, obtained after concentration under reduced pressure, was purified by column chromatography (hexane/ethyl acetate, 9 : 1) to give 6.01 g of brown oil (96 %). Bp 260-262˚C. IR (Nujol), cm^{-1}: 3647 (free OH), 1738 (C=O), 1434 (C-H methylene bending), 1030 (C-O stretching). ^{1}H NMR ($CDCl_3$, 400 MHz), δ, ppm : 1.43 (s, 18H, 2 × t-Bu), 3.12 (s, 2H, H-8), 3.72 (s, 3H, -OCH3), 3.76 (s, 2H, H-7), 5.19 (s, 1H, OH), 7.11 (s, 2H, H-3 and H-5). ^{13}C NMR ($CDCl_3$, 100 MHz), δ, ppm : 30.34 (6C, 2 × $–C(CH_3)_3$), 32.55 (1C, C-8), 34.39 (2C, 2 × $–C(CH_3)_3$), 36.93 (1C, C-7), 52.44 (1C, OCH_3), 125.96 (2C, C-3, C-5), 127.48 (1C, C-4), 136.02 (2C, C-2, C-6), 153.10 (1C, C-1), 171.13 (1C, C-9). HREIMS m/z 324.1763 $[M]^+$ (calcd for $C_{18}H_{28}O_3N_2S$ 324.1759).

3.1.2.3 *S-(3,5-di-tert-butyl-4-hydroxybenzyl)thioglycolic acid hydrazide, 3.0*

Methyl-S-(3,5-di-tert-butyl-4-hydroxybenzyl)thioglycolate (5 g, 15.40 mmol) was stirred with hydrazine hydrate 85% (2 ml) for 15 minutes. Hexane (150 ml) was added after 1 hour, followed by 6 ml of hydrazine hydrate and stirred at RT for 24 hours. The crude product was filtered washed with distilled water several times to remove unreacted hydrazine, dried, and purified by recrystallization from n-hexane to give white solid 4.34 g (87%). Mp 108-110˚C. IR (KBr pellet), cm^{-1}: 3634 (free OH), 3275-3324 (-NH-NH2), 2872-2962 (C-H of t-Bu), 1637 (C=O), 1433 (CH methylene bending). ^{1}H NMR (DMSO-d_6, 400 MHz), δ, ppm: 1.31 (s,18H, 2 × t-Bu), 2.95 (s, 2H, H-8), 3.67 (s, 2H, H-7), 4.21 (bs, 2H, NH2), 6.85 (s, 1H, OH), 6.99 (s, 2H, H-3, H-5),

9.10 (s, 1H, NH). ^{13}C NMR (DMSO-d6, 100 MHz), δ, ppm : 30.86 (6C, 2 × –C(CH3)3), 32.92 (1C, C-8), 34.99 (2C, 2 × –C(CH3)3), 36.79 1C, C-7), 125.73 (2C, C-3, C-5), 128.88 (1C, C-4), 139.70 (2C, C-2, C-6), 153.09 (1C, C-1), 169.00 (1C, C-9). HREIMS m/z 324.1881 [M]+ (calcd for $C_{17}H_{28}O_2N_2S$ 324.1872).

3.1.3 General procedure for the synthesis of semicarbazides and thiosemicarbazides (5a – 5h’)

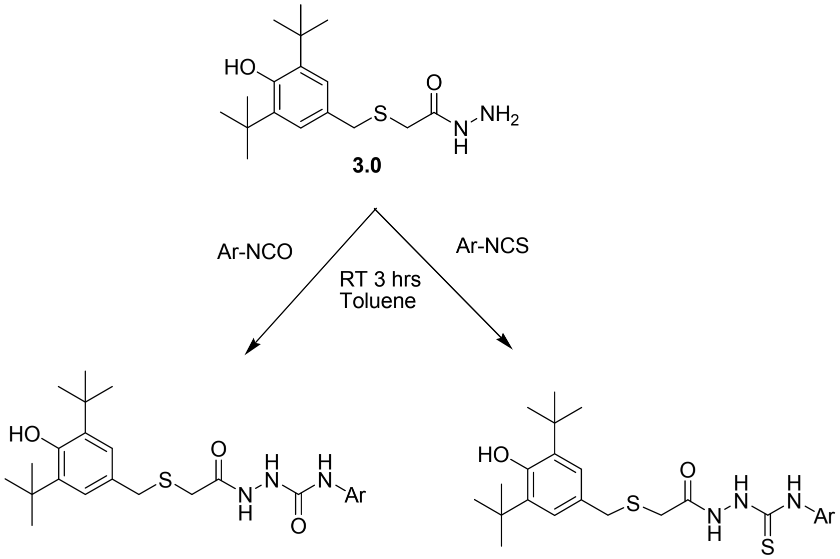

Compound	**R**
**5a, 5a’**	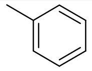
**5b, 5b’**	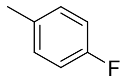
**5c, 5c’**	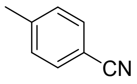
**5d, 5d’**	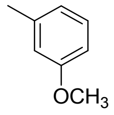
**5e, 5e’**	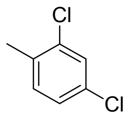
**5f, 5f’**	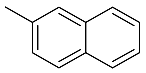
**5g, 5g’**	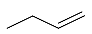
**5h, 5h’**	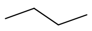

Scheme 3.2: Synthetic scheme for the preparation of 5a – 5h'.

The preparation of the target compounds is outlined in Scheme 3.2.

General procedure: To a solution of S-(3,5-di-tert-butyl-4-hydroxybenzyl)thioglycolic acid hydrazide (**3.0**; 0.3241 g, 1 mmol), dry toluene (5mL) was added isocyanate (1 mmol), and the reaction mixture was stirred at room temperature for 2 hours until the reaction completed (monitored by thin-layer chromatography). The white precipitate was collected by filtration, washed with water and boiled hexane, and dried at 50°C. The same procedure is applied to respective isothiocyanate group of reactants.

3.1.3.1 *2-(2-(3,5-di-tert-Butyl-4-hydroxybenzylthio)acetyl)-N-phenyl hydrazinecarboamide,* 5a

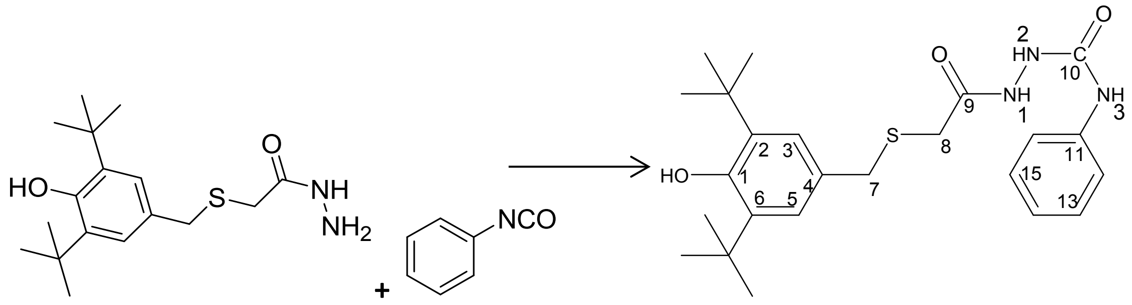

2-(2-(3,5-di-tert-Butyl-4-hydroxybenzylthio)acetyl)-N-phenyl hydrazinecarboamide was prepared from phenyl isocyanate (0.1191 g, 1.0 mmol), recrystallized from toluene to give 0.42 g of white solid (95 %). Mp 151°C. FTIR (ATR): 3619, 3252, 2960, 1605 cm^{-1}; ^{1}H NMR (600MHz, DMSO-d_6): δ = 9.83 (s, 1H), 8.72 (s, 1H), 8.13 (s, 1H), 7.46 (d, J = 6Hz, 2H), 7.26 (t, J = 6Hz, 2H), 7.08 (s,2H), 6.96 (t, J = 6Hz, 1H), 6.89 (s, 1H), 3.79 (s, 2H), 3.13 (s, 2H), 1.38 (s, 18H); ^{13}C NMR (150 MHz, DMSO-d_6): δ = 169.49, 155.68, 153.32, 140.06, 139.63, 129.12, 128.92, 125.73,

122.38, 118.90, 36.56, 34.95, 32.92, 30.83; HRMS (Q-TOF): m/z $[M+Na]^+$ = 466.2161, calcd for $C_{24}H_{33}NaN_3O_3S^+$ 466.2135.

3.1.3.2 *2-(2-(3,5-di-tert-Butyl-4-hydroxybenzylthio)acetyl)-N-(4-fluorophenyl) hydrazinecarboamide, 5b*

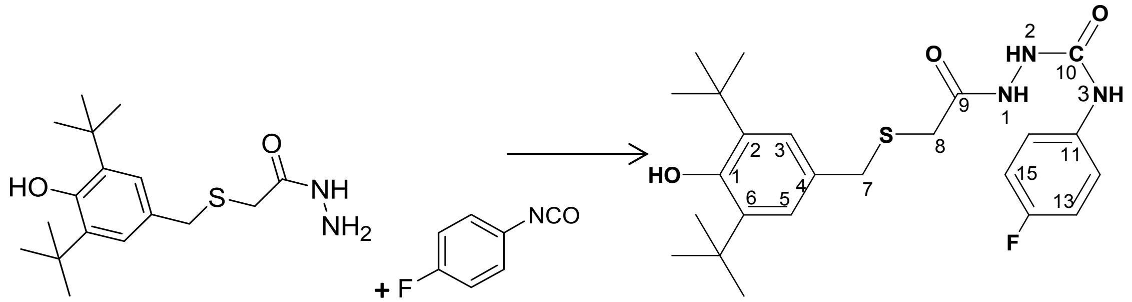

2-(2-(3,5-di-tert-Butyl-4-hydroxybenzylthio)acetyl)-N-(4-fluorophenyl) hydrazinecarboamide was prepared from 4-fluorophenyl isocyanate (0.1371 g, 1 mmol), recrystallized from toluene to give 0.44 g of white solid (96 %). Mp 162 °C. FTIR (ATR): 3634, 3292-3344, 2871-2955, 1656 cm^{-1}; ^1H NMR (600MHz, DMSO-d_6): δ = 9.81 (s,1H), 8.76 (s, 1H), 8.15 (s, 1H), 7.47 (m, 2H), 7.10 (m, 2H), 7.07 (s, 2H), 6.89 (s, 1H), 3.78 (s, 2H), 3.12 (s, 2H), 1.37 (s, 18H); ^{13}C NMR (150 MHz, DMSO-d_6): δ = 169.49, 158.64, 157.06, 153.31, 139.64, 136.40, 128.92, 120.70, 115.66, 115.52, 36.55, 34.94, 32.93, 30.82; HRMS (Q-TOF): m/z $[M+H]^+$ = 462.2225, calcd for $C_{24}H_{33}O_3N_3SF^+$ 462.2221.

3.1.3.3 *2-(2-(3,5-di-tert-Butyl-4-hydroxybenzylthio)acetyl)-N-4-(cyanophenyl) hydrazinecarboamide, 5c*

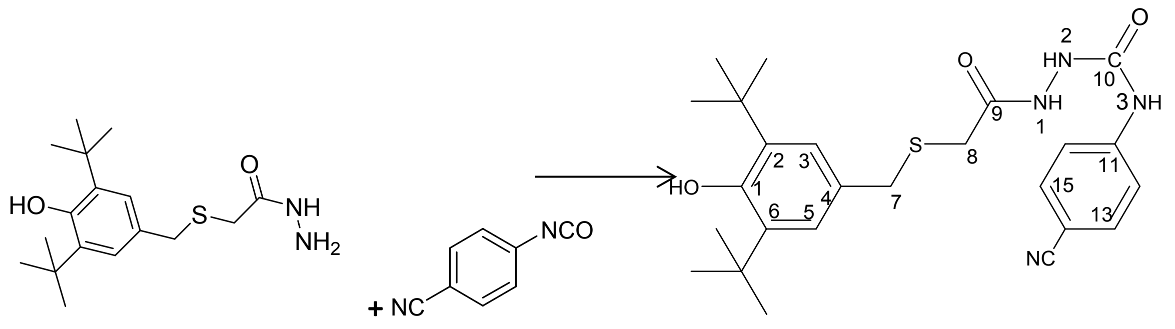

2-(2-(3,5-di-tert-Butyl-4-hydroxybenzylthio)acetyl)-N-(4-cyanophenyl) hydrazinecarboamide was prepared from 4-cyanophenyl isocyanate (0.1441 g, 1 mmol), recrystallized from toluene to give 0.42 g of white solid (91 %). Mp 141-148 ^{o}C. FTIR (ATR): 3589-3649, 3337, 2956-3025, 2234, 1601 cm^{-1} ; ^{1}H NMR (600MHz, DMSO-d$_6$): $\boldsymbol{\delta}$ = 9.89 (s, 1H), 9.27 (b, 1H), 8.43 (s, 1H), 7.71 (d, *J*=6 Hz, 2H), 7.65 (d, *J*=6, 2H), 6.88 (s, 1H), 3.78 (s, 2H), 3.14 (s, 2H), 1.38 (s, 18H); ^{13}C NMR (150 MHz, DMSO-d$_6$): $\boldsymbol{\delta}$ = 169.51, 155.24, 153.33, 144.66, 139.64, 133.64, 128.89, 125.73, 119.75, 118.74, 103.86, 36.53, 34.95, 32.87, 30.82; HRMS (Q-TOF): m/z [M+H]$^+$ = 469.2265, calcd for $C_{25}H_{33}O_3N_4S^+$ 469.2268.

3.1.3.4 ***2-(2-(3,5-di-tert-Butyl-4-hydroxybenzylthio)acetyl)-N-(3-methoxyphenyl) hydrazinecarboamide, 5d***

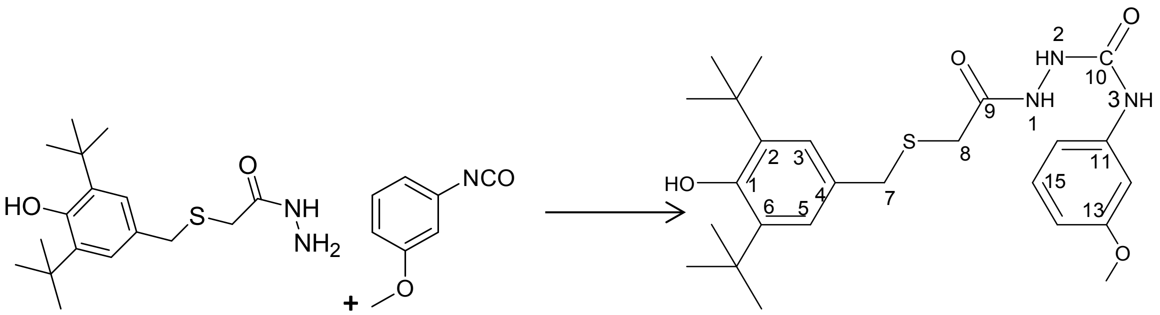

2-(2-(3,5-di-tert-Butyl-4-hydroxybenzylthio)acetyl)-N-(3-methoxyphenyl) hydrazinecarboamide was prepared from 3-methoxyphenyl isocyanate (0.1492 g, 1 mmol), recrystallized from toluene to give 0.44 g of white solid (93 %). Mp 136°C. FTIR (ATR): 3624, 3288, 2961, 1683 cm^{-1}; ^{1}H NMR (600MHz, DMSO-d_6): δ = 9.82 (b, 1H), 8.72 (b, 1H), 8.12 (b, 1H), 7.16 (m, 1H), 7.08 (s, 2H), 6.98 (d, *J*=6 Hz, 1H), 6.88 (s, 1H), 6.54 (dd, *J*=6 *J*=6 Hz, 1H), 3.79 (s, 2H), 3.71 (s, 3H), 3.13 (s, 2H), 1.38 (s, 18H); ^{13}C NMR (150 MHz, DMSO-d_6): δ = 169.49, 158.64, 157.06, 153.31, 139.64, 136.40, 128.92, 120.70, 115.59, 36.55, 34.94, 32.93, 30.82; HRMS (Q-TOF): m/z $[M+H]^+$ = 475.2470, calcd for $C_{25}H_{36}O_4N_3S^+$ 475.2442.

3.1.3.5 ***2-(2-(3,5-di-tert-Butyl-4-hydroxybenzylthio)acetyl)-N-(2,4-dichlorophenyl) hydrazinecarboamide, 5e***

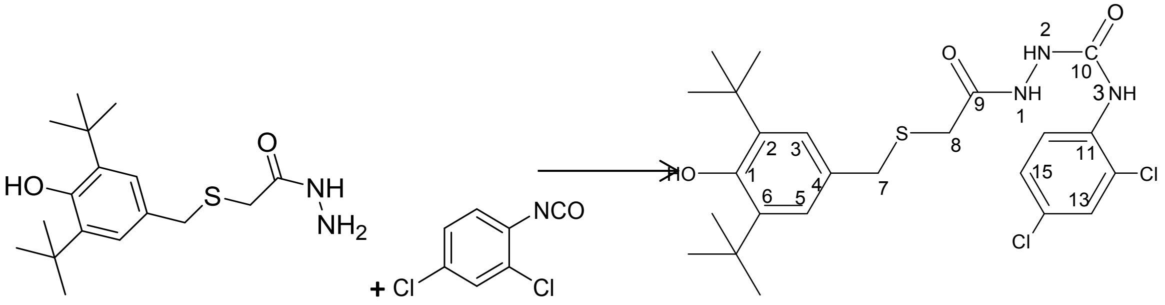

2-(2-(3,5-di-tert-Butyl-4-hydroxybenzylthio)acetyl)-N-(2,4-dichlorophenyl) hydrazinecarboamide was prepared from 2,4-dichlorophenyl isocyanate (0.1880 g, 1 mmol), recrystallized from toluene to give 0.46 g of white solid (90 %). Mp 106°C. FTIR (ATR): 3644, 3184-3332, 2862-2956, 1591 cm^{-1}; ^{1}H NMR (600MHz, DMSO-d_6): δ = 10.00 (s, 1H), 8.91 (s, 1H), 8.33 (b, 1H), 8.11 (d, ^{3}J=6 Hz 1H), 7.62 (d, ^{4}J=0 Hz 1H), 7.37 (dd, ^{3}J=6, ^{4}J=0 Hz 1H), 7.07 (s, 2H), 6.89 (s, 1H), 3.78 (s, 2H), 3.13 (s, 2H), 1.37 (s, 18H); ^{13}C NMR (150 MHz, DMSO-d_6): δ = 169.40, 155.03, 153.33, 139.63, 135.50, 129.06, 128.84, 128.14, 126.80, 125.74, 123.28, 122.57, 36.52, 34.94, 32.65, 30.81; HRMS (Q-TOF): m/z $[M+Na]^+$ = 510.1373, calcd for $C_{22}H_{32}O_3NaN_3SCl_2^+$ 510.1336.

3.1.3.6 *2-(2-(3,5-di-tert-Butyl-4-hydroxybenzylthio)acetyl)-N-(1-naphthyl) hydrazinecarboamide, 5f*

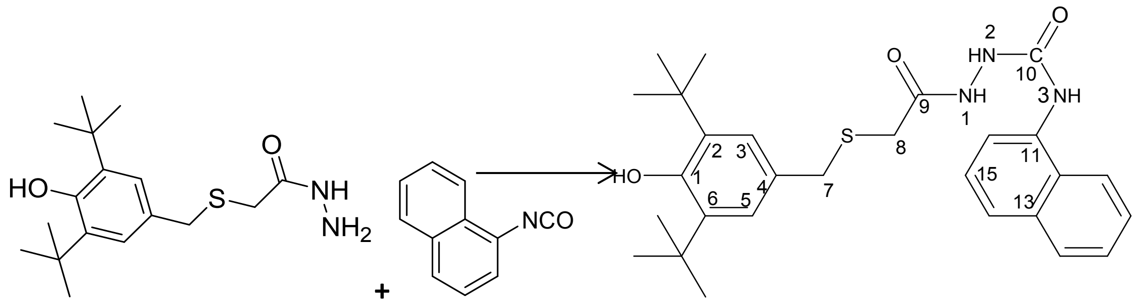

2-(2-(3,5-di-tert-Butyl-4-hydroxybenzylthio)acetyl)-N-(1-naphthyl) hydrazinecarboamide was prepared from 1-naphthyl isocyanate (0.1692 g, 1 mmol), recrystallized from toluene to give 0.44 g of white solid (89 %). Mp 123 ^{o}C. FTIR (ATR): 3639, 3200-3302, 2873-2961, 1610 cm^{-1} ^{1}H NMR (600MHz, DMSO-d$_6$): $\boldsymbol{\delta}$ = 9.96 (s, 1H), 8.79 (s, 1H), 8.43 (s, 1H), 8.06 (d, ^{4}J=12 Hz, 1H), 7.92 (m, 1H), 7.80 (b, 1H), 7.67 (d, ^{3}J=6 Hz, 1H), 7.53 (m, 2H), 7.46 (t, ^{3}J=6, ^{3}J=6 Hz, 1H), 7.08 (s, 2H), 6.90 (s, 1H), 3.80 (s, 2H), 3.16 (s, 2H), 1.36 (s, 18H); ^{13}C NMR (150 MHz, DMSO-d$_6$): $\boldsymbol{\delta}$ = 169.60, 156.42, 153.32, 139.69, 134.58, 134.19, 128.90, 128.74, 126.41, 126.21, 126.16, 125.74, 124.25, 122.38, 119.42, 36.59, 34.92, 32.91, 30.81; HRMS (Q-TOF): m/z $[M+H]^{+}$ = 494.2473, calcd for $C_{28}H_{36}O_3N_3S^{+}$ 494.2472.

3.1.3.7 *2-(2-(3,5-di-tert-Butyl-4-hydroxybenzylthio)acetyl)-N-(Allyl) hydrazinecarboamide, 5g*

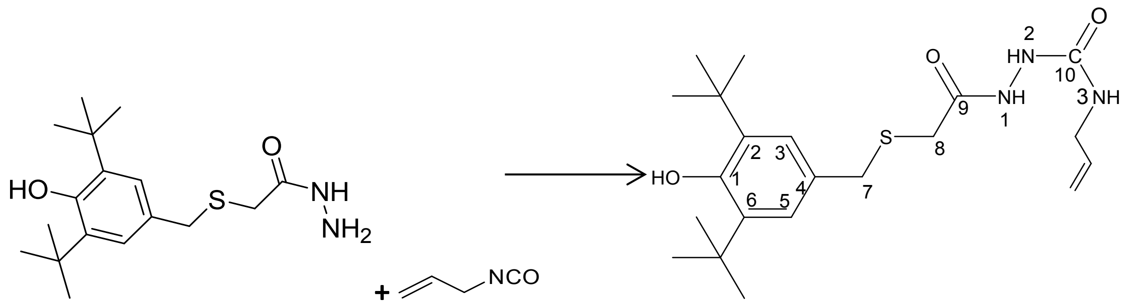

2-(2-(3,5-di-tert-Butyl-4-hydroxybenzylthio)acetyl)-N-(allyl) hydrazinecarboamide was prepared from Allyl isocyanate (0.0831 g, 1 mmol), recrystallized from toluene to give 0.37 g of white solid (90 %). Mp 129 ^{o}C. FTIR (ATR): 3619, 3436.3-3515, 3139, 2867-2956, 1675 cm^{-1}; ^{1}H NMR (600MHz, DMSO-d_6): $\boldsymbol{\delta}$ = 9.63 (s, 1H), 7.86 (s, 1H), 7.06 (s, 2H), 6.86 (s, 1H), 6.47 (t, ^{3}J=6, ^{3}J=6 Hz, 1H), 5.80 (m, 1H), 5.15 (dd, ^{3}J=18, ^{2}J=0 Hz, 1H), 5.03 (dd, ^{3}J=12, ^{2}J=0 Hz, 1H), 3.75 (s, 2H), 3.66 (t, ^{3}J=6, ^{3}J=6 Hz, 2H), 3.08 (s, 2H), 1.38 (s, 18H); ^{13}C NMR (150 MHz, DMSO-d$_6$): $\boldsymbol{\delta}$ = 169.39, 158.27, 153.30, 139.62, 136.75, 128.96, 125.68, 114.94, 41.94, 36.62, 34.94, 32.99, 30.84; HRMS (Q-TOF): m/z [M+Na]$^{+}$ = 430.2154, calcd for $C_{21}H_{33}O_3NaN_3S^+$ 430.2135.

3.1.3.8 *2-(2-(3,5-di-tert-Butyl-4-hydroxybenzylthio)acetyl)-N-Butyl hydrazine carboamide, 5h*

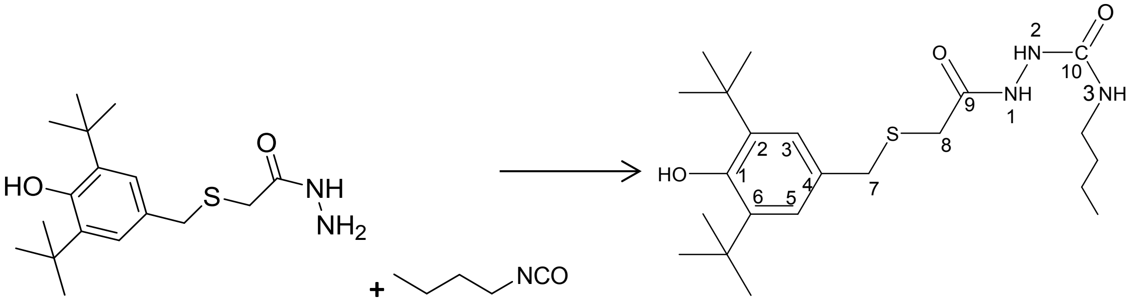

2-(2-(3,5-di-tert-Butyl-4-hydroxybenzylthio)acetyl)-N-(butyl) hydrazinecarboamide was prepared from n-Butyl isocyanate (0.0991 g, 1 mmol), recrystallized from toluene to give 0.37 g of white solid (87 %). Mp 84 ^{o}C. FTIR (ATR): 3375 , 3132, 2849-2917, 1635 cm^{-}1; ^{1}H NMR (600MHz, DMSO-d_6): $\boldsymbol{\delta}$ = 9.60 (s, 1H), 7.75 (s, 1H), 7.06 (s, 2H), 6.87 (s, 1H), 6.26 (t, ^{3}J=6, ^{3}J=6 Hz, 1H), 3.75 (s, 2H), 3.07 (s, 2H), 3.01 (m, 2H), 1.37 (s, 18H), 1.26 (m, 2H), 0.87 (t, ^{3}J=6, ^{3}J=6 Hz, 3H); ^{13}C NMR (150 MHz, DMSO-d$_6$): $\boldsymbol{\delta}$ = 169.35), 158.41, 153.30, 139.61, 128.93, 125.69, 39.30, 36.59, 34.93, 32.92, 32.42, 30.82, 19.88, 14.17; HRMS (Q-TOF): m/z [M+H]$^{+}$ = 424.2637, calcd for $C_{22}H_{38}O_3N_3S^+$ 424.2628.

3.1.3.9 *2-(2-(3,5-di-tert-Butyl-4-hydroxybenzylthio)acetyl)-N-phenyl hydrazinecarbothioamide, 5a'*

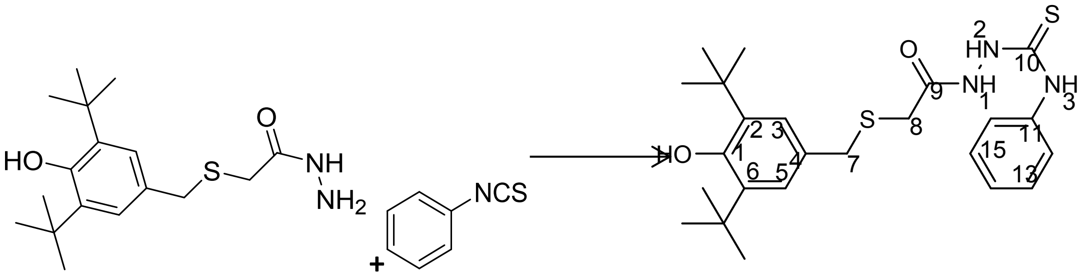

2-(2-(3,5-di-tert-Butyl-4-hydroxybenzylthio)acetyl)-N-phenyl hydrazinecarbothioamide was prepared from phenyl isothiocyanate (0.1352 g, 1 mmol), recrystallized from toluene to give 0.43 g of white solid (94 %). Mp 128°C. FTIR (ATR): 3634, 3144, 2956, 1670, 1225 cm^{-1}; ^{1}H NMR (600MHz, DMSO-d$_6$): $\boldsymbol{\delta}$ = 10.10 (s, 1H), 9.67 (s, 1H), 9.60 (b, 1H), 7.45 (s, 2H), 7.34 (t, ^{3}J=6, ^{3}J=6 Hz, 1H), 7.17 (t, ^{3}J=6, ^{3}J=6 Hz, 1H), 7.08 (s, 2H), 6.90 (s, 1H), 3.79 (s, 2H), 3.17 (s, 2H), 1.38 (s, 18H); ^{13}C NMR (150 MHz, DMSO-d_6): $\boldsymbol{\delta}$ = 181.39, 169.38, 153.34, 139.66, 139.55, 128.87, 128.59, 126.09, 125.70, 125.51, 36.67, 34.95, 33.35, 30.84; HRMS (Q-TOF): m/z [M+H]$^{+}$ = 460.2096, calcd for $C_{24}H_{34}O_3N_3S_2{}^{+}$ 460.2087.

3.1.3.10 ***2-(2-(3,5-di-tert-Butyl-4-hydroxybenzylthio)acetyl)-N-(4-fluorophenyl) hydrazinecarbothioamide, 5b’***

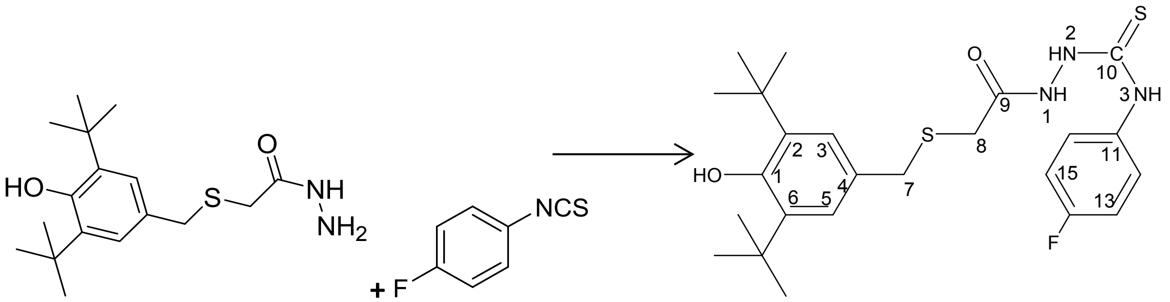

2-(2-(3,5-di-tert-Butyl-4-hydroxybenzylthio)acetyl)-N-(4-fluorophenyl) hydrazinecarbothioamide was prepared from 4-fluorophenyl isothiocyanate (0.1532 g, 1 mmol), recrystallized from toluene to give 0.45 g of white solid (95 %). Mp 111°C. FTIR (ATR): 3624, 3139-3283, 2956, 1670, 1220 cm^{-1}; ^{1}H NMR (600MHz, $CDCl_3$): $\boldsymbol{\delta}$ = 9.47 (b, 1H), 8.54 (b, 1H), 8.11 (s, 1H), 7.39 (m, 2H), 7.12 (s, 2H), 7.11 (d, *J*=6 Hz, 2H), 5.24 (s, 1H), 3.85 (s, 2H), 3.27 (s, 2H), 1.44 (s, 18H); ^{13}C NMR (150 MHz, $CDCl_3$): $\boldsymbol{\delta}$ = 166.22, 162.09, 160.46, 153.29, 136.47, 132.47, 127.07, 126.92, 125.86, 116.64, 116.49, 37.75, 34.35, 33.76, 30.23; HRMS (Q-TOF): m/z $[M+Na]^+$ = 500.1827, calcd for $C_{24}H_{33}O_2NaN_3S_2F^+$ 500.1812.

3.1.3.11 ***2-(2-(3,5-di-tert-Butyl-4-hydroxybenzylthio)acetyl)-N-(4-cyanophenyl) hydrazinecarbothioamide, 5c'***

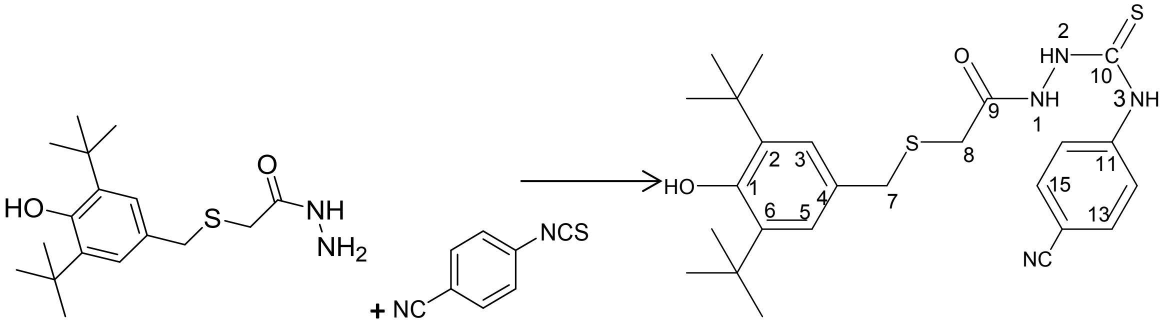

2-(2-(3,5-di-tert-Butyl-4-hydroxybenzylthio)acetyl)-N-(4-cyanophenyl) hydrazinecarbothioamide was prepared from 4-cyanophenyl isothiocyanate (0.1602 g, 1 mmol), recrystallized from toluene to give 0.43 g of white solid (90 %). Mp 154°C. FTIR (ATR): 3634, 3127-3205, 2956, 2229, 1600, 1240 cm^{-1}; ^{1}H NMR (600MHz, $CDCl_3$): $\boldsymbol{\delta}$ = 9.81 (b, 2H), 8.93 (b, 1H), 7.66 (d, *J*=6 Hz, 2H), 7.53 (d, *J*=6 Hz, 2H), 7.03 (s, 2H), 5.17 (s, 1H), 3.76 (s, 2H), 3.25 (s, 2H), 1.34 (s, 18H); ^{13}C NMR (150 MHz, $CDCl_3$): $\boldsymbol{\delta}$ = 179.79, 167.41, 153.42, 142.01, 136.63, 133.03, 126.59, 125.78, 122.55, 118.55, 107.53, 37.78, 34.36, 33.97, 30.22; HRMS (Q-TOF): m/z $[M+H]^+$ =485.2043, calcd for $C_{25}H_{33}O_2N_4S_2^+$ 485.2039.

3.1.3.12 ***2-(2-(3,5-di-tert-Butyl-4-hydroxybenzylthio)acetyl)-N-(3-methoxyphenyl) hydrazinecarbothioamide, 5d'***

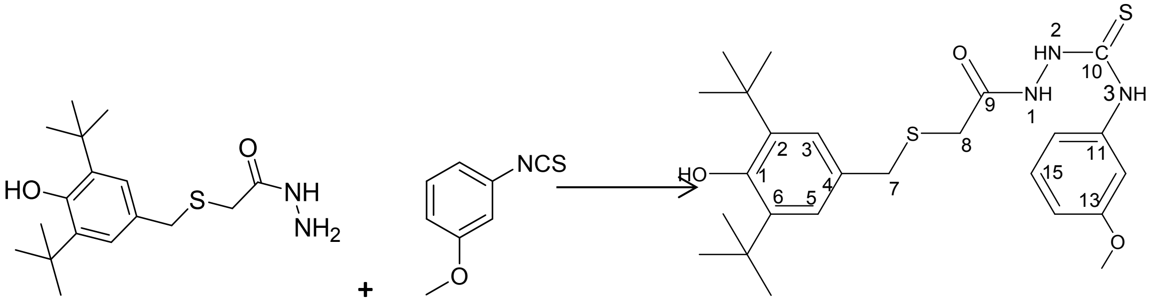

2-(2-(3,5-di-tert-Butyl-4-hydroxybenzylthio)acetyl)-N-(3-methoxyphenyl) hydrazinecarbothioamide was prepared from 3-methoxyphenyl isothiocyanate (0.1652 g, 1 mmol), recrystallized from toluene to give 0.46 g of white solid (94 %). Mp 111°C. FTIR (ATR): 3634, 3283-3332, 2834-2956, 1674, 1162 cm^{-1}; ^{1}H NMR (600MHz, DMSO-d_6): $\boldsymbol{\delta}$ = 10.09 (b, 1H), 9.68 (s, 1H), 9.54 (b, 1H), 7.24 (t, ^{3}J=6, ^{3}J=6 Hz, 1H), 7.17 (s, 1H), 7.08 (s, 2H), 7.04 (dd, ^{3}J=6, ^{4}J=6 Hz, 1H), 6.89 (s, 1H), 6.74 (d, ^{3}J=6 Hz, 1H), 3.79 (s, 2H), 3.74 (s, 1H), 3.17 (s, 2H), 1.38 (s, 18H); ^{13}C NMR (150 MHz, DMSO-d$_6$): $\boldsymbol{\delta}$ = 181.20, 169.37, 159.50, 153.34, 140.67, 139.66, 129.37, 128.86, 125.71, 110.93, 55.57, 36.68, 34.95, 33.33, 30.83; HRMS (Q-TOF): m/z [M+H]$^{+}$ = 491.2245, calcd for $C_{25}H_{36}O_3N_3S_2^+$ 491.2293.

3.1.3.13 ***2-(2-(3,5-di-tert-Butyl-4-hydroxybenzylthio)acetyl)-N-(2,4-dichlorophenyl) hydrazinecarbothioamide, 5e'***

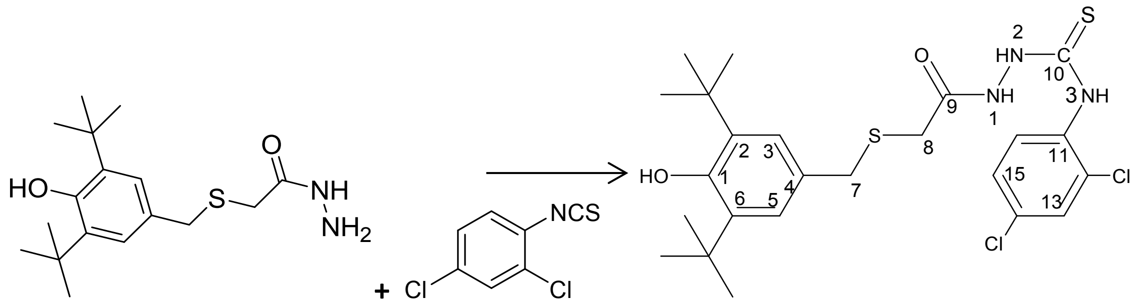

2-(2-(3,5-di-tert-Butyl-4-hydroxybenzylthio)acetyl)-N-(2,4-dichlorophenyl) hydrazinecarbothioamide was prepared from 2,4-dichlorophenyl isothiocyanate (0.2041 g, 1 mmol), recrystallized from toluene to give 0.51 g of white solid (96 %). Mp 131-136°C. FTIR (ATR): 3634, 3174-3278, 2965, 1651, 1275 cm^{-1}; ^{1}H NMR (600MHz, DMSO-d$_6$): $\boldsymbol{\delta}$ = 10.19 (b, 1H), 9.90 (b, 1H), 9.48 (b, 1H), 7.67 (s, 1H), 7.43 (d, ^{3}J=6 Hz, 2H), 7.07 (s, 2H), 6.89 (s, 1H), 3.78 (s, 2H), 3.16 (s, 2H), 1.37 (s, 18H); ^{13}C NMR (150 MHz, DMSO-d$_6$): $\boldsymbol{\delta}$ = 182.34, 169.41, 153.34, 139.66, 136.35, 132.86, 132.49, 131.94, 129.28, 128.85, 127.78, 125.69, 36.69, 34.94, 33.24, 30.83; HRMS (Q-TOF): m/z [M+H]$^+$ = 526.1147, calcd for $C_{24}H_{32}O_2N_3S_2Cl_2^+$ 526.1138.

3.1.3.14 ***2-(2-(3,5-di-tert-Butyl-4-hydroxybenzylthio)acetyl)-N-1-naphthyl hydrazine carbothioamide, 5f'***

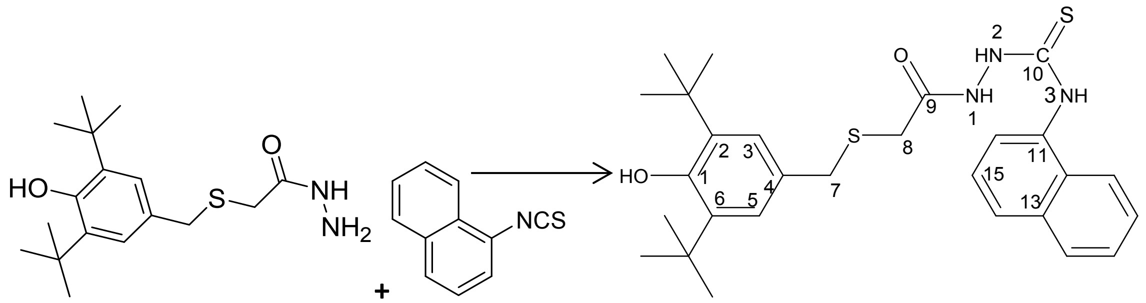

2-(2-(3,5-di-tert-Butyl-4-hydroxybenzylthio)acetyl)-N-(1-naphthyl) hydrazine carboamide was prepared from 1-naphthyl isothiocyanate (0.1852 g, 1 mmol), recrystallized from toluene to give 0.47 g of white solid (92 %). Mp 121 ^oC. FTIR (ATR): 3644, 3166-3258, 2956, 1698 cm^{-1}; ^1H NMR (600MHz, $CDCl_3$): $\boldsymbol{\delta}$ = 9.79 (b, 1H), 9.04 (b, 1H), 8.54 (s, 1H), 7.92 (m, 1H), 7.82 (m, 1H), 7.76 (d, ^3J=12 Hz, 1H), 7.58 (d, ^3J=12 Hz, 1H), 7.46 (m, 1H), 7.45 (s, 1H), 7.42 (m, 1H), 7.00 (s, 2H), 5.08 (s, 1H), 3.72 (s, 2H), 3.07 (s, 2H), 1.31 (s, 18H); ^{13}C NMR (150 MHz, $CDCl_3$): $\boldsymbol{\delta}$ = 179.16, 165.29, 153.24, 136.29, 134.51, 132.15, 129.58, 128.57; 128.53, 127.27, 126.87, 126.83, 125.87, 125.63, 124.92, 122.10, 37.62, 34.31, 33.69, 30.24; HRMS (Q-TOF): m/z $[M+H]^+$ = 510.2241, calcd for $C_{28}H_{36}O_2N_3S_2^+$ 510.2243.

3.1.3.15 ***2-(2-(3,5-di-tert-Butyl-4-hydroxybenzylthio)acetyl)-N-(Allyl) hydrazine carbothioamide, 5g'***

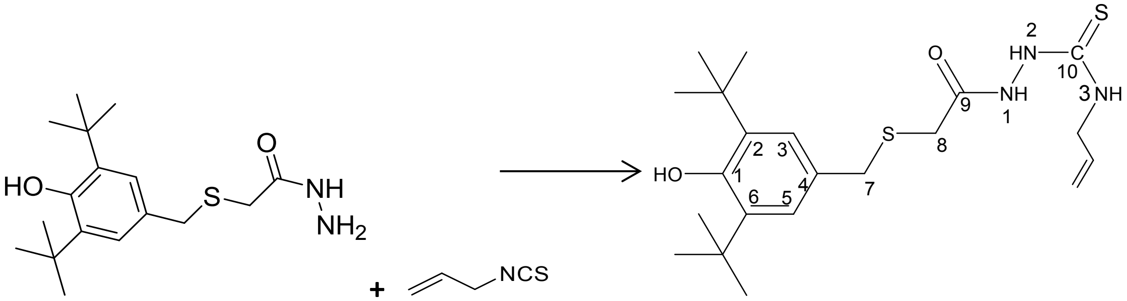

2-(2-(3,5-di-tert-Butyl-4-hydroxybenzylthio)acetyl)-N-(Allyl) hydrazinecarbothioamide was prepared from Allyl isothiocyanate (0.0992 g, 1 mmol), recrystallized from toluene to give 0.36 g of white solid (85 %). Mp 113-124 ^{o}C. FTIR (ATR): 3619, 3439-3512, 3141, 2868-2956, 1674, 1230 cm^{-1}; ^{1}H NMR (600MHz, DMSO-d_6): $\boldsymbol{\delta}$ = 9.88 (s, 1H), 9.32 (s, 1H), 8.05 (s, 1H), 7.07 (s, 2H), 6.88 (s, 1H), 5.82 (m, 1H), 5.13 (dd, ^{3}J=18, ^{2}J=6 Hz, 1H), 5.04 (dd, ^{3}J=12, ^{2}J=0 Hz, 1H), 4.10 (s, 2H), 3.75 (s, 2H), 3.11 (s, 2H), 1.38 (s, 18H); ^{13}C NMR (150 MHz, DMSO-d$_6$): $\boldsymbol{\delta}$ = 182.35, 169.39, 153.33, 139.64, 135.31, 128.87, 125.68, 115.76, 46.31, 36.66, 34.94, 33.27, 30.84; HRMS (Q-TOF): m/z [M+H]$^+$ = 424.2110, calcd for $C_{21}H_{34}O_2N_3S_2^+$ 424.2117.

3.1.3.16 *2-(2-(3,5-di-tert-Butyl-4-hydroxybenzylthio)acetyl)-N-(Butyl) hydrazine carbothioamide, 5h'*

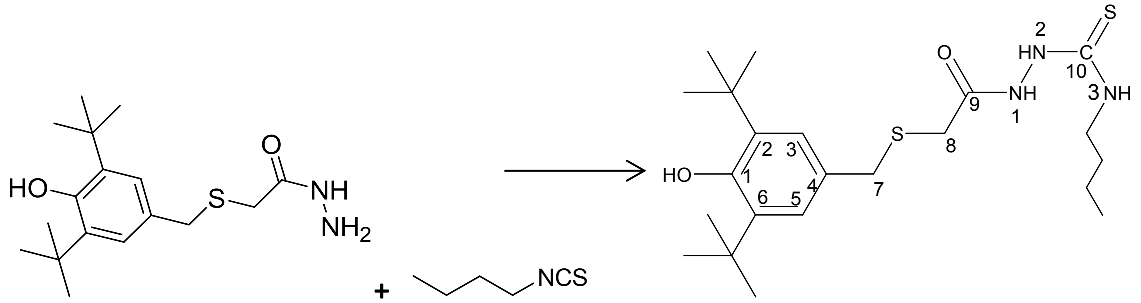

2-(2-(3,5-di-tert-Butyl-4-hydroxybenzylthio)acetyl)-N-(Butyl) hydrazinecarbothioamide was prepared from n-Butyl isothiocyanate (0.1152 g, 1 mmol), recrystallized from toluene to give 0.41 g of white solid (93 %). Mp 122-127°C. FTIR (ATR): 3619-3639, 3346, 3215-3273, 2868-2956, 1664, 1225 cm^{-1}; ^{1}H NMR (600MHz, DMSO-d_6): $\boldsymbol{\delta}$ = 9.83 (s, 1H), 9.20 (s, 1H), 7.82 (s, 1H), 7.06 (s, 2H), 6.89 (s, 1H), 3.75 (s, 2H), 3.42 (d, ^{3}J=6 Hz, 2H), 3.10 (s, 2H), 1.46 (m, 2H), 1.38 (s, 18H), 1.25 (m, 2H), 0.88 (t, ^{3}J=6, ^{3}J=6 Hz, 3H); ^{13}C NMR (150 MHz, DMSO-d$_6$): $\boldsymbol{\delta}$ = 192.18, 169.49, 158.64, 157.06, 153.31, 139.65, 128.86, 125.69, 43.80, 36.63, 34.94, 33.19, 31.32, 30.83, 19.88, 14.25; HRMS (Q-TOF): m/z [M+H]$^{+}$ = 440.2424, calcd for $C_{22}H_{38}O_2N_3S_2^{+}$ 440.2400.

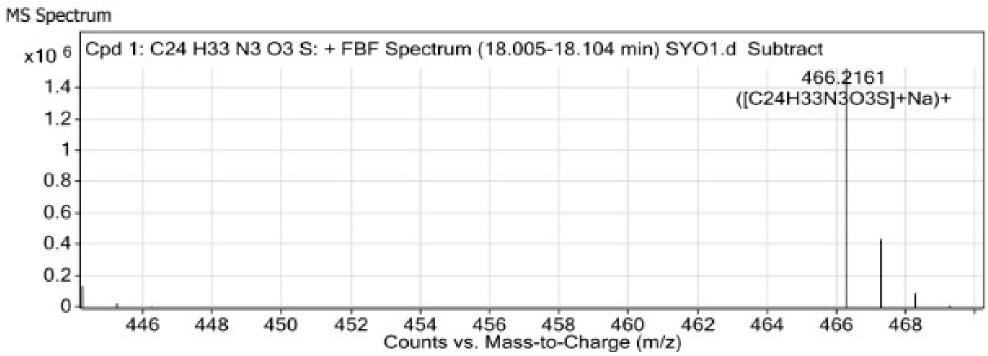

HRMS (ESI) of 5a

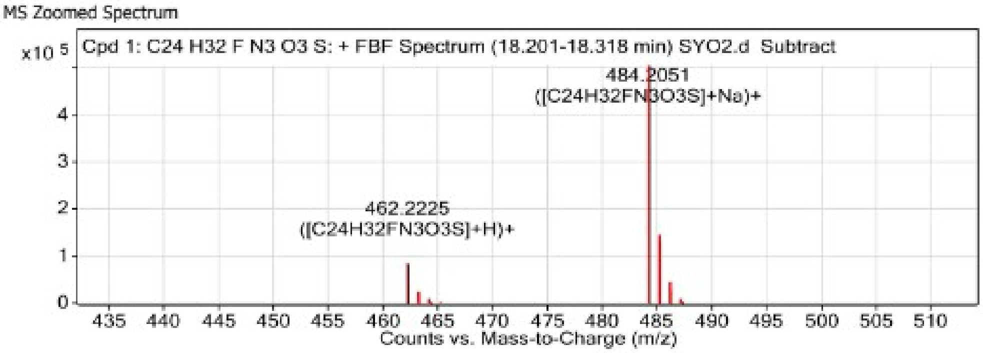

HRMS (ESI) of 5b

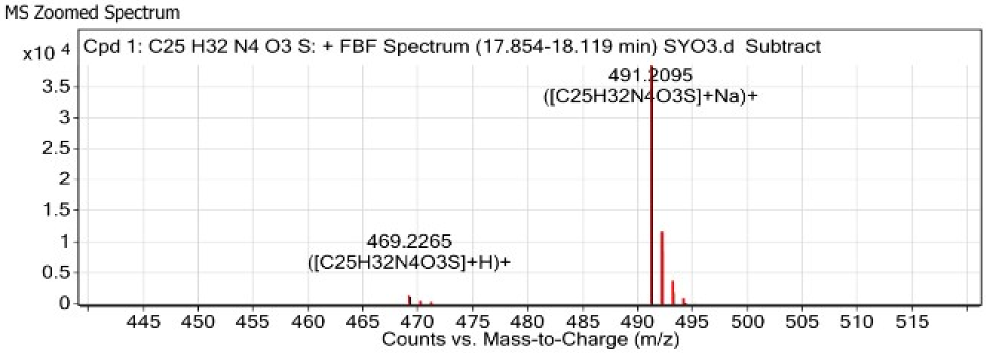

HRMS (ESI) of 5c

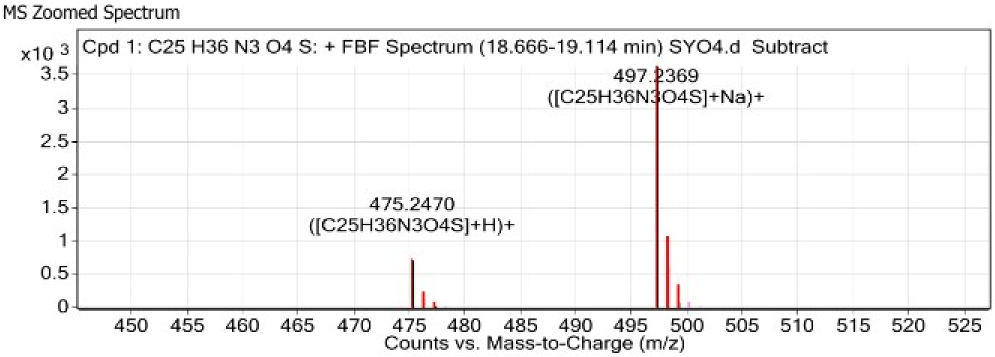

HRMS (ESI) of 5d

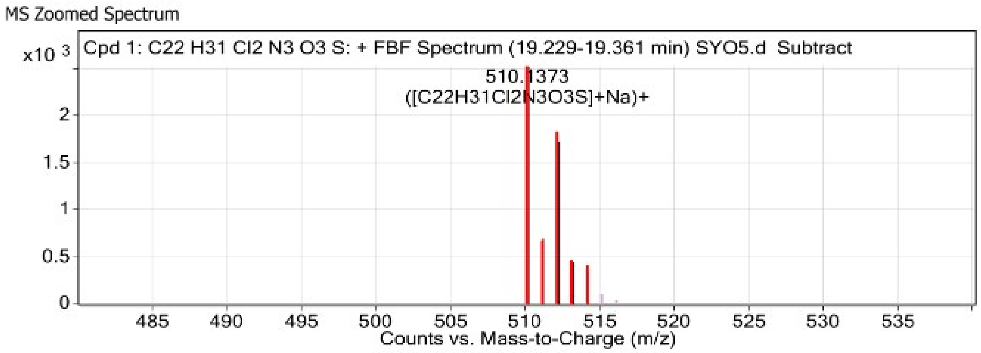

HRMS (ESI) of 5e

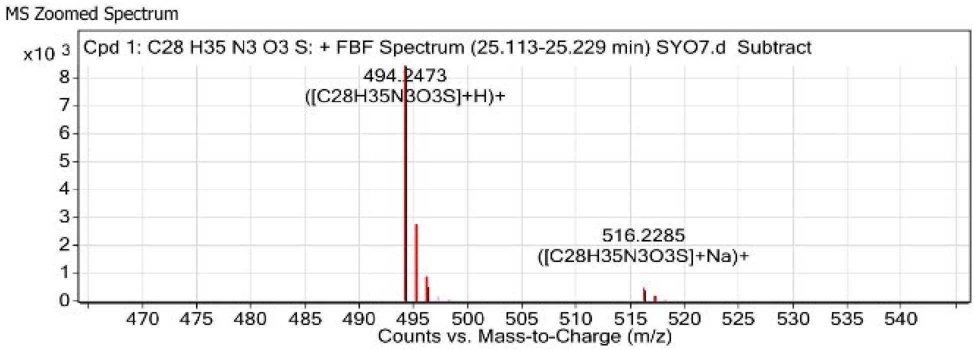

HRMS (ESI) of 5f

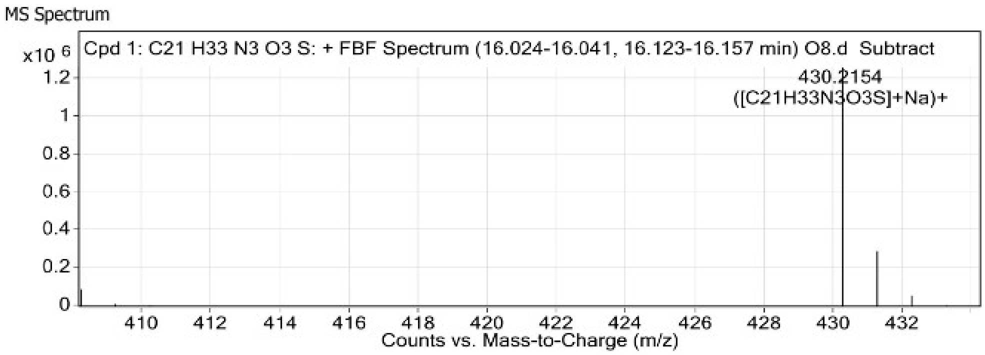

HRMS (ESI) of 5g

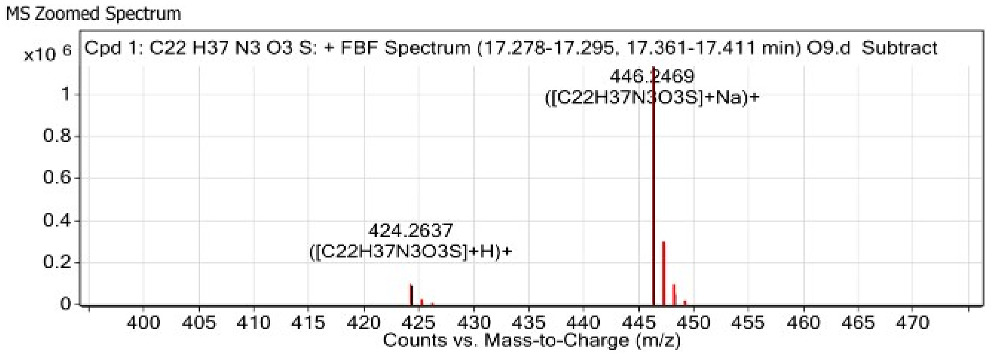

HRMS (ESI) of 5h

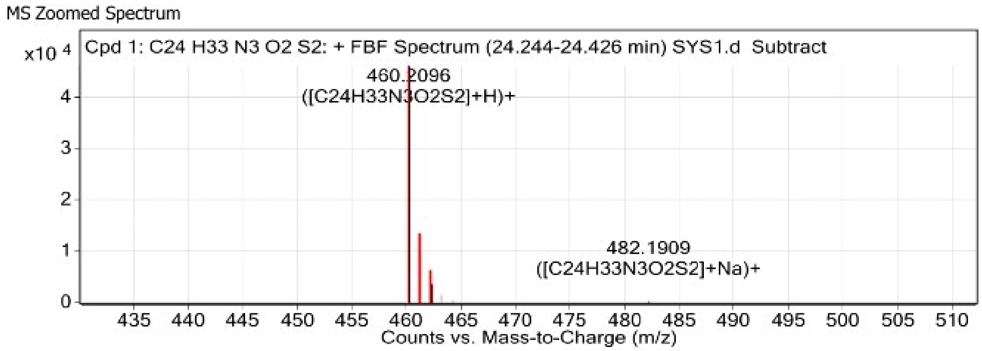

HRMS (ESI) of 5a'

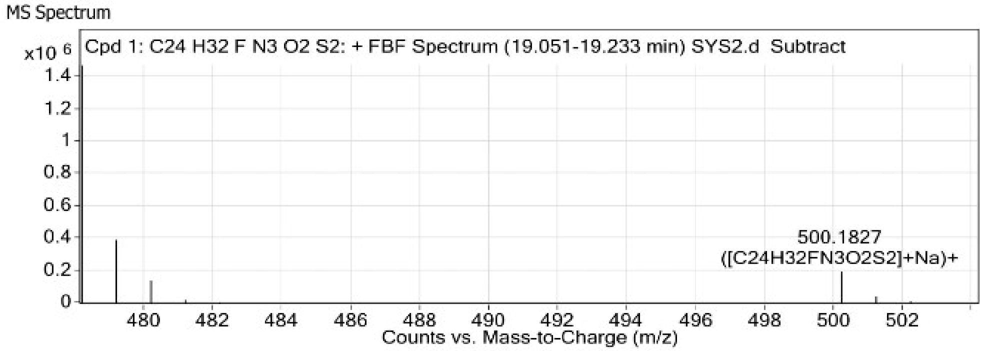

HRMS (ESI) of 5b'

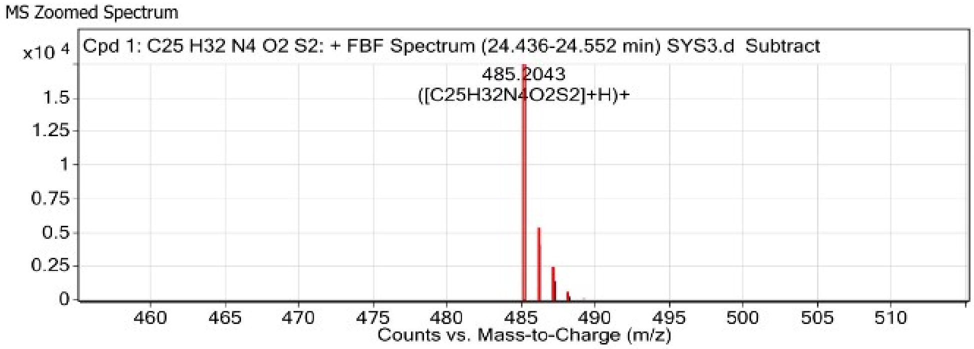

HRMS (ESI) of 5c'

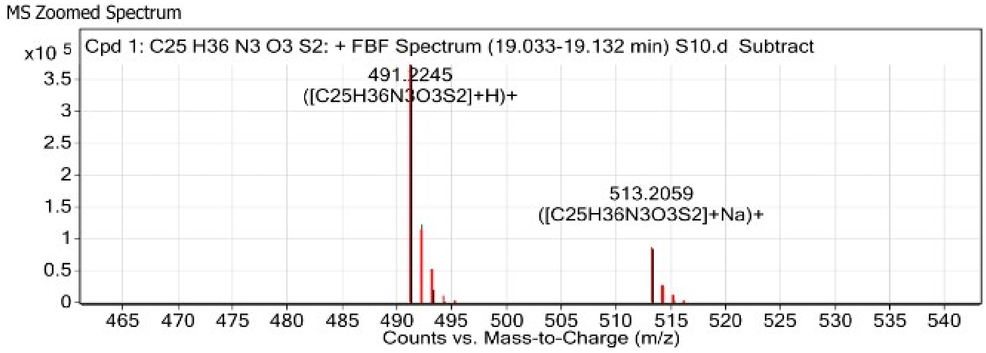

HRMS (ESI) of 5d'

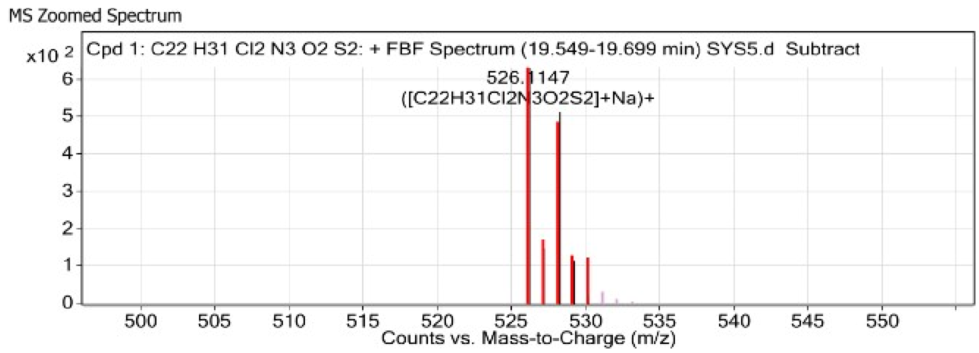

HRMS (ESI) of 5e'

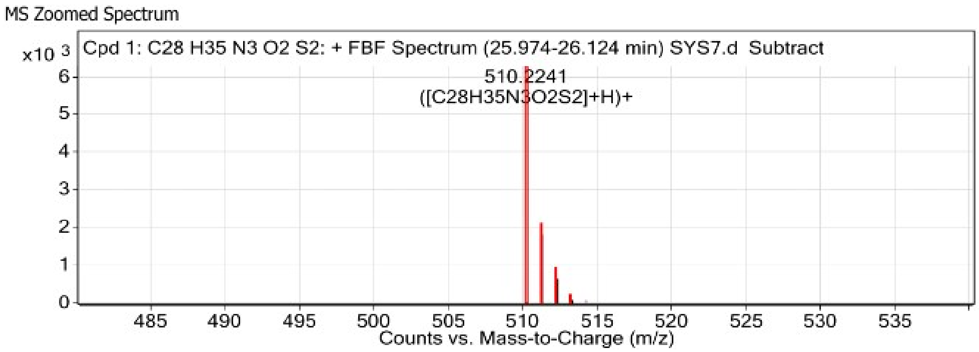

HRMS (ESI) of 5f'

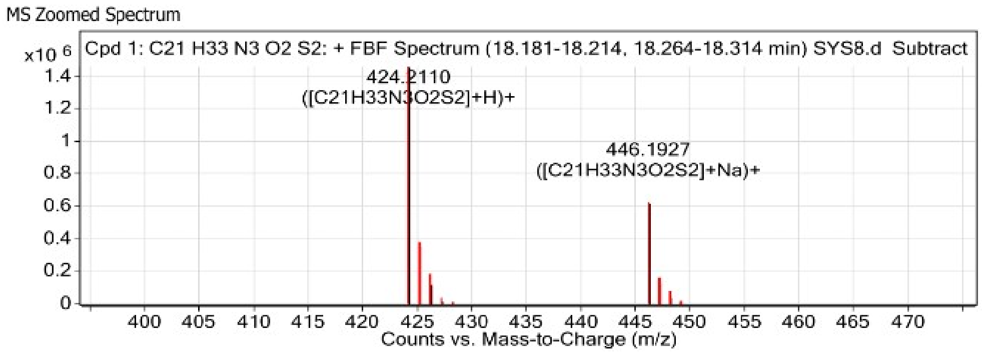

HRMS (ESI) of 5g'

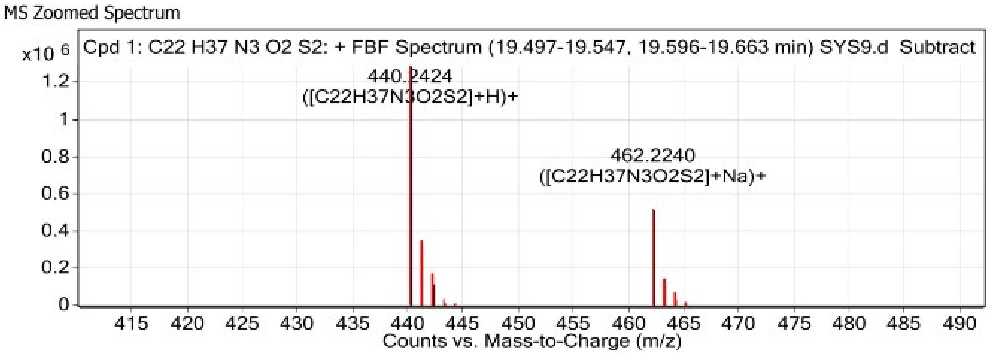

HRMS (ESI) of 5h'

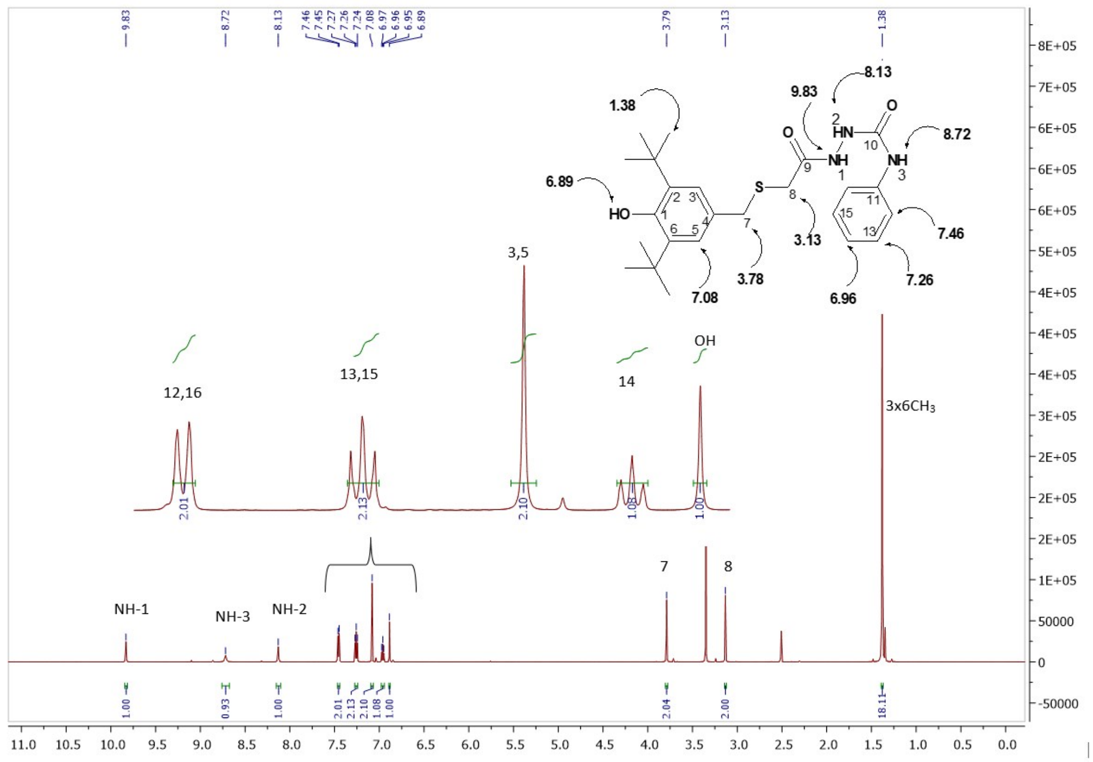

^{1}H spectrum (DMSO-d_6, 600MHz) of 5a

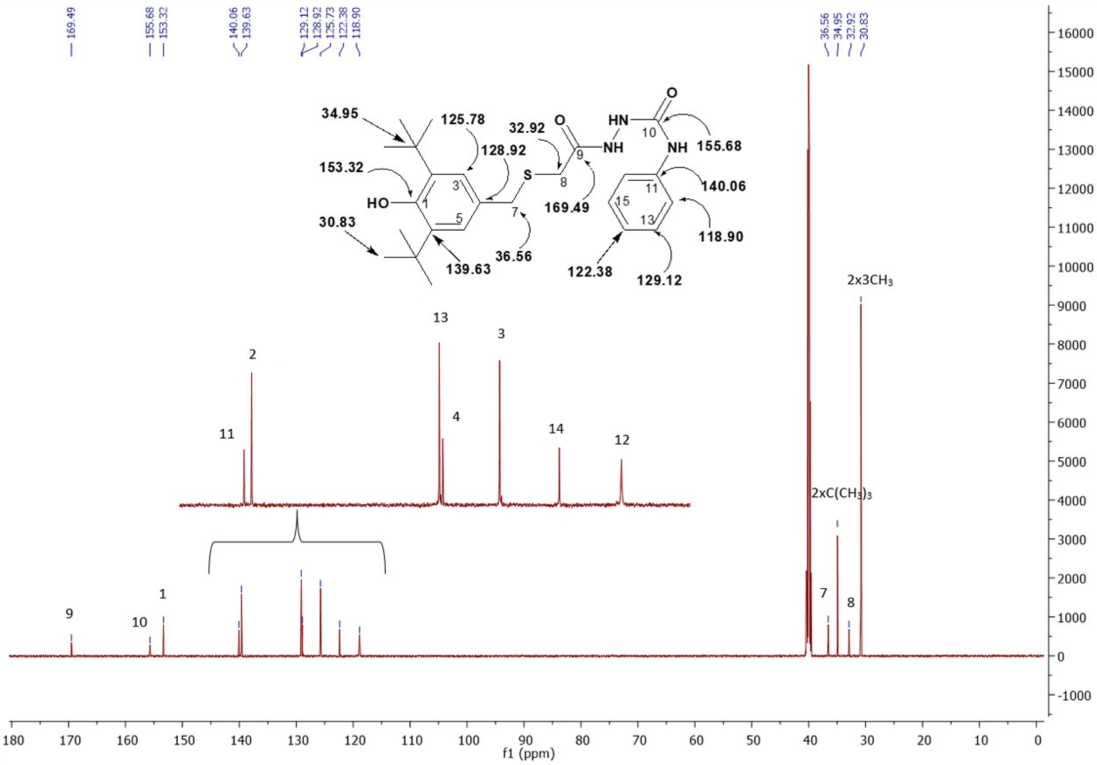

^{13}C spectrum (DMSO-6*d*, 100MHz) of 5a

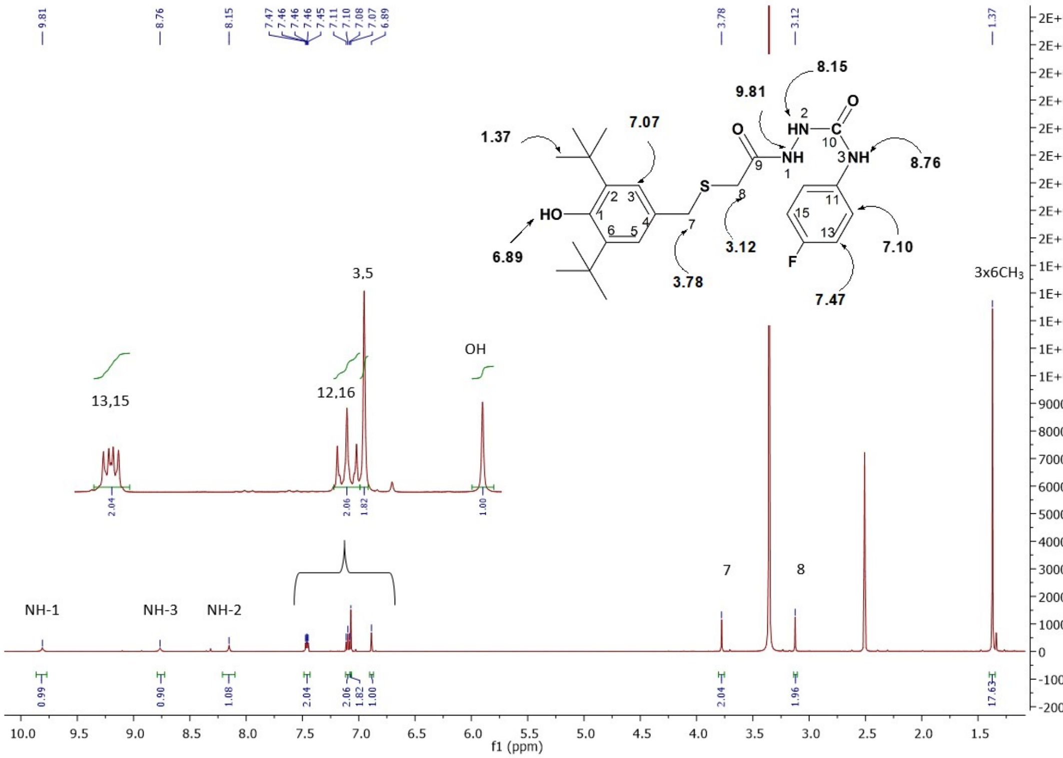

^{1}H spectrum (DMSO-d$_6$, 600MHz) of 5b

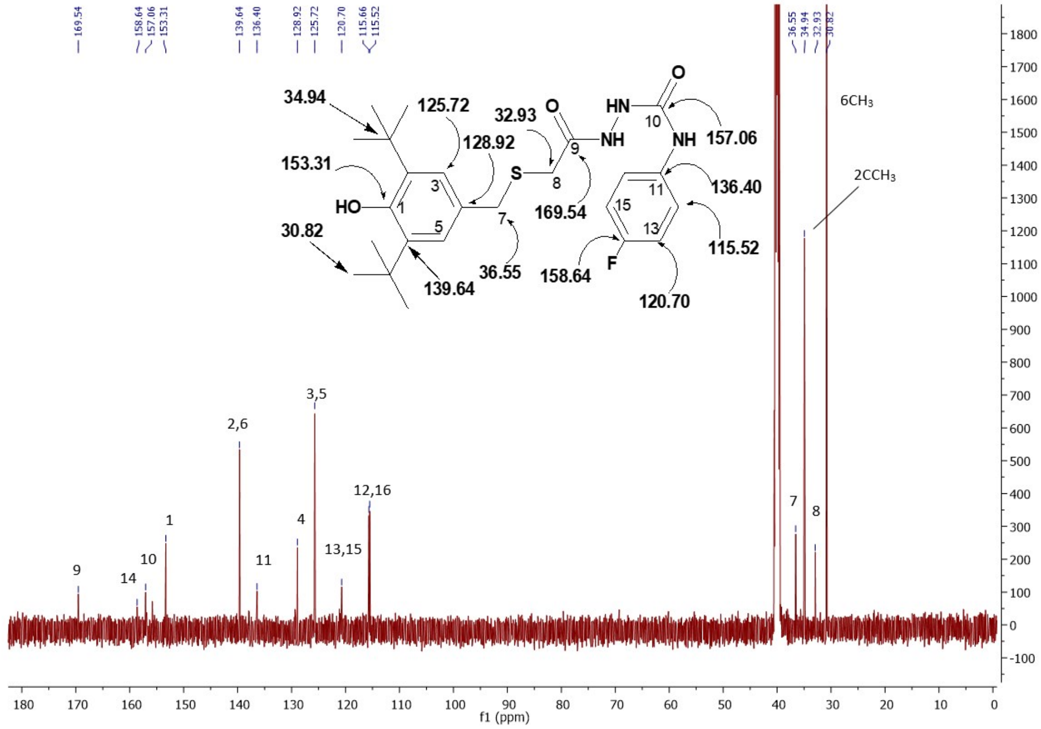

^{13}C spectrum (DMSO-6*d*, 100MHz) of 5b

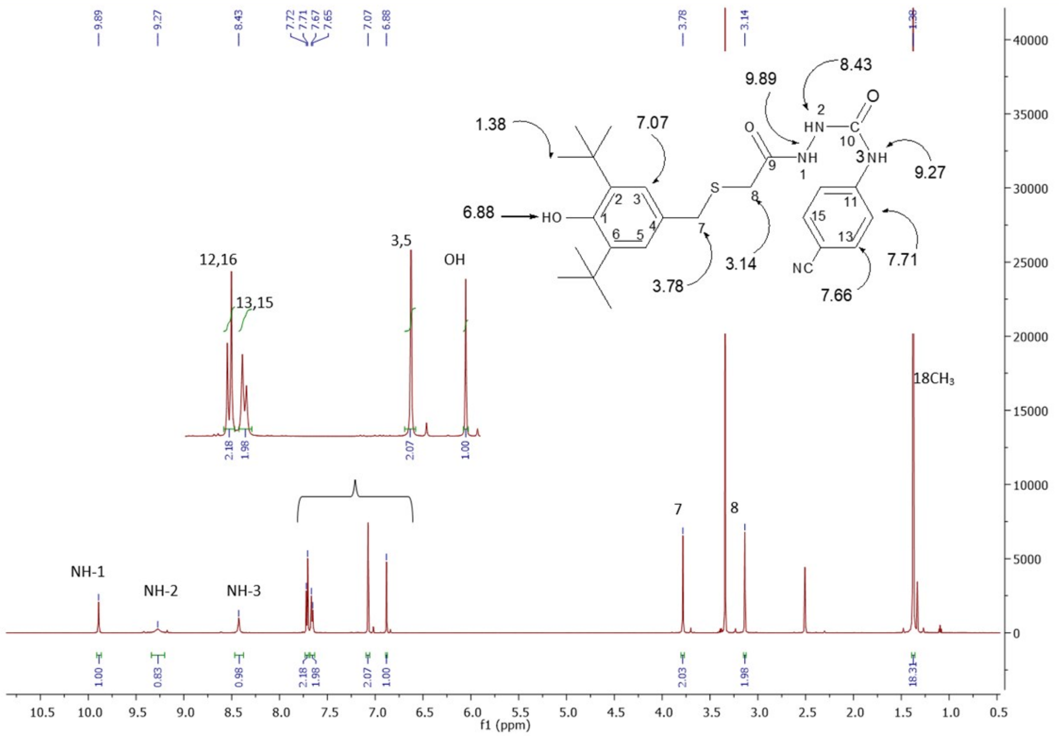

^{1}H spectrum (DMSO-d$_6$, 600MHz) of 5c

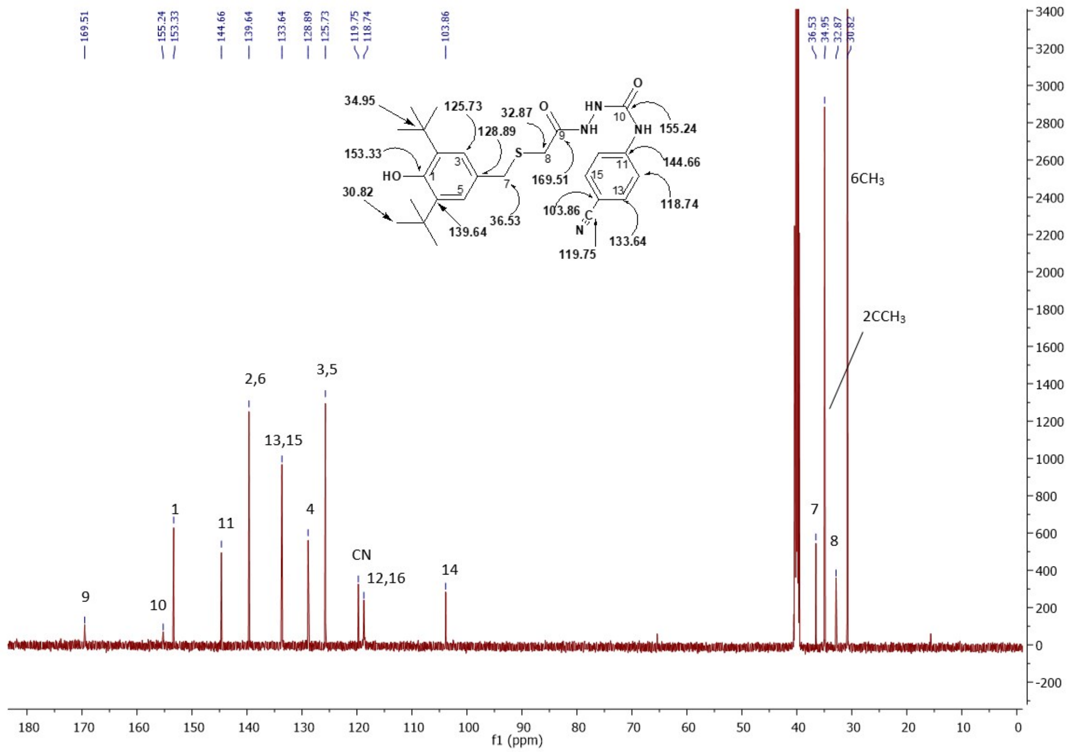

^{13}C spectrum (DMSO-6*d*, 100MHz) of 5c

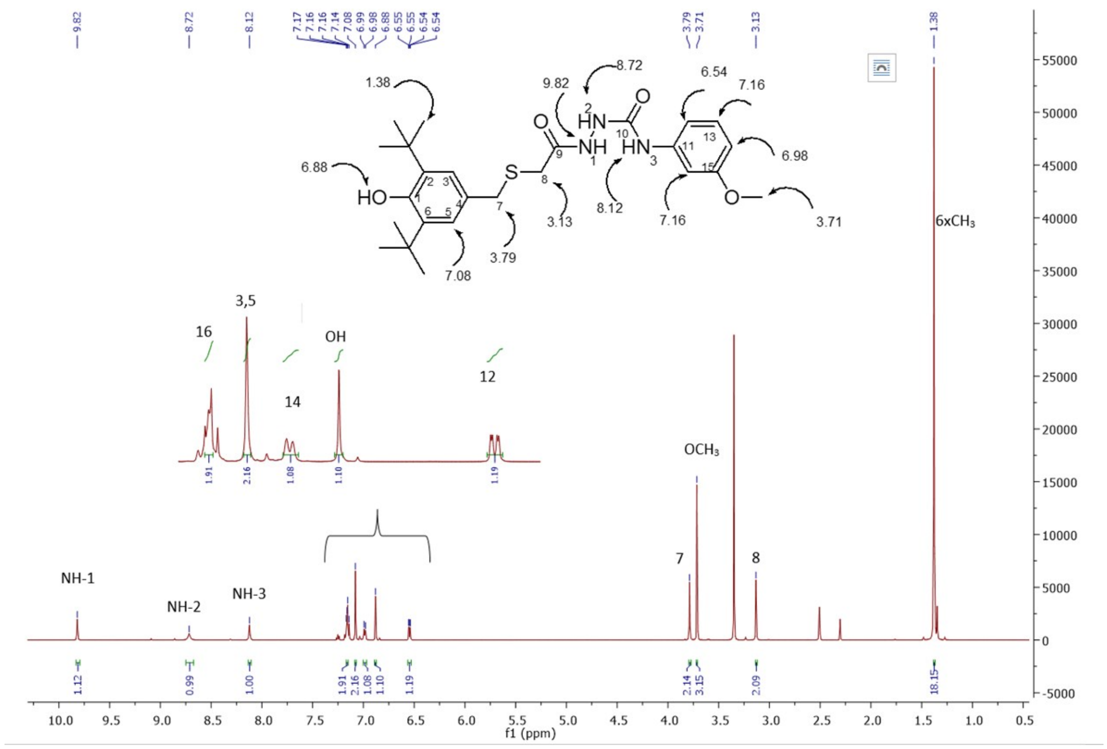

^{1}H spectrum (DMSO-d_6, 600MHz) of 5d

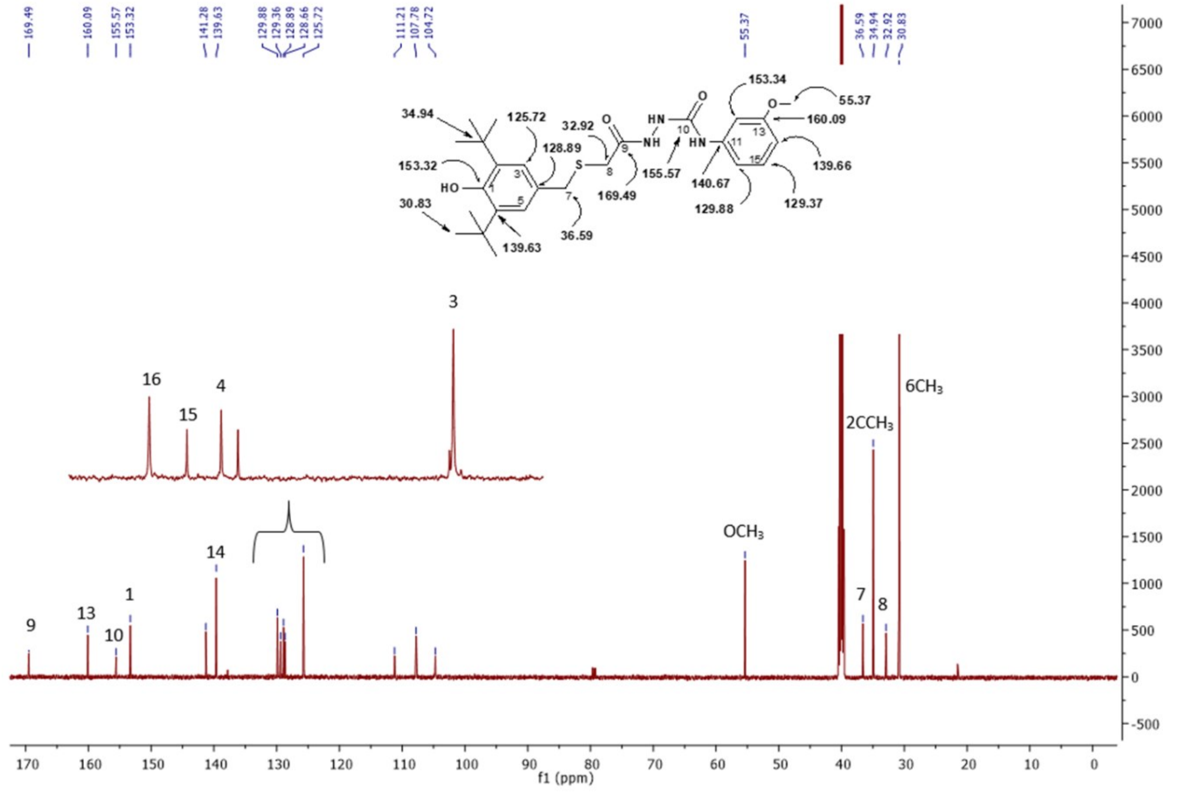

^{13}C spectrum (DMSO-6*d*, 100MHz) of 5d

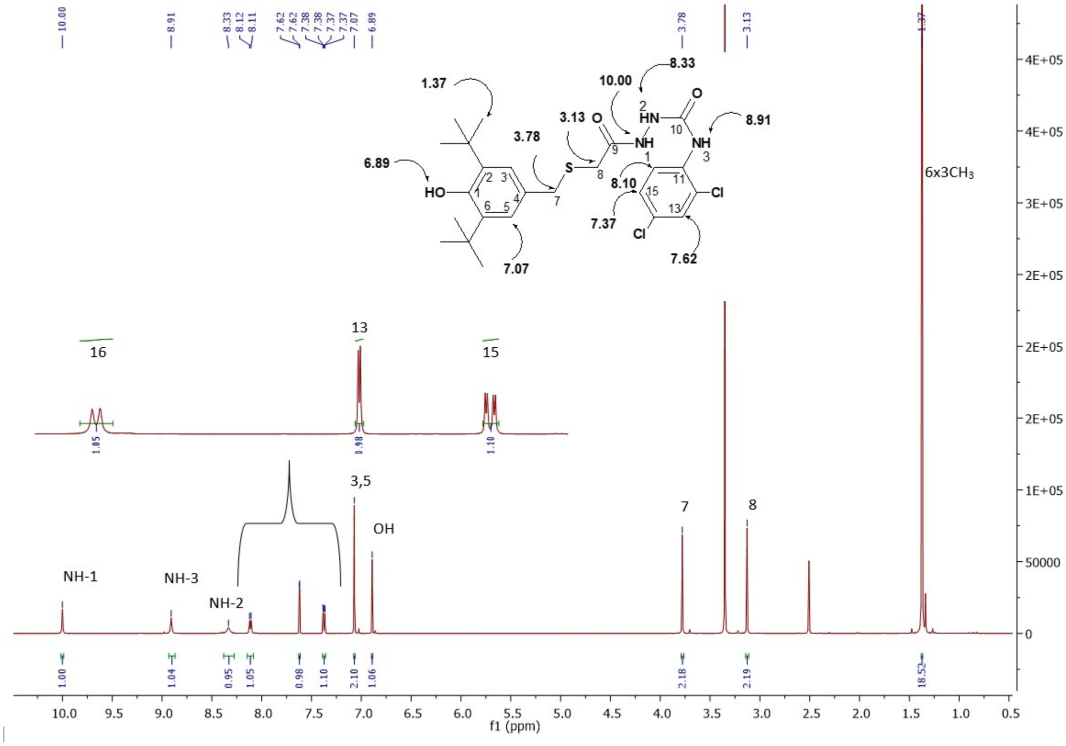

^{1}H spectrum (DMSO-d$_6$, 600MHz) of 5e

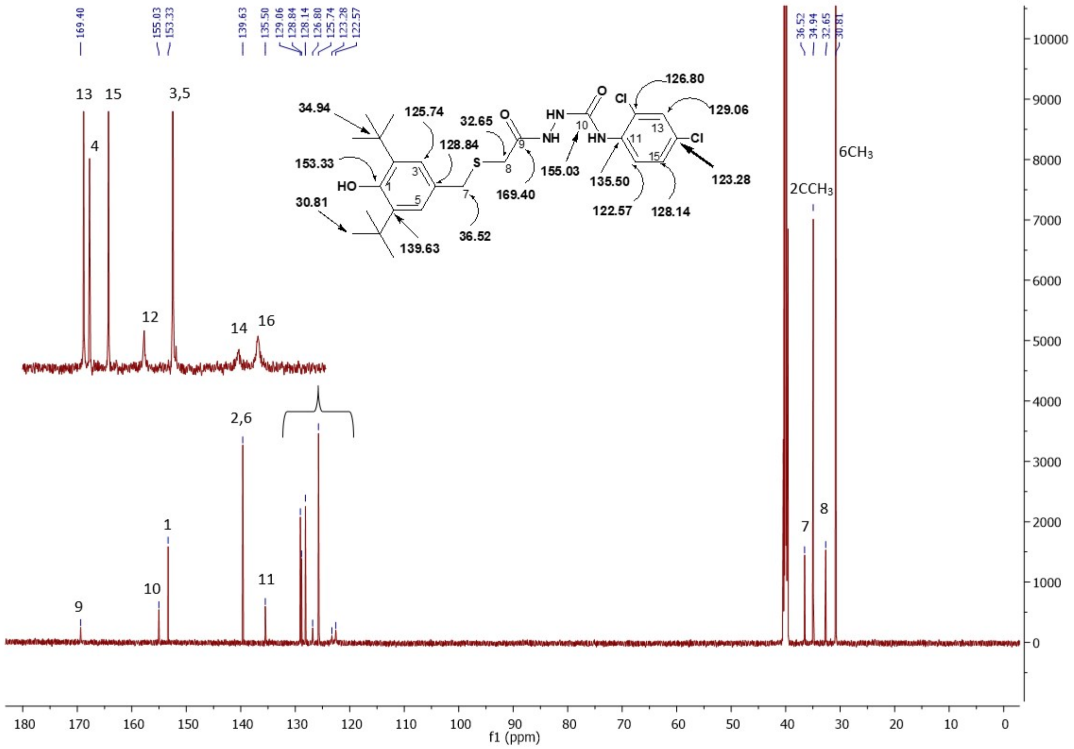

^{13}C spectrum (DMSO-6*d*, 100MHz) of 5e

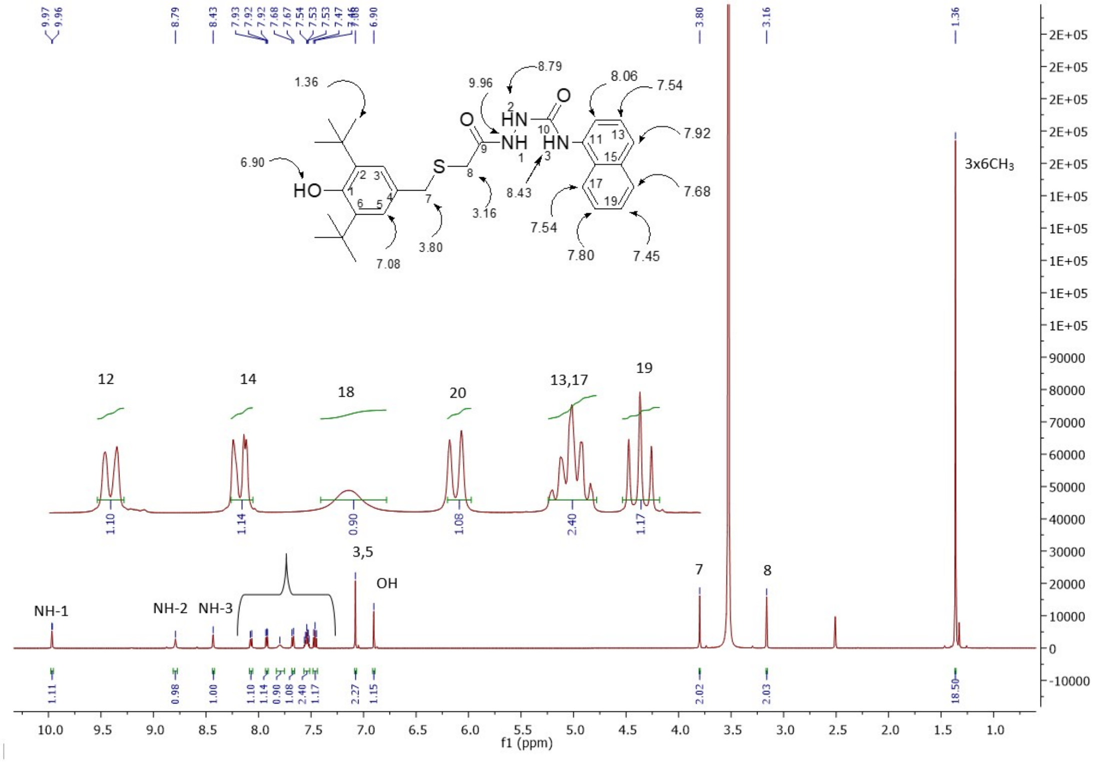

^{1}H spectrum (DMSO-d$_6$, 600MHz) of 5f

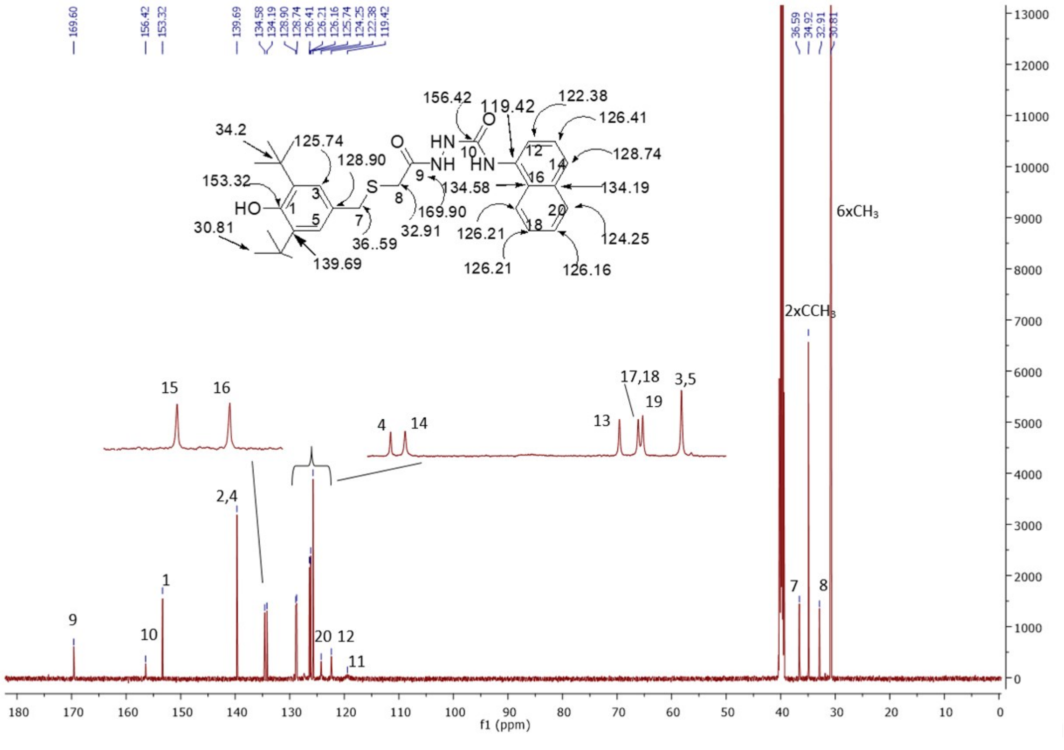

^{13}C spectrum (DMSO-6*d*, 100MHz) of 5f

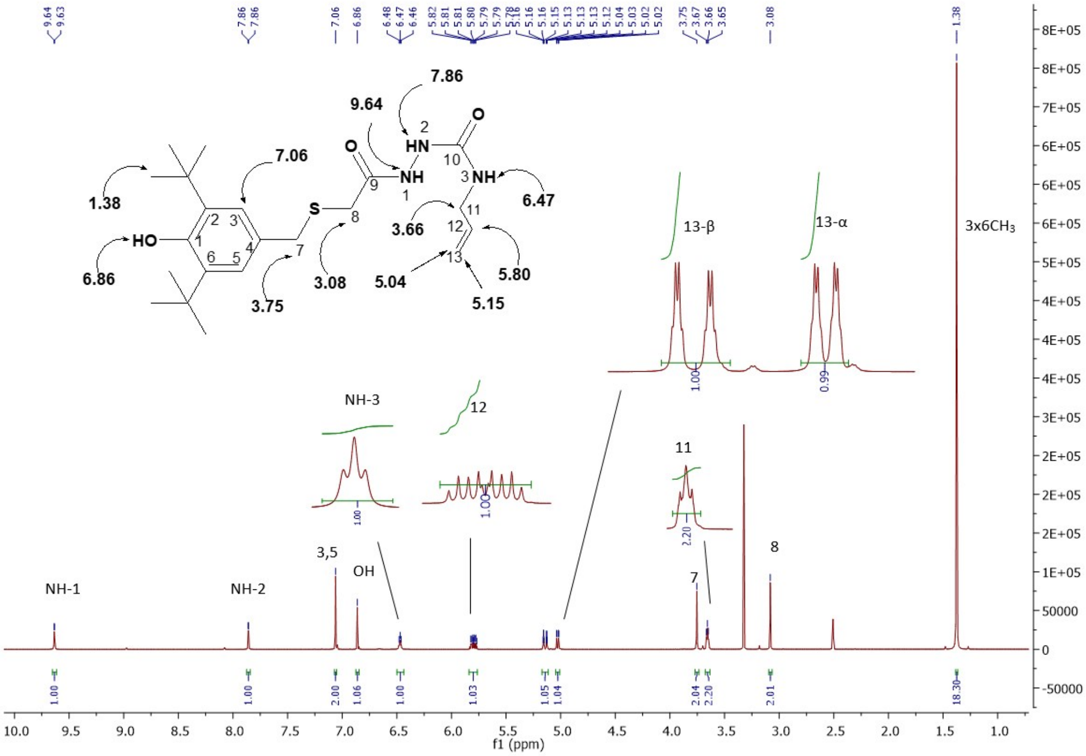

^{1}H spectrum (DMSO-d_6, 600MHz) of 5g

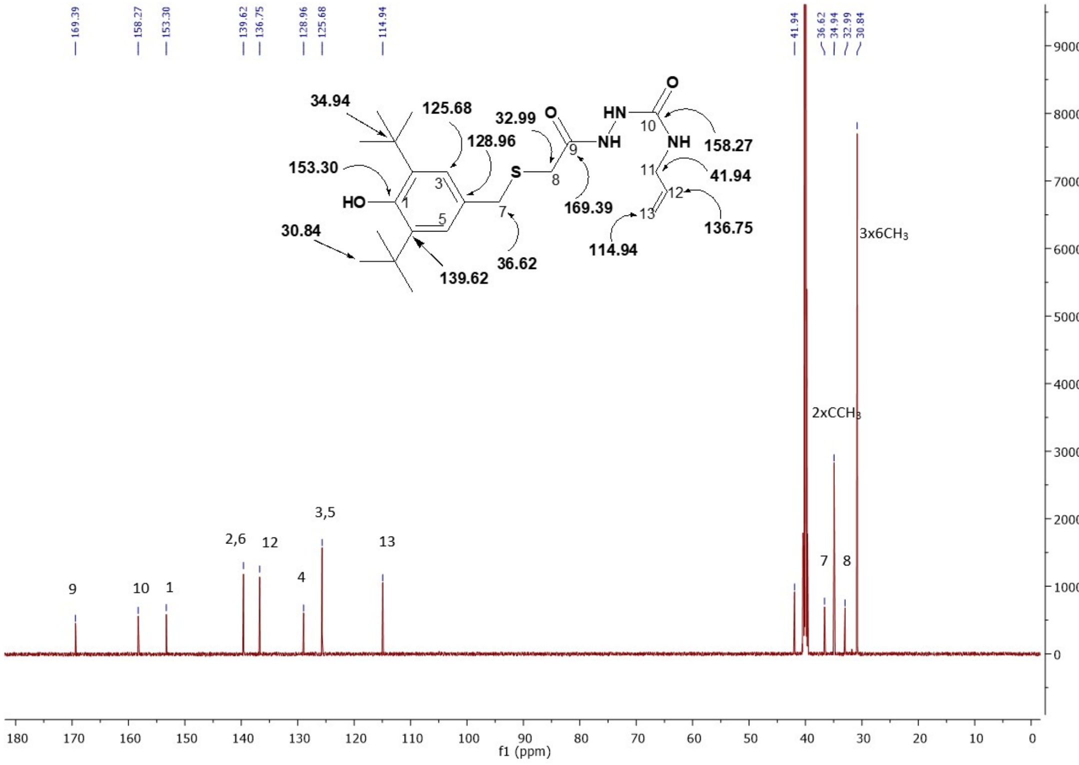

^{13}C spectrum (DMSO-6*d*, 100MHz) of 5g

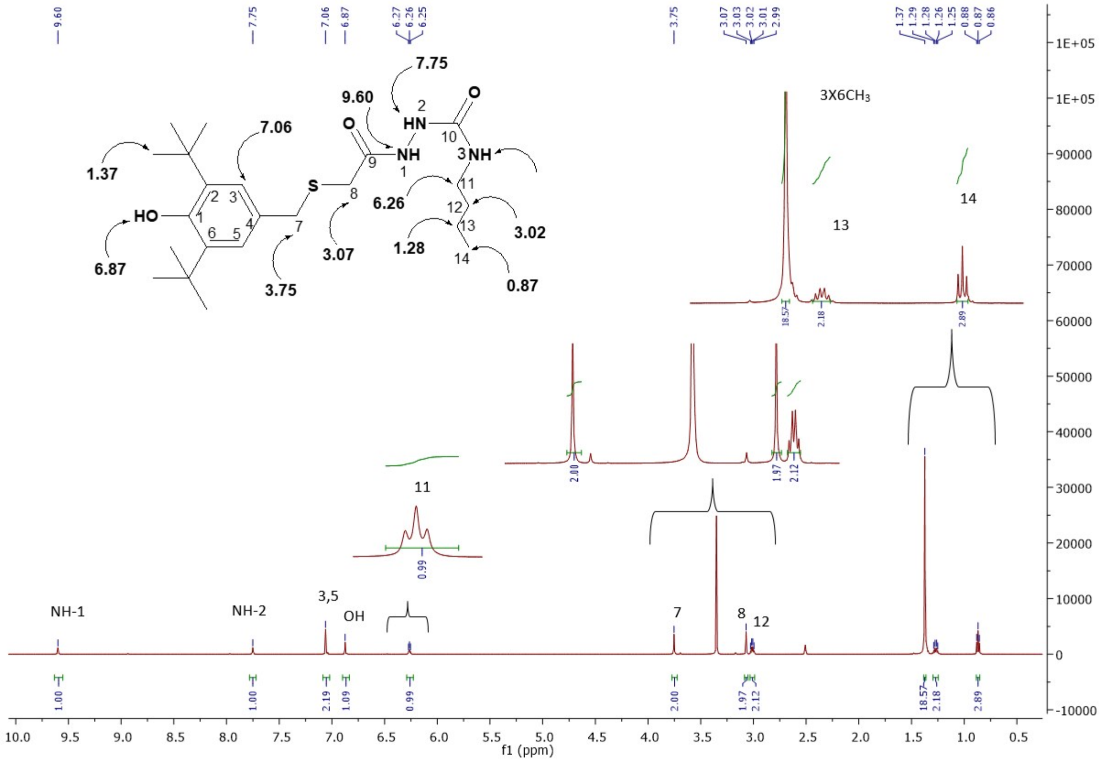

^{1}H spectrum (DMSO-d$_6$, 600MHz) of 5h

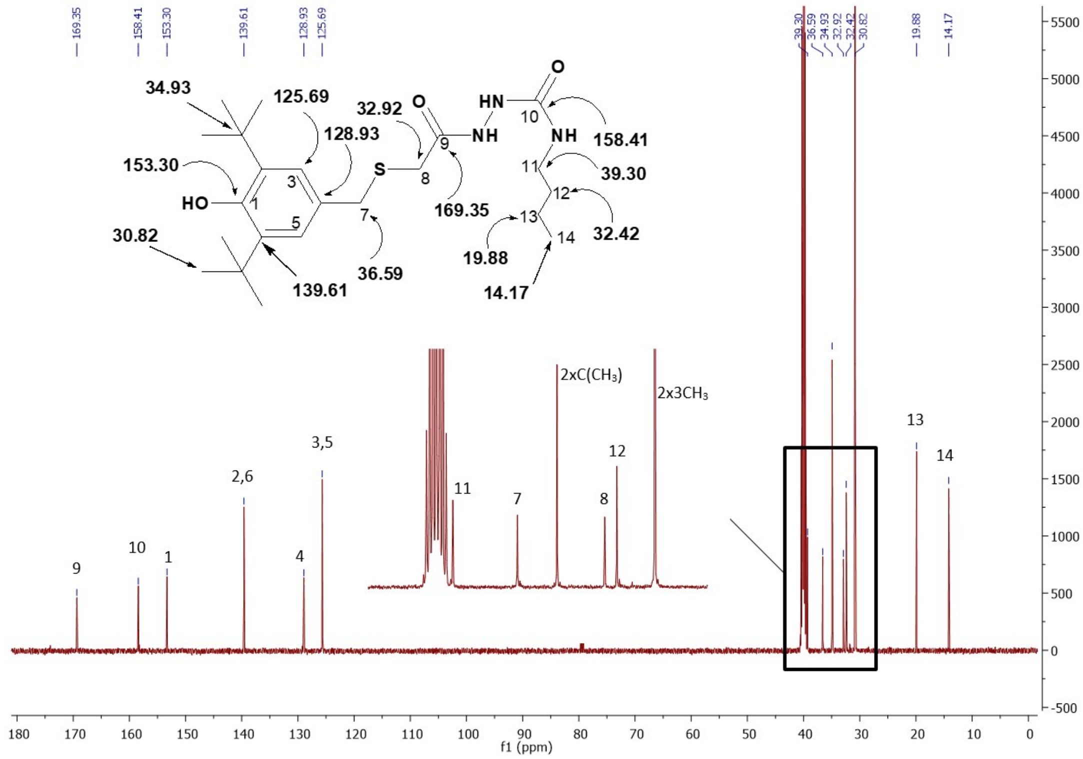

^{13}C spectrum (DMSO-6*d*, 100MHz) of 5h

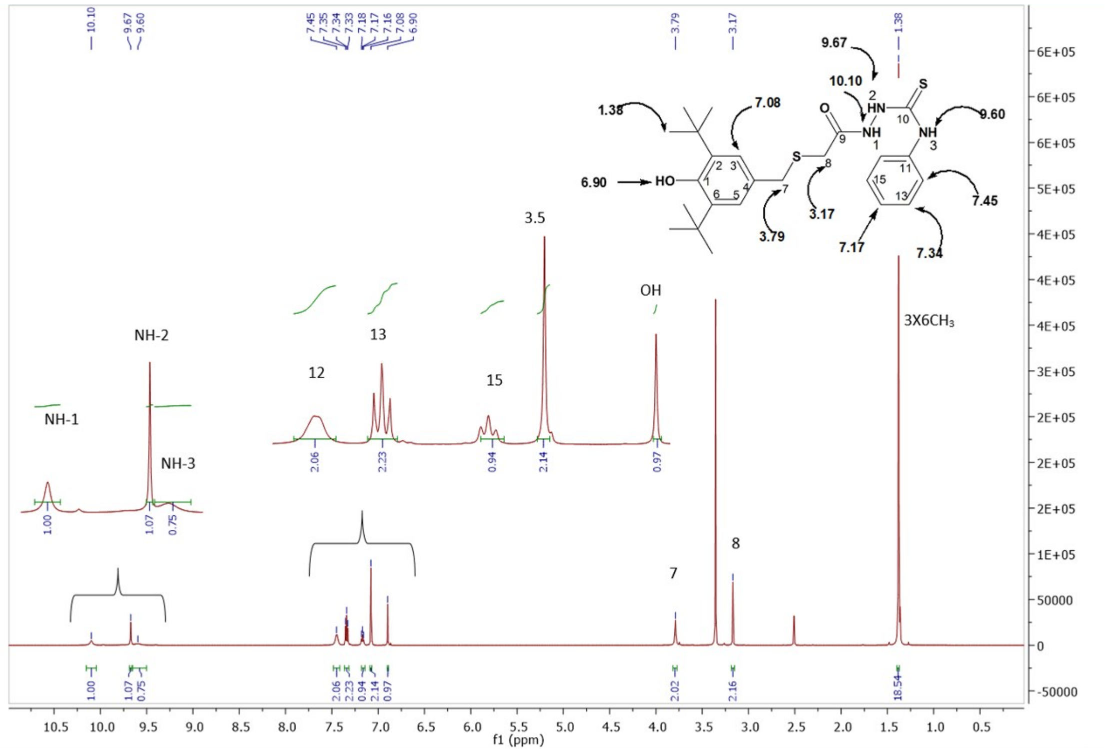

^{1}H spectrum (DMSO-d$_6$, 600MHz) of 5a’

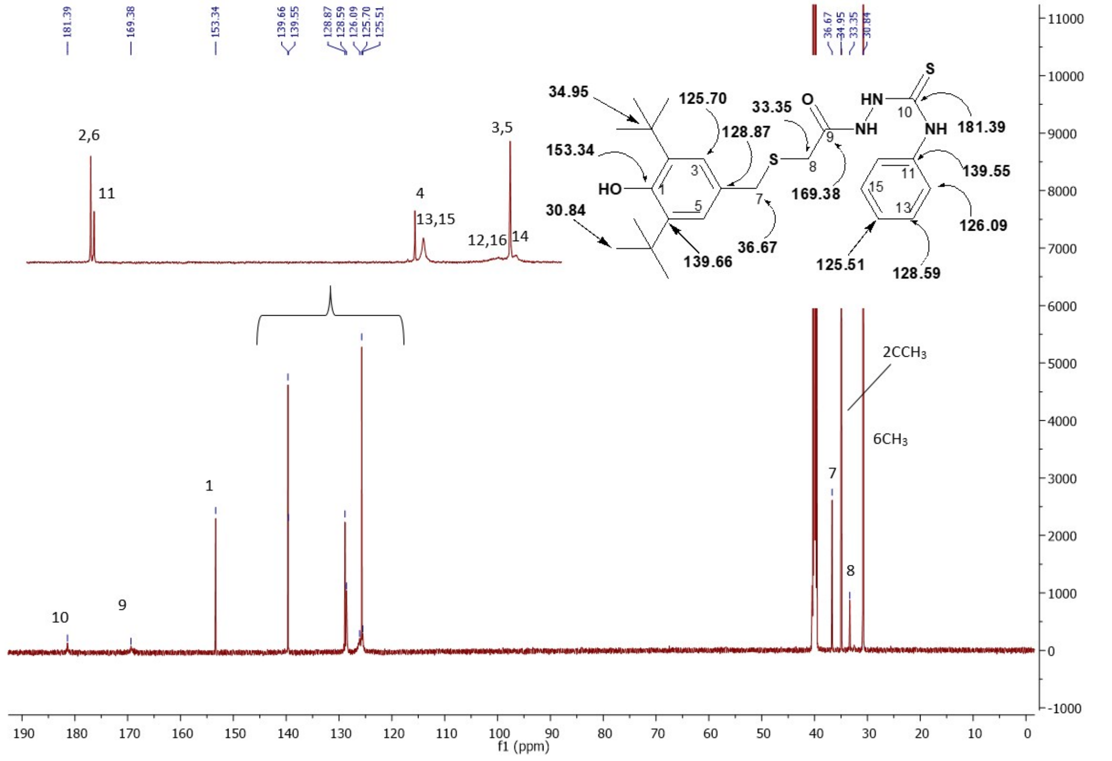

^{13}C spectrum (DMSO-6*d*, 100MHz) of 5a’

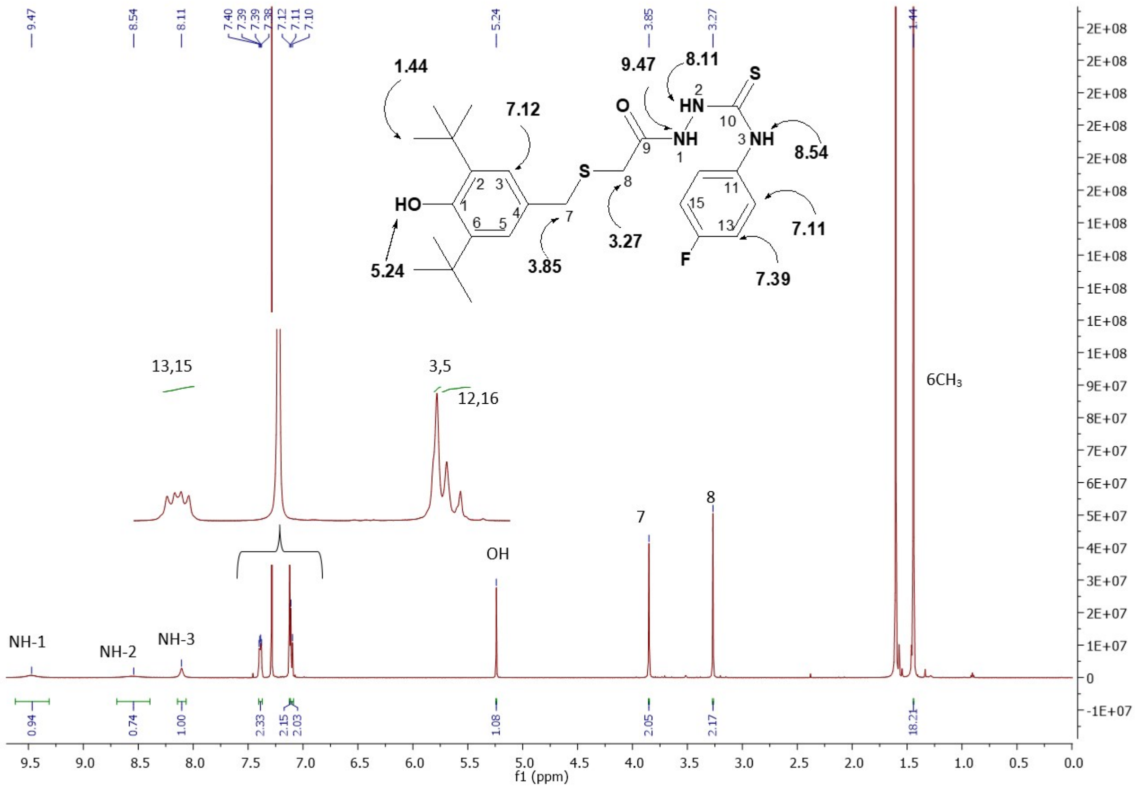

^{1}H spectrum ($CDCl_3$, 600MHz) of 5b’

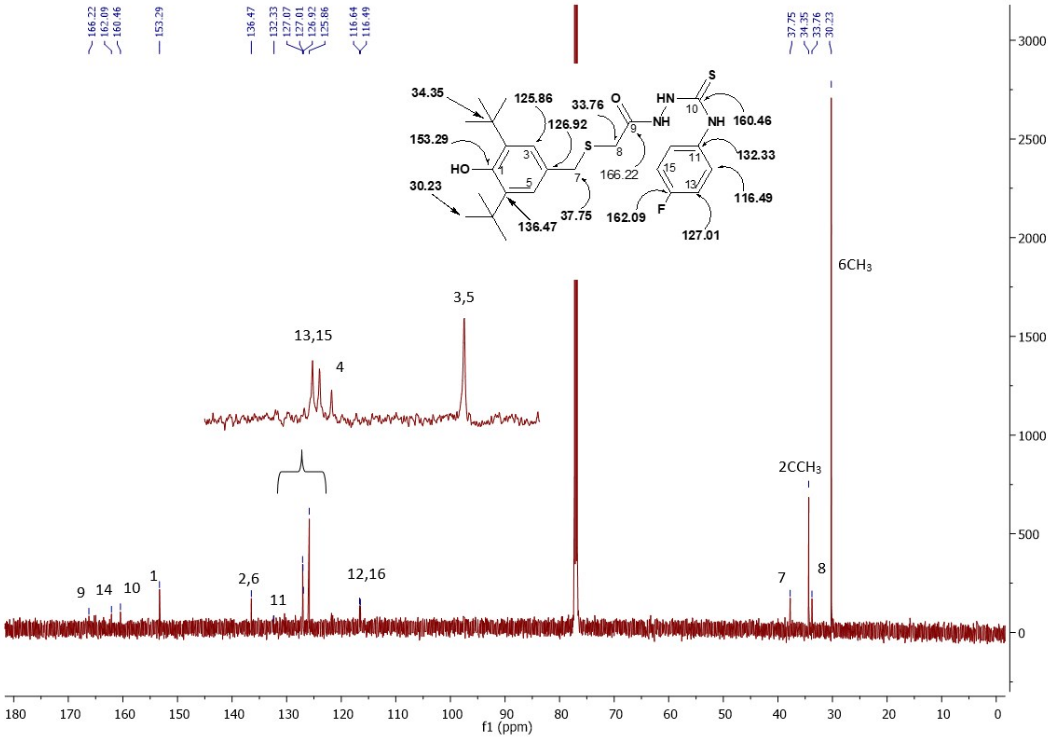

^{13}C spectrum (DMSO-6*d*, 100MHz) of 5b’

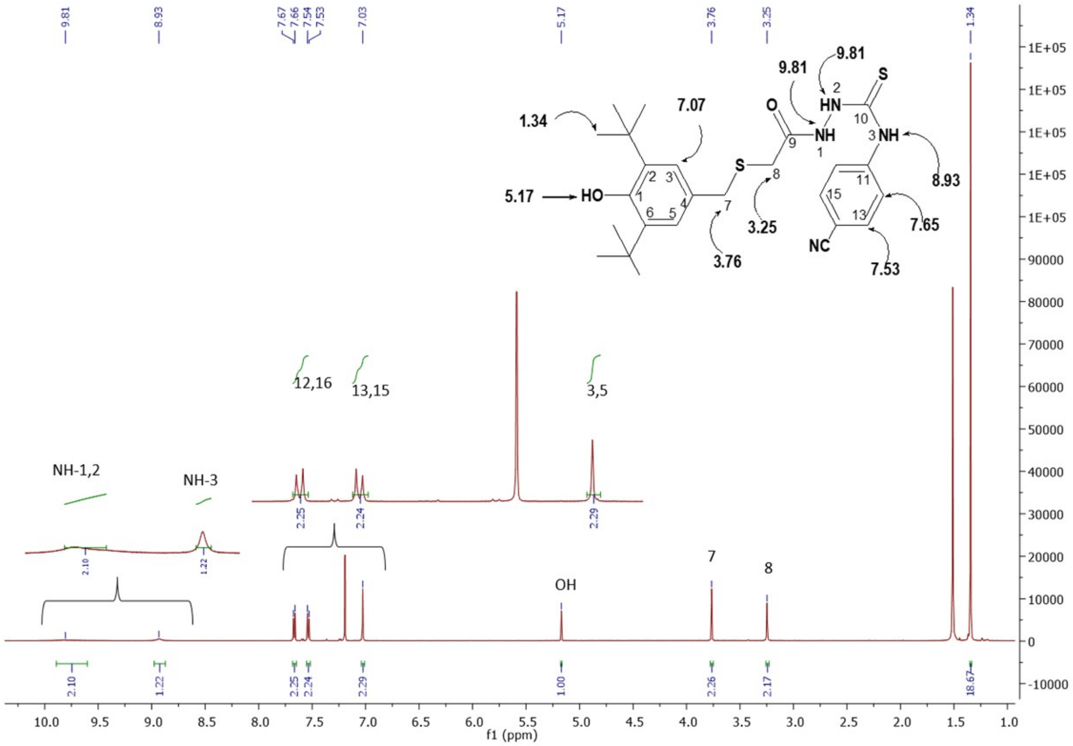

^{1}H spectrum ($CDCl_3$, 600MHz) of 5c'

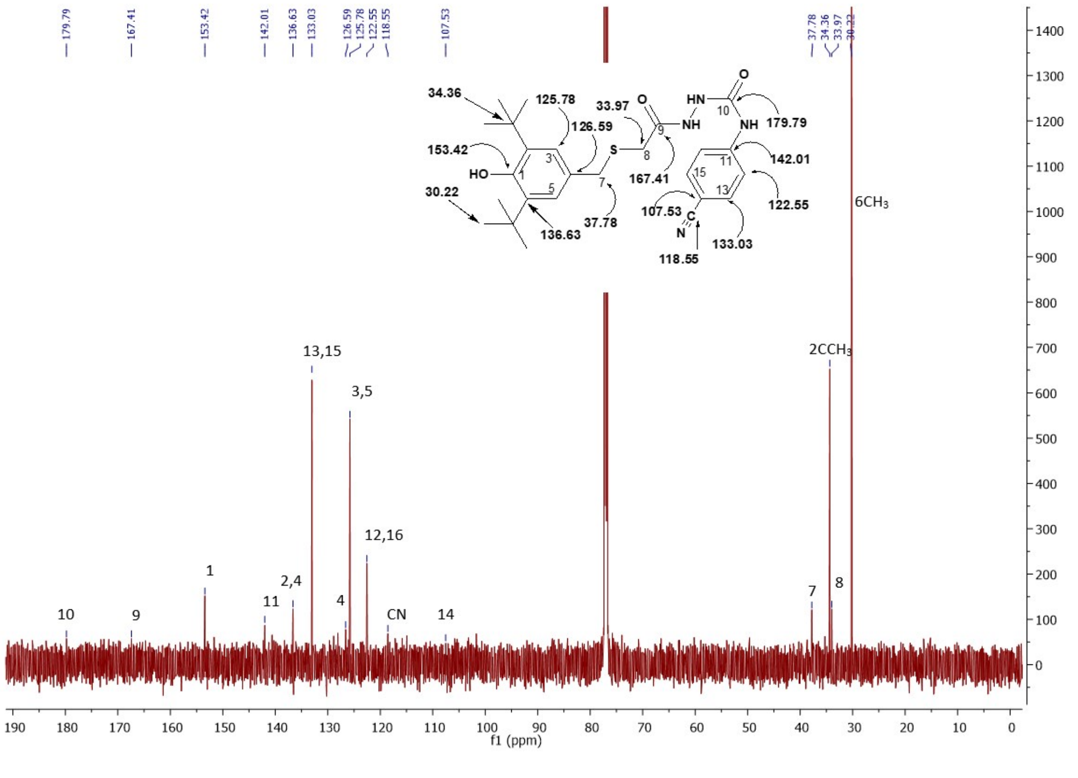

^{13}C spectrum (DMSO-6*d*, 100MHz) of 5c'

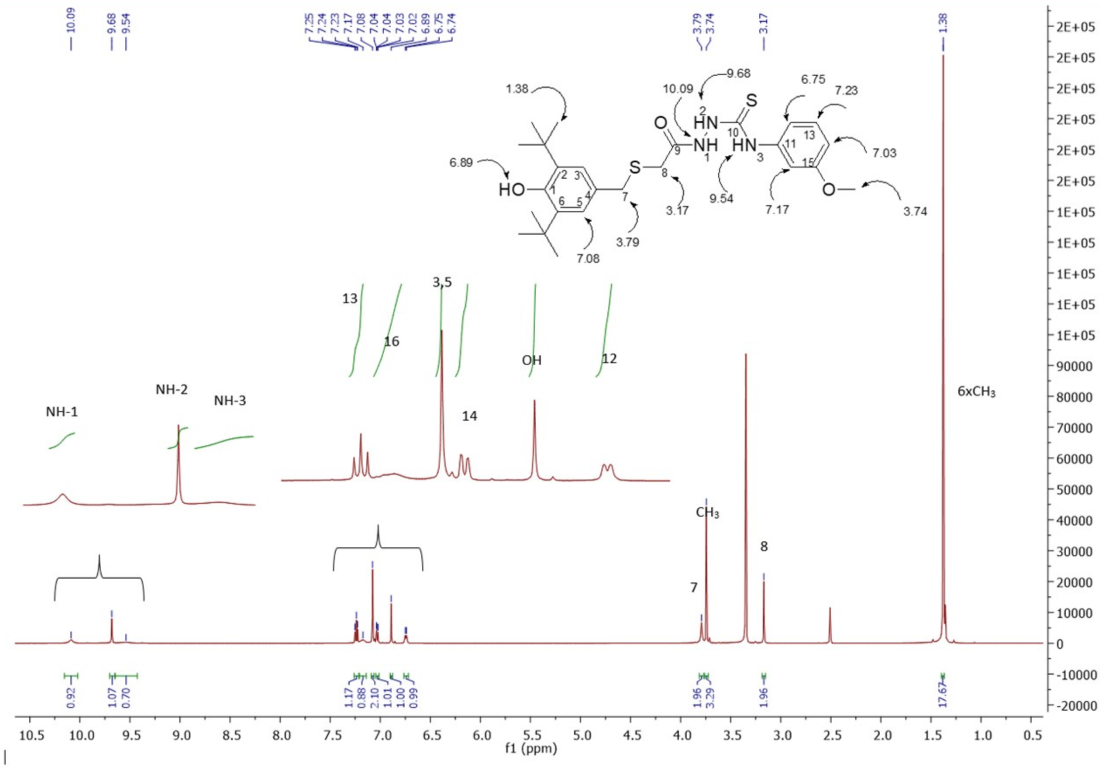

^{1}H spectrum (DMSO-d_6, 600MHz) of 5d'

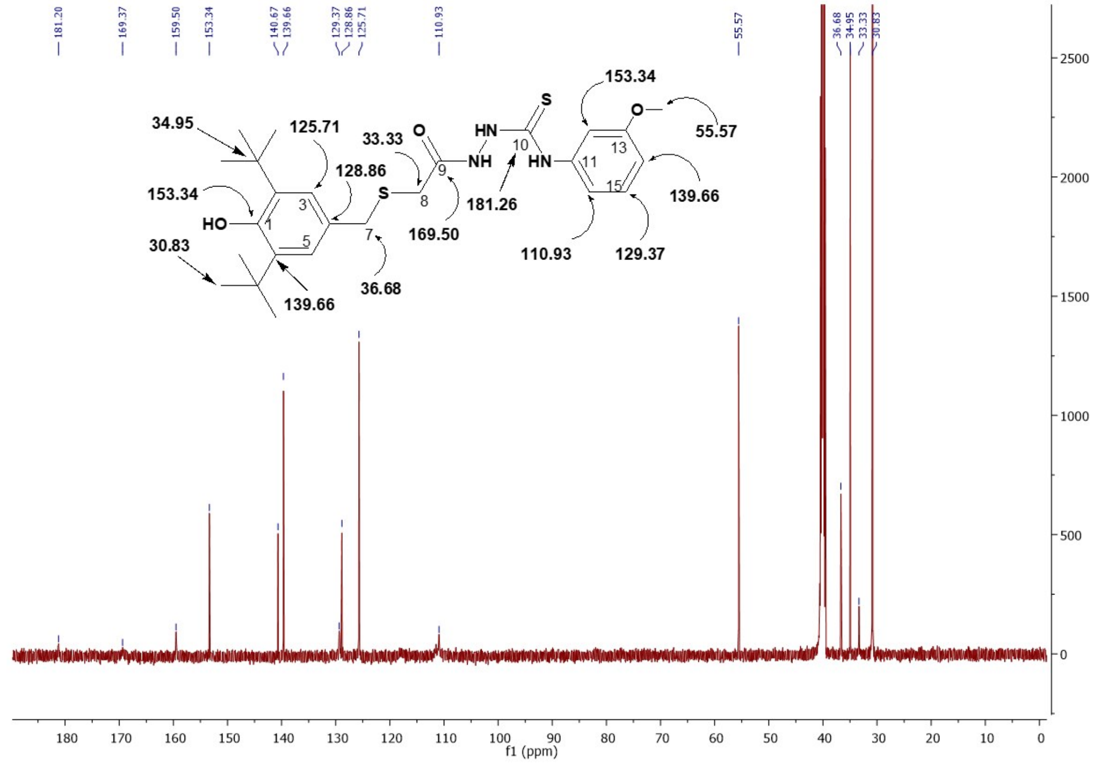

^{13}C spectrum (DMSO-6*d*, 100MHz) of 5d'

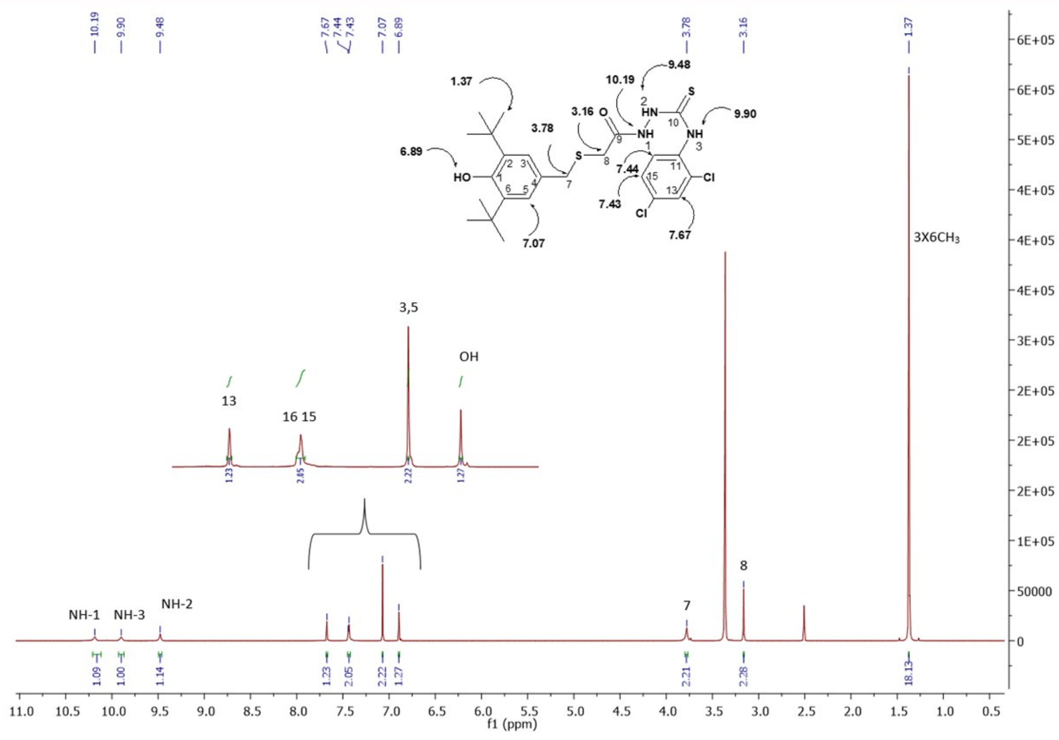

^{1}H spectrum (DMSO-d$_6$, 600MHz) of 5e'

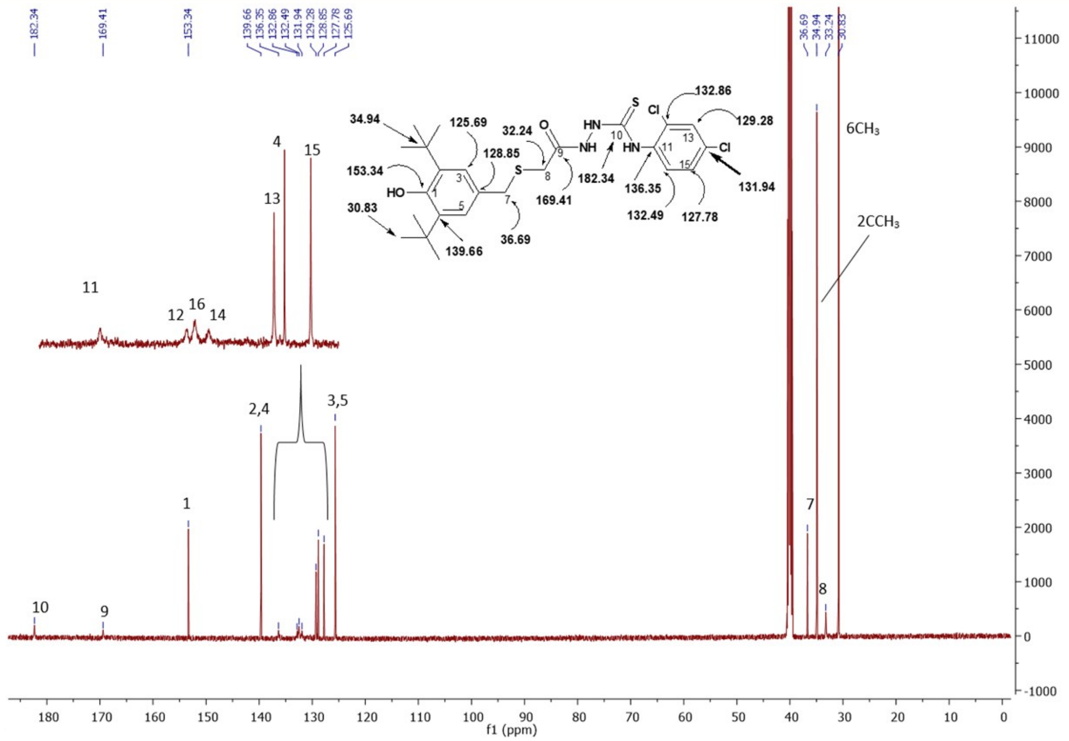

^{13}C spectrum (DMSO-6*d*, 100MHz) of 5e'

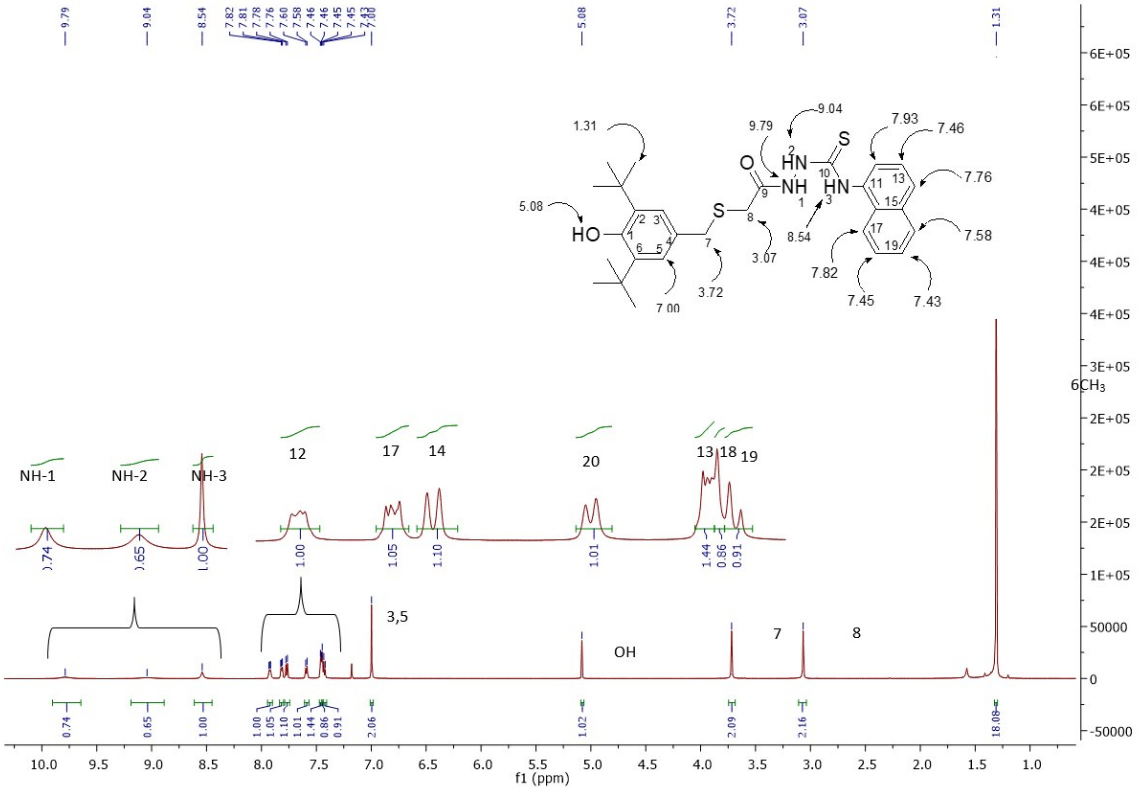

^{1}H spectrum (CDCl$_3$, 600MHz) of 5f'

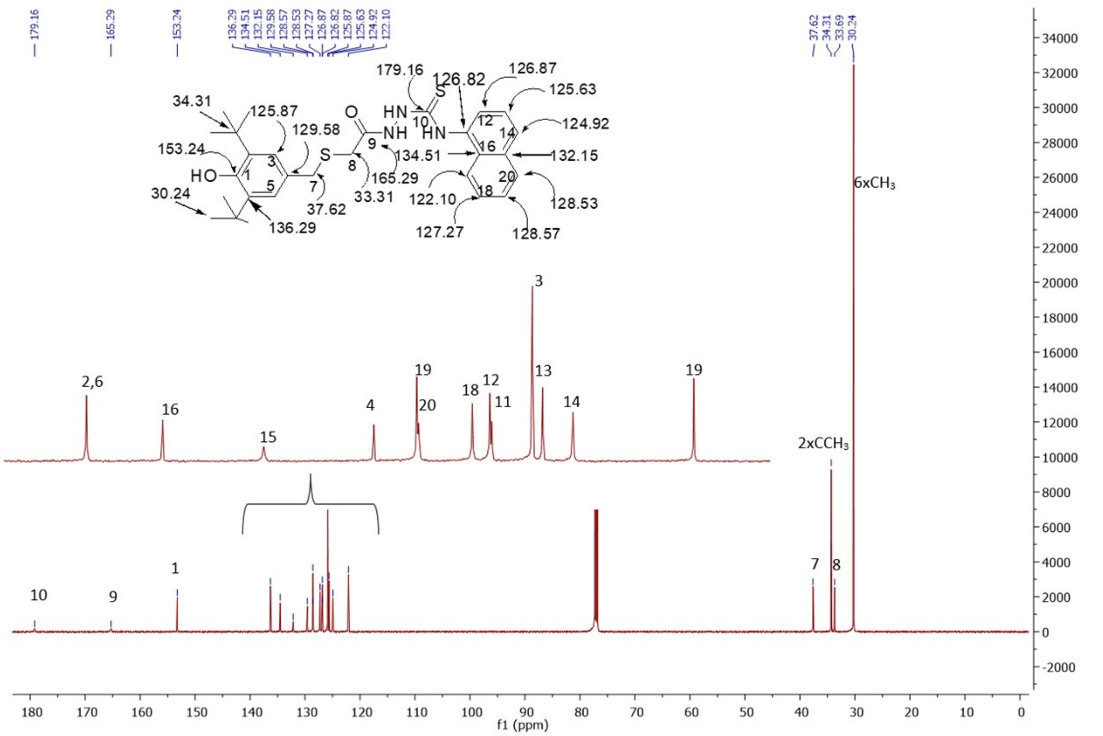

^{13}C spectrum (DMSO-6*d*, 100MHz) of 5f'

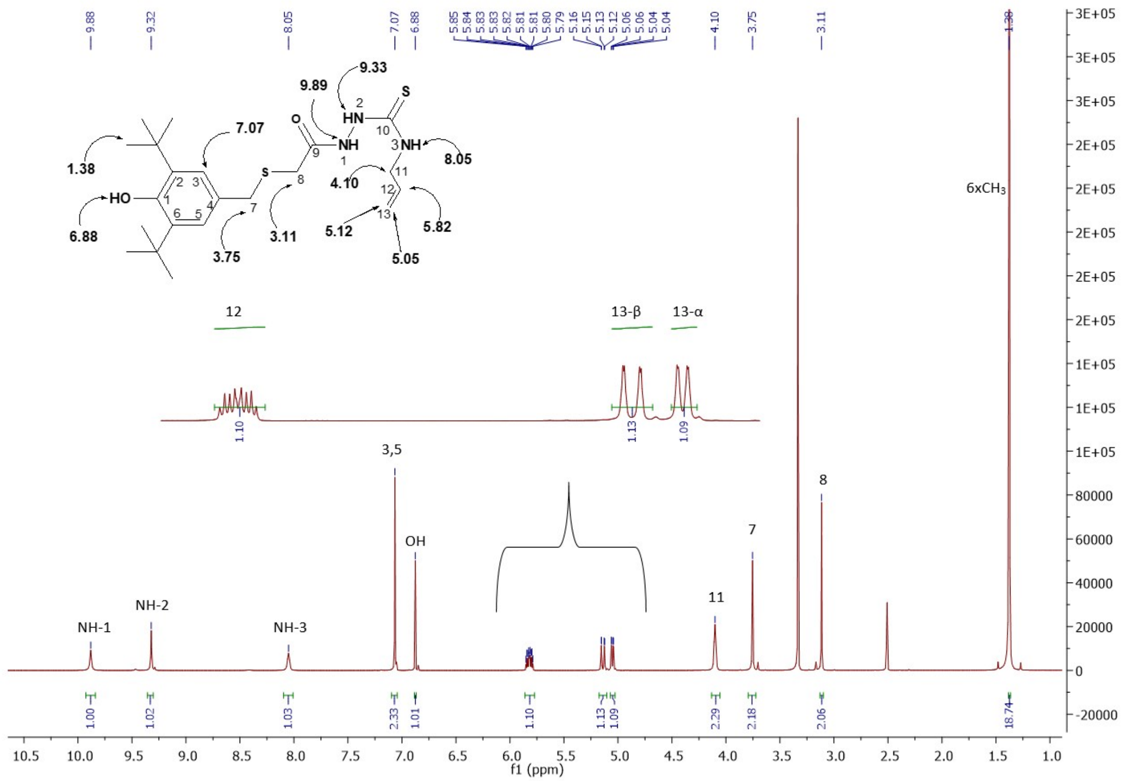

^{1}H spectrum (DMSO-d_6, 600MHz) of 5g'

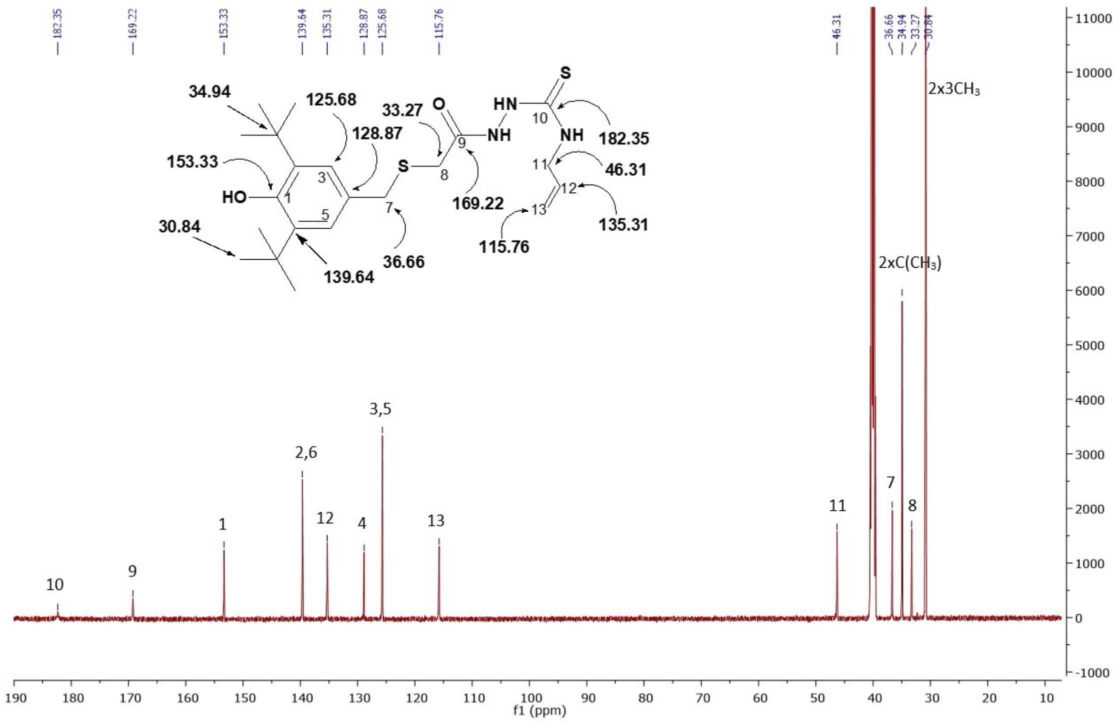

^{13}C spectrum (DMSO-6*d*, 100MHz) of 5g'

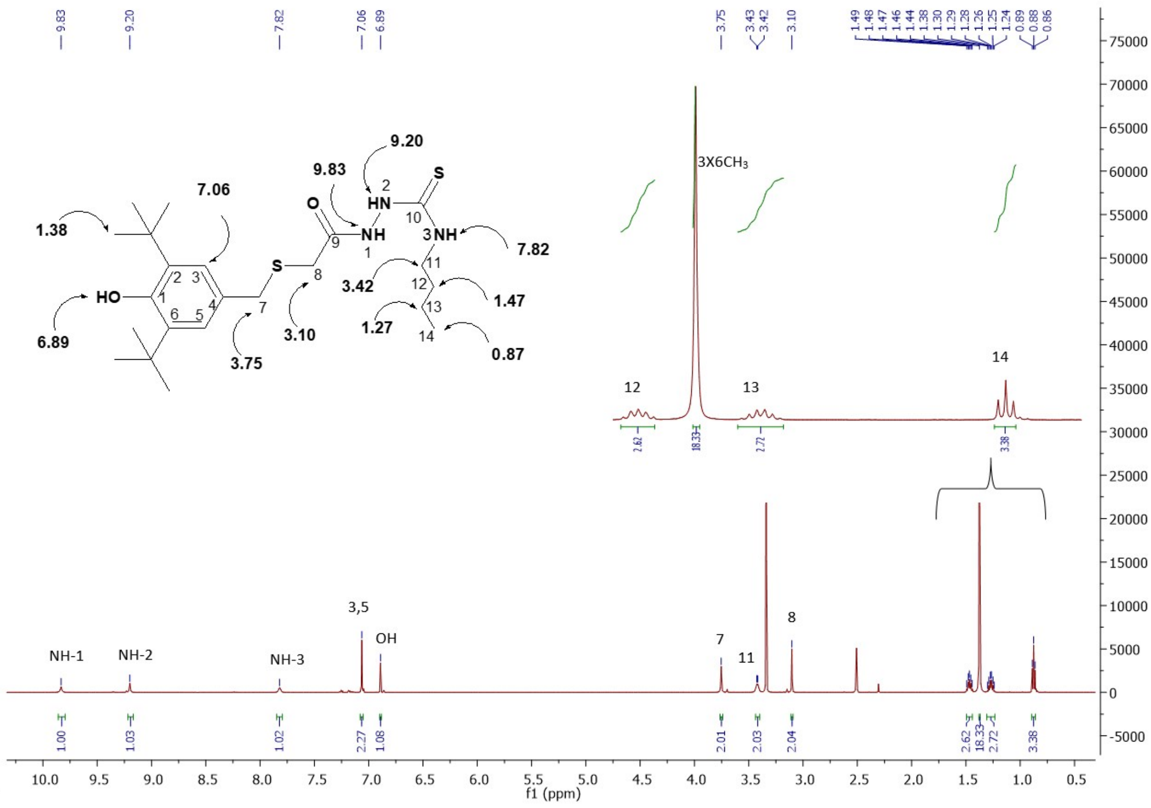

^{1}H spectrum (DMSO-d$_6$, 600MHz) of 5h'

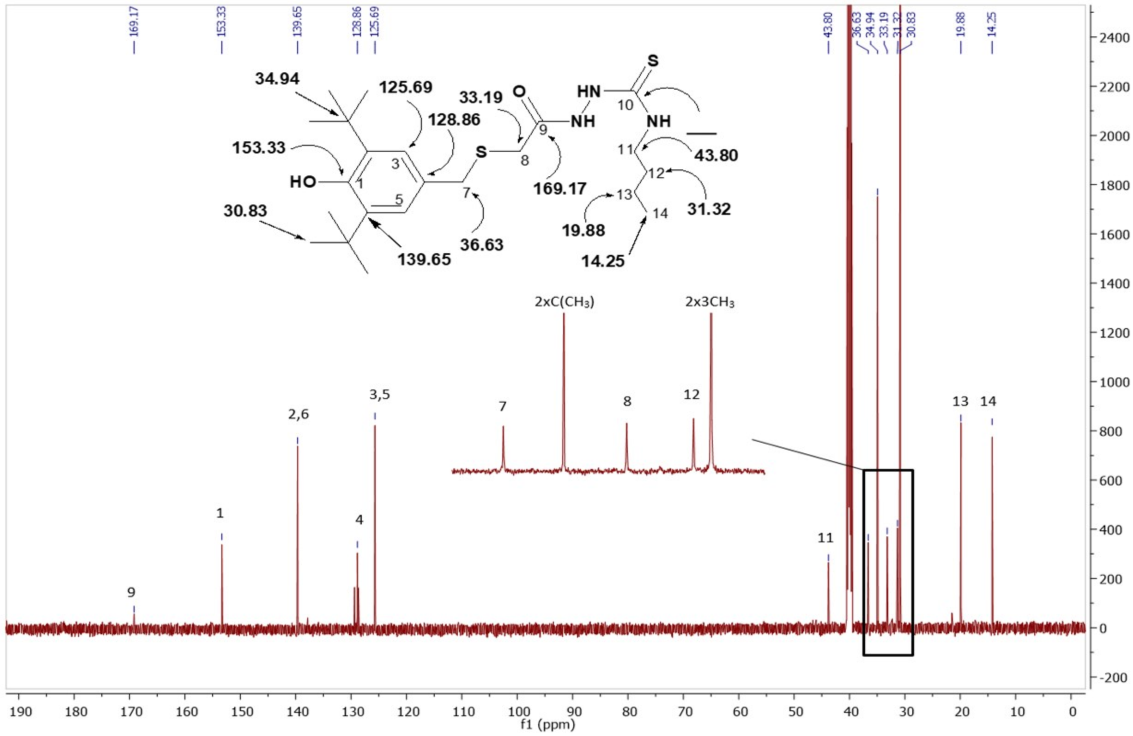

^{13}C spectrum (DMSO-6*d*, 100MHz) of 5h'

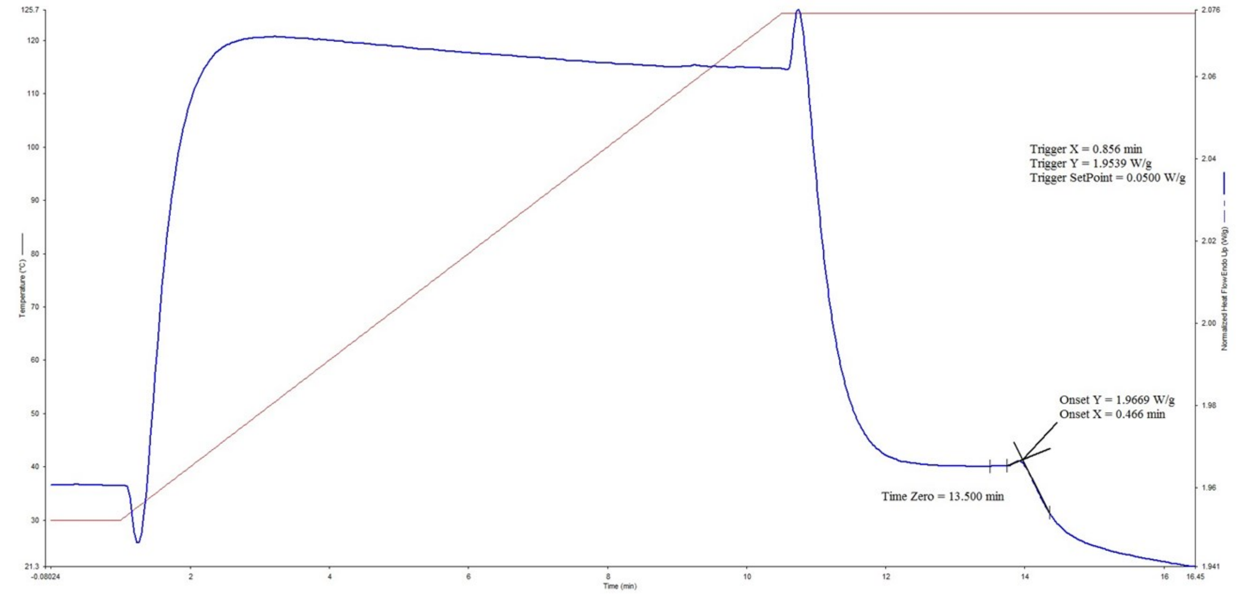

DSC OIT of TMPTO

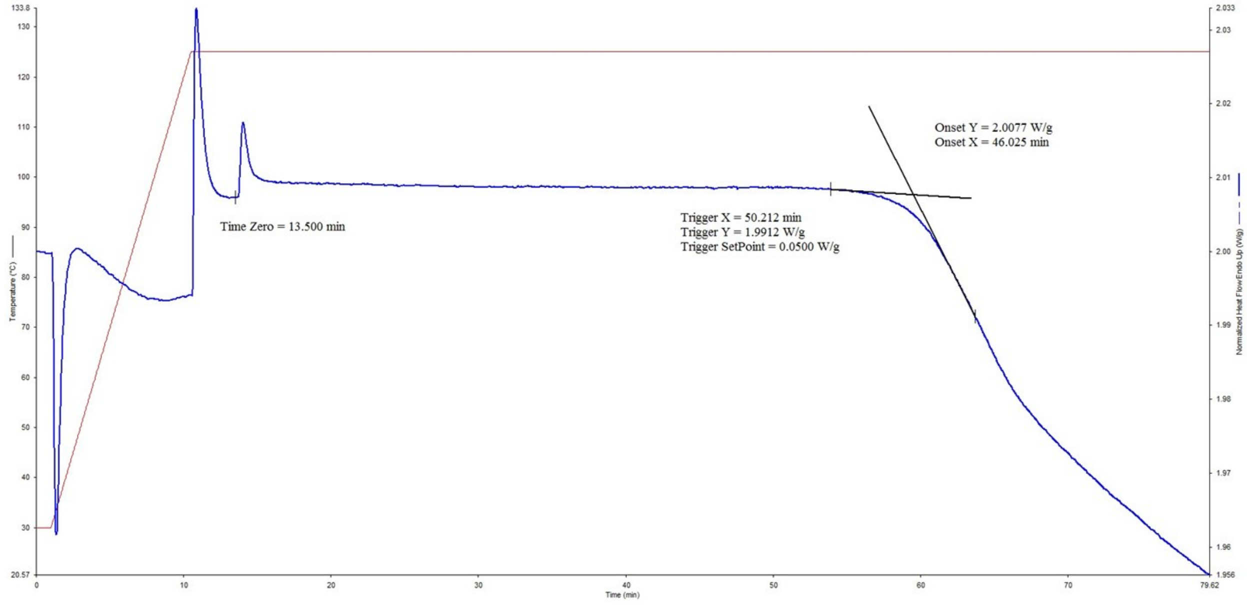

DSC OIT of TMPTO + BHT

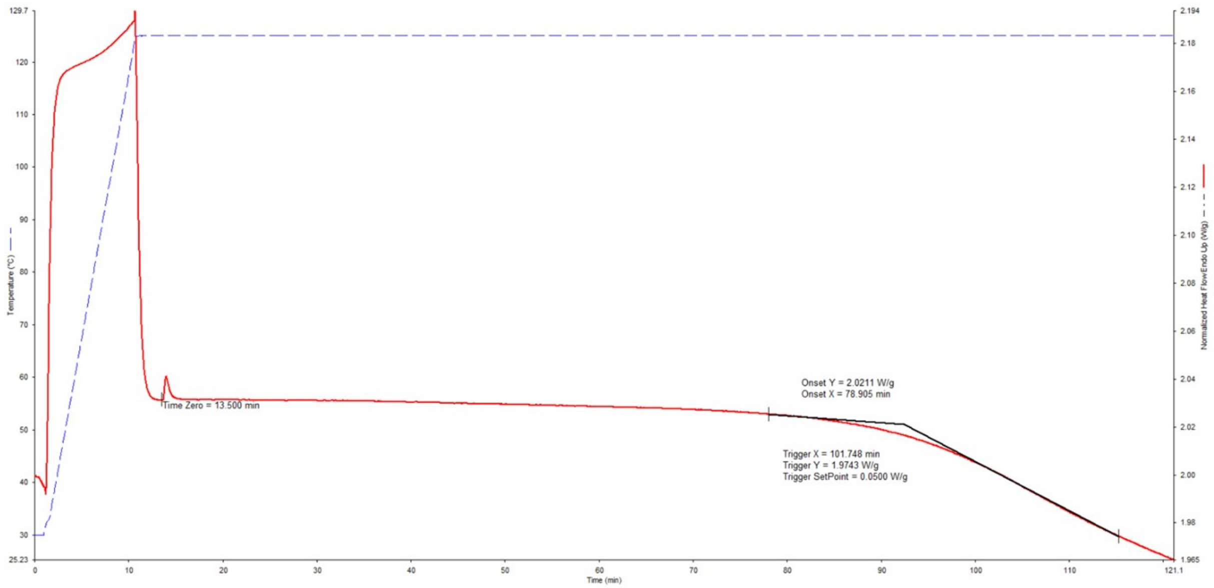

DSC OIT of TMPTO + 5f

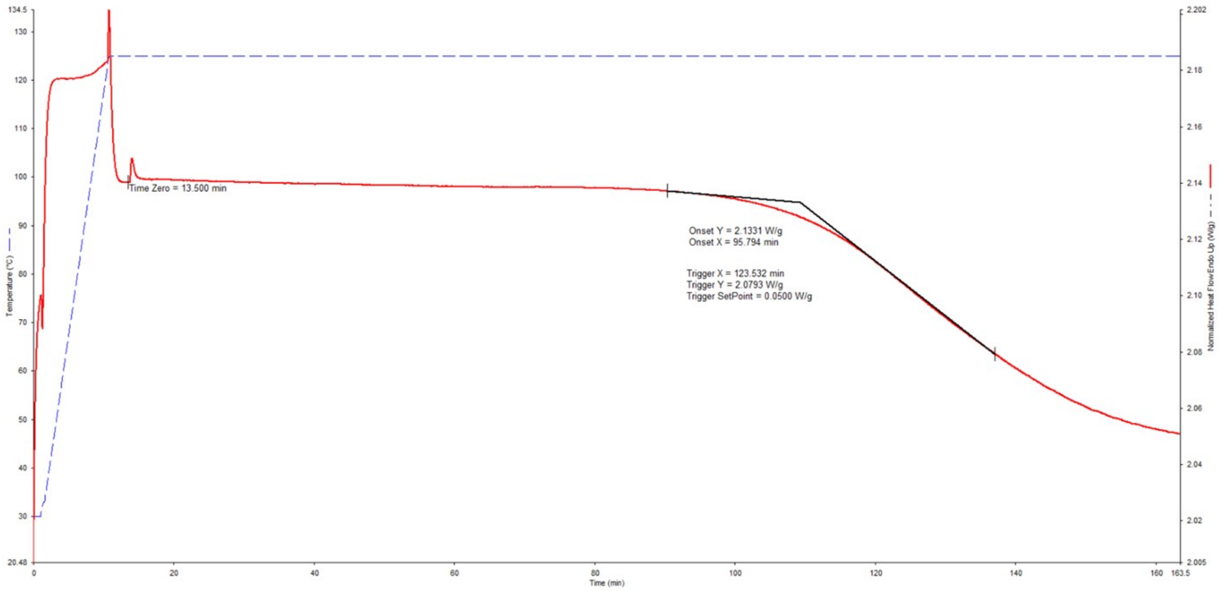

DSC OIT of TMPTO + 5f’
